# Exceptional Early Jurassic fossils with leathery eggs shed light on dinosaur reproductive biology

**DOI:** 10.1093/nsr/nwad258

**Published:** 2023-10-09

**Authors:** Fenglu Han, Yilun Yu, Shukang Zhang, Rong Zeng, Xinjin Wang, Huiyang Cai, Tianzhuang Wu, Yingfeng Wen, Sifu Cai, Chun Li, Rui Wu, Qi Zhao, Xing Xu

**Affiliations:** School of Earth Sciences, China University of Geosciences (Wuhan), Wuhan 430074, China; Key Laboratory of Vertebrate Evolution and Human Origins, Institute of Vertebrate Paleontology and Paleoanthropology, Chinese Academy of Sciences, Beijing 100044, China; University of Chinese Academy of Sciences, Beijing 101408, China; Key Laboratory of Vertebrate Evolution and Human Origins, Institute of Vertebrate Paleontology and Paleoanthropology, Chinese Academy of Sciences, Beijing 100044, China; Guizhou Provincial Museum, Guiyang 550081, China; Guizhou Provincial Institute of Cultural Relics and Archaeology, Guiyang 550001, China; Guizhou Provincial Museum, Guiyang 550081, China; Guizhou Provincial Museum, Guiyang 550081, China; Pingba Institute of Cultural Relics Administration, Anshun 550820, China; Guizhou Provincial Museum, Guiyang 550081, China; Key Laboratory of Vertebrate Evolution and Human Origins, Institute of Vertebrate Paleontology and Paleoanthropology, Chinese Academy of Sciences, Beijing 100044, China; School of Earth Sciences, China University of Geosciences (Wuhan), Wuhan 430074, China; Key Laboratory of Vertebrate Evolution and Human Origins, Institute of Vertebrate Paleontology and Paleoanthropology, Chinese Academy of Sciences, Beijing 100044, China; Centre for Vertebrate Evolutionary Biology, Yunnan University, Kunming 650091, China; Key Laboratory of Vertebrate Evolution and Human Origins, Institute of Vertebrate Paleontology and Paleoanthropology, Chinese Academy of Sciences, Beijing 100044, China

**Keywords:** dinosaur, sauropodomorph, Jurassic, embryo, egg evolution, reproductive behavior

## Abstract

Our understanding of pre-Cretaceous dinosaur reproduction is hindered by a scarcity of evidence within fossil records. Here we report three adult skeletons and five clutches of embryo-containing eggs of a new sauropodomorph from the Lower Jurassic of southwestern China, displaying several significant reproductive features that are either unknown or unlike other early-diverging sauropodomorphs, such as relatively large eggs with a relatively thick calcareous shell formed by prominent mammillary cones, synchronous hatching and a transitional prehatching posture between the crocodilians and living birds. Most significantly, these Early Jurassic fossils provide strong evidence for the earliest known leathery eggs. Our comprehensive quantitative analyses demonstrate that the first dinosaur eggs were probably leathery, elliptical and relatively small, but with relatively long eggshell units, and that along the line to living birds, the most significant change in reptilian egg morphology occurred early in theropod evolution rather than near the origin of Aves.

## Teaser

The earliest dinosaur eggs were probably leathery, relatively small and thin-shelled but theropod eggs become much larger and elongated.

## INTRODUCTION

**Figure 1.**
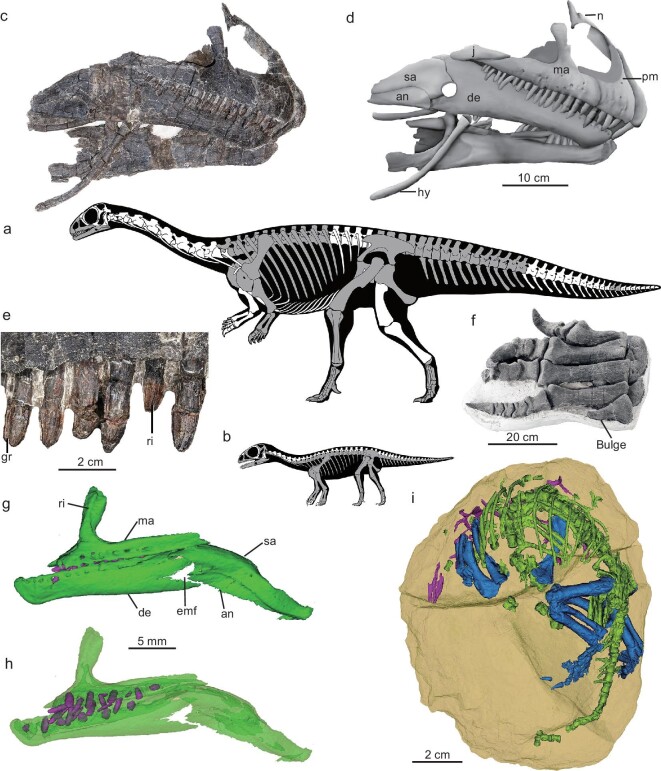
Skeletal morphology of *Qianlong shouhu*. Skeletal silhouettes of the (a) adult and (b) embryo showing preserved bones (in gray) and standing postures. (c) Skull photograph and (d) line drawing in right lateral view, (e) maxillary teeth in right lateral view and (f) right pes in posterior view of GZPM VN001 (adult). (g) Skull normal image and (h) transparency image showing cheek teeth in left lateral view of GZPM VN004-2. (i) 3D reconstruction of the embryo GZPM VN006-1 showing the prehatching posture, with skull elements in purple color; axial skeleton in green; scapula, forelimb and hindlimb in blue. an, angular; de, dentary; emf, external mandibular fenestra; gr, groove; hy, hyoid bone; ma, maxilla; n, nasal; pm, premaxilla; ri, ridge; sa, surangular.

**Figure 2.**
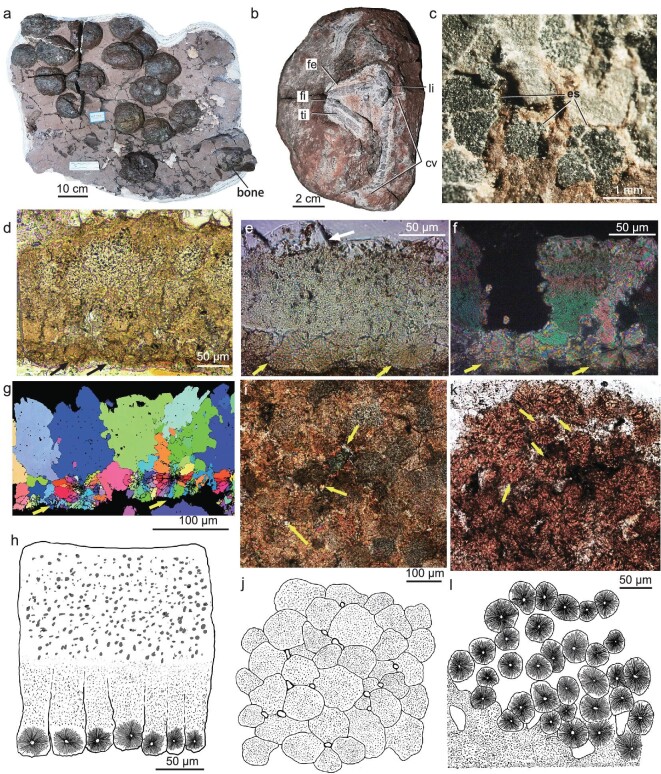
Egg clutch, eggs and eggshell microstructure of *Qianlong shouhu*. (a) Egg clutch GZPM VN005 preserving 16 eggs and a fragmentary bone. (b) The embryonic-skeleton-containing egg GZPM VN006-1. (c) Close-up of eggshell of GZPM VN004-1 showing cracked eggshell. (d and e) Radial thin sections and (h) line drawing of (d) showing the entire eggshell microstructure. (e, arrow) The eggshell covered by secondary calcite is thinner. (f) Radial thin section under polarized light and (g) Inverse Pole figure map under EBSD analysis showing the mammillary cones with nucleation center (yellow arrows). (i) Tangential thin section near the outer surface under PLM and (j) its line drawing showing interlocking eggshell units and elongated and round pores (arrows). (k) Tangential thin section near the inner surface under TLM and (l) its line drawing showing isolated eggshell units with nucleation center (arrows). cv, caudal vertebrae; es, eggshell; fe, femur; fi, fibula; Ii, left ilium; ti, tibia.

Our understanding of dinosaur reproductive biology has greatly improved due to the discoveries of numerous reproduction-related fossils and analyses of data sets compiled from both fossil and neontological data [1–9]. However, fossils relating to dinosaur reproduction are mostly known from Cretaceous deposits, which has sparked debates on whether the rarity of dinosaur eggs in pre-Cretaceous deposits is a preservation/collection artifact or a true evolutionary signal indicating the delayed appearance of thick-shelled eggs, or even hard-shelled eggs, in dinosaur evolution [2,3]. Here we report some exceptional new dinosaur fossils (Figs 1 and 2 and Supplementary Figs S1–S3) significant for reconstructing dinosaur reproduction evolution, and particularly for testing the views mentioned above.

## RESULTS AND DISCUSSION

### Systematic paleontology

Dinosauria Owen, 1842

Saurischia Seeley, 1887

Sauropodomorpha von Huene, 1932

*Qianlong shouhu* gen. et sp. nov.

#### Etymology

The genus name is derived from Mandarin Chinese *Qian* (an alternative name for Guizhou Province where the fossils were collected) + *long* (‘dragon’); the species name *shouhu* means ‘guarding’ in Chinese, referring to the associated preservation of adult skeletal fossils and embryo-containing egg fossils.

#### Holotype

GZPM VN001 is a partial and semi-articulated skeleton (Fig. 1), though the partial skull and mandible are preserved 30 m western to the postcranial skeleton (Supplementary Fig. S1). It is probably an adult individual given the closed neurocentral sutures of all preserved vertebrae.

#### Referred specimens

The specimens comprise two partial semi-articulated skeletons (GZPM VN002 and 003; Supplementary Fig. S2) and five clutches of embryo-containing eggs (GZPM VN004-008; Fig. 2 and Supplementary Fig. S3). All fossils (GZPM VN001-008) are housed at the Guizhou Provincial Museum (GZPM).

#### Locality and horizon

The locality and horizon are Zhuanpo, Pingba District, Anshun City, Guizhou Province, southwestern China and the Lower Jurassic Zhenzhuchong Member (possibly in Sinemurian), Ziliujing Formation [10,11] (Supplementary Fig. S1a–c).

#### Diagnosis

*Qianlong* differs from other sauropodomorphs in the following character states (autapomorphies marked by *): a shallow concavity at the base of the premaxilla nasal process; relatively straight teeth with labiolingually asymmetrical crowns and without denticles; jaw articulation lower than dentary dorsal margin; a short retroarticular process; a very small external mandibular fenestra; well-developed nutritive foramina on the maxillary and dentary, the width of Metacarpal I being greater than its length; Metatarsal V with a strongly expanded proximal end that is four times the mediolateral width of the distal end and with a small bulge on the lateral margin * (Fig. 1 and Supplementary Fig. S2).

#### Description and comparisons

The skull and mandible (Fig. 1) share similar general morphology to those of other early-diverging sauropodomorphs [12]: the snout is relatively long, the large external naris is positioned anteriorly and ventrally, and the dentary has a slightly down-turned anterior end and contributes to more than half of the length of the mandible. There are also a number of derived cranial features, including a relatively posteriorly positioned nasal with a short anteroventral process (also in *Lufengosaurus* [13], *Mussaurus* and other sauropodiforms [14]), a very small external mandibular fenestra (also in *Yizhousaurus* [15], *Riojasaurus* [16] and many sauropodiforms), a high coronoid eminence and ventrally offset jaw articulation (also in *Lufengosaurus, Jingshanosaurus* [17], *Yizhousaurus* and most sauropodiforms), relatively short surangular and angular (also in *Yizhousaurus* [15]), angular posteriorly positioned relative to the mandibular fenestra (also in *Lufengosaurus* and most sauropodiforms) and labiolingually asymmetrical tooth crowns without marginal denticles (also in *Yunnanosaurus, Irisosaurus* and many sauropodiforms) and with several longitudinal ridges on the labial surface (also in *Chuxiongsaurus* [18]).

In the postcranial skeleton, morphological features shared with other early-diverging sauropodomorphs (Supplementary Fig. S2) include three sacral vertebrae; an elongated, laterally arched scapula; a relatively short humerus with a well-developed deltopectoral crest; a very stout Metatarsal I; a relatively small ilium with a short pre-acetabular process and a long pubic peduncle; a long pubis with a large obturator foramen; a long ischial shaft with a subtriangular cross section; and a robust sigmoid femur longer than the tibia. Derived postcranial features include anterior dorsals with a transversely expanded dorsal end of the neural spine (also in *Yizhousaurus* [15] and some other sauropodiforms), short anteriormost caudals (also in sauropods [12]), a short manus (also in *Yizhousaurus* [15], *Jingshanosaurus* [19] and many other sauropodiforms [20]), robust manual digits (also in *Lufengosaurus* [21] and some sauropodiforms such as *Yizhousaurus* and *Mussaurus* [22]), a relatively long pubic apron, Pedal Ungual I longer than all nonterminal phalanges (also in *Jingshanosaurus* [19] and other sauropodiforms [23]) and a short Metatarsal V with a strongly expanded proximal end and a lateral bulge [unknown in any other sauropodomorphs (Fig. 1f)] (see Supplementary Data for more description and comparisons). Our phylogenetic analysis places *Qianlong* as the sister taxon to *Yunnanosaurus* near the base of Sauropodiformes (Supplementary Fig. S4).

### Embryos and growth

**Figure 3.**
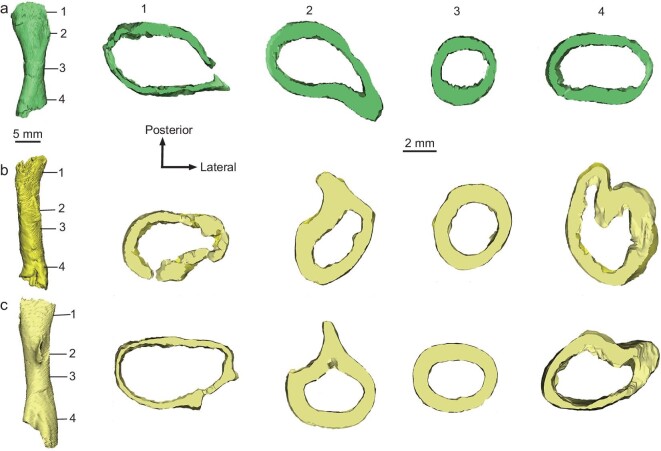
Cross sections of the limb bones of *Qianlong* embryos derived from CT reconstruction. (a and b) GZPM VN006-2 showing that the forelimb is only slightly thinner than the hindlimb in *Qianlong* as indicated by the cross-section data. (a) Humerus in anterior view. (b) Femur in posterior view. (c) GZPM VN004-2 femur in posterior view. GZPM VN004-2 is similar in size to GZPM VN006-2 as indicated by the cross-section data.

Six embryos from two egg clutches display long bones through either direct exposure or CT imaging, and have a large medullary cavity and a very spongy cortex. Microstructures such as numerous primary cavities and abundant osteocyte lacunae (Supplementary Fig. S5) suggest fast growth [24]. These embryos are probably in their late developmental stage as indicated by nearly full occupation of the egg space by the skeleton (Fig. 1i). They display a transitional prehatching posture between the crocodilians and living birds: the head is near the pole and the hindlimbs are only partially crouched (Fig. 1i) as late-stage embryos of *Massospondylus* [25] and extant crocodilians [26,27], but the back is curved along the pole and the hip is near the central portion of the egg as in those of early [28] and living birds [29] as well as possibly oviraptorids [30] and troodontids [31] (but see [27] for a different interpretation of oviraptorid prehatching posture). All embryos are similar in the ossification degree and size (Fig. 3, Supplementary Fig. S6 and Supplementary Table S1), suggesting that *Qianlong* has a synchronous hatching strategy and synchronous breeding in this colonial nesting site.

The embryos display several characteristics that are shared with their adult counterparts (Fig. 1g–i and Supplementary Fig. S6): the maxillary dorsal process deflected distinctly from the anterior ramus at a large angle (∼70 degrees), the dentary proportionally long (∼60% of the mandibular length) and posteriorly bifurcated, the external mandibular fenestra proportionally small, four premaxillary teeth, relatively straight tooth crown, well-developed nutritive foramina on the maxillary and dentary, the pubic apron long (>30% of the pubic length), the ilium with a short pre-acetabular process and a relatively long postacetabular process, a smooth convex dorsal margin, a long pubic peduncle and the prominent plate-like femoral fourth trochanter relatively proximally and medially positioned.

However, the embryos also display some differences from the adults. Some of these differences are ontogenetic variations also seen in other sauropodomorphs [14,32,33], including proportionally longer skull and mandible, a more vertical anterior margin of the premaxilla and fewer teeth in the embryos. Other differences, such as maxillary anterior ramus shallow and subtriangular in the embryos but deep in adults, the presence in the embryos but absence in the adults of a narrow ridge along the maxillary posterodorsal ramus (Fig. 1g) and retroarticular process long in the embryos but short in the adults, have not been reported previously in the ontogenetic series of other sauropodomorphs [14,32,33].

Some proportional features (Supplementary Table S1) indicate that the embryos have proportionally longer forelimbs and larger shoulder girdles than the adults—a phenomenon is also seen in *Massospondylus* and *Mussaurus* [34]. Limb cross-section data display a similar pattern: humeral cross section is close in size to the femoral one in embryos (Fig. 3), but the difference is huge in adults. *Qianlong* thus may have been quadrupedal at hatching. Ontogenetic shifting from quadrupedalism to bipedalism has been proposed for early-diverging sauropodomorphs based on data gathered in both limb proportions [25] or the body's center of mass [35]. Our allometric growth analysis provides support for this proposal, and specifically the humerus displays a negative allometry in the growth of early-diverging sauropodomorphs but near isometry or even a positive allometry in the growth of sauropods (Fig. 6a). This suggests that early-diverging sauropodomorphs are similar to sauropods in body plan at their early ontogenetic stages, but differ in the growth pattern, which leads to the different body proportions at later ontogenetic stages.

### Nesting and eggs

The five egg clutches containing the same type of eggs are distributed in a small area of ∼15 m^2^ and the three adult/subadult skeletons are preserved with a distance to the egg clutches ranging from 1 to 3 meters. All fossils except GZPM VN002 (yellow surface color) are from purple silty mudstone and the latter from a layer of purple siltstone ∼0.7 m above the former fossil bed layer (Supplementary Figs S7–S11). The fossil-bearing beds are featured by massive fine brown mudstone, abundant calcium carbonate nodules, along with slickensides and weak color mottling, indicating that they are paleosol origins and floodplain deposits of low energy (see more details in Supplementary Data). The general taphonomical and sedimentary features are similar to those of the fossil-bearing beds of several other early-diverging sauropodomorphs [36,37] that have been suggested to possess such reproductive behaviors as colonial nesting and site fidelity. The preserved *Qianlong* adult skeletons display a prostrating posture similar to that of some *Plateosaurus* fossils that were interpreted as resulting from miring [38].

The preserved egg clutches vary in size, with the smallest clutch containing 3 eggs and the largest with 16 eggs (Fig. 2a and Supplementary Fig. S3), and are much smaller in size than the largest known clutch of *Massospondylus* and *Mussaurus* containing 34 eggs and 30 eggs, respectively [36,39], though the possibility of incomplete preservation leading to the small clutch sizes could not be dismissed. Most eggs are generally elliptical in outline. However, many small pits are observed on the outer surface, leading to a somewhat irregular shape of the eggs (Fig. 5c). *Qianlong* eggs have a diameter of ∼11.5 cm × 9.4 cm, which is more similar in size to those of sauropods (ranging from 9 to 23 cm in egg diameter) [1,40] than to those of other early-diverging sauropodomorphs such as *Massospondylus* and *Mussaurus* (∼6–7 cm in egg diameter) [25,41].

The eggs have a calcareous eggshell layer of 160 ± 26 μm on average (*n* = 30, ranging from 115 to 230 μm, Fig. 2c–e). The irregular outer surface indicates eggshell weathering and thus the original *Qianlong* eggshell possibly is even thicker. *Qianlong* thus has a calcareous eggshell that is considerably thicker than that of other known early-diverging sauropodomorphs such as *Massospondylus* (80–100 μm) [2], much thicker than the calcareous layer of all known soft-shelled eggs (usually <60 μm) [42], but much thinner than that of most other non-avian dinosaur eggs (400–4750 μm) [43].

**Figure 4.**
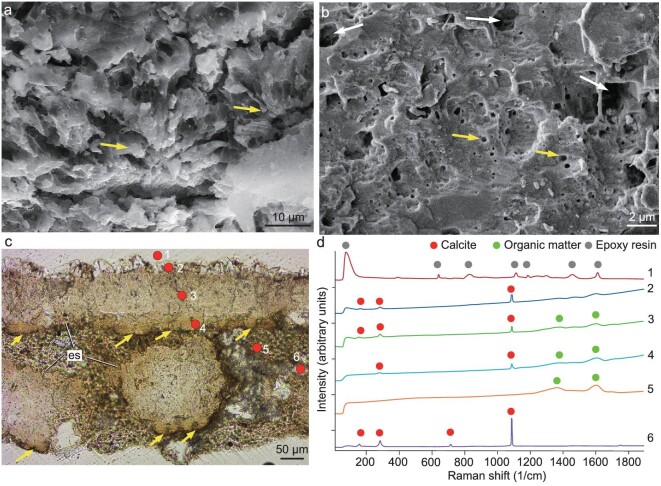
Microstructure and Raman spectra of *Qianlong* eggshell. (a) Radial section under SEM showing the nucleation centers of two eggshell units (arrows). (b) Radial section under SEM showing numerous cavities (white arrows) and tiny vesicles (yellow arrows) throughout the whole eggshell. (c) Radial thin section of the eggshell under normal light showing eggshells (es) and nucleation centers (yellow arrows). (d) Raman point spectra were acquired at the positions labeled with the red dots in (c): 1. Epoxy resin; 2. Secondary calcite on the outer surface of the eggshell; 3, 4. Eggshell; 5. Organic matters in sediments; 6. Calcite in sediments.

In radial thin sections, the eggshell consists of interlocking columnar eggshell units with a height-to-wide ratio of ∼2 : 1 to 5 : 1 (Fig. 2d, e and h) and the boundaries between the interlocking eggshell units are irregular (Fig. 2f and g). Round and elongated pores occasionally appear between adjacent eggshell units (Fig. 2i and j). Quantitative analysis indicates that *Qianlong* had relatively high eggshell porosity (Supplementary Table S5) and, by combining the egg mass data, our analysis indicates that *Qianlong* had covered nests (Supplementary Fig. S13) as in most non-pennaraptoran archosaurs [44]. Towards the inner surface, the eggshell units become isolated from each other (Fig. 2k and l). At the inner surface of the eggshell, the mammillary cone exhibits a radial arrangement of calcite grains and a small rounded nucleation center (Figs 2d–h, k, l and 4a, c), as in turtles and all other dinosaurs including birds [45] (Supplementary Figs S14 and S15). Electron backscatter diffraction (EBSD) imaging shows that the mammillary cones continue by large vertical prism-shaped calcite grains in the outer portion of the eggshell (Fig. 2g) as in typical dinosaur eggshells (e.g. *Placoolithus*, Supplementary Fig. S14f); scanning electron microscope (SEM) imaging reveals that there are numerous tiny vesicles in the calcite crystals (Fig. 4b), resembling those of Cretaceous dinosaur eggshells [46,47].

The presence of a calcareous layer is further supported by our chemical analyses. Energy-dispersive spectroscopy (EDS) indicates that *Qianlong* eggshell mainly consists of C, O and Ca (Supplementary Table S12) and Raman spectroscopy also detects the calcite signal from the *Qianlong* eggshell (Fig. 4c and d). However, organic matters are detected in the eggshells and the surrounding matrix and, interestingly, the Raman spectra obtained from the *Qianlong* eggshell are similar in calcite and organic matter signal patterns to those from *Mussaurus* eggshell [3]. This suggests that the signal pattern of organic matters revealed from *Mussaurus* eggshell [3] is not reliable evidence for soft-shelled eggs. The transmitted light microscopy (TLM), polarized light microscopy (PLM) and EBSD images confirm that *Qianlong* eggshell resembles other dinosaur eggshells at a microstructural level, though the microstructure is less well preserved in *Qianlong* eggshell than in most Cretaceous dinosaur eggs that have been studied from this perspective.

**Figure 5.**
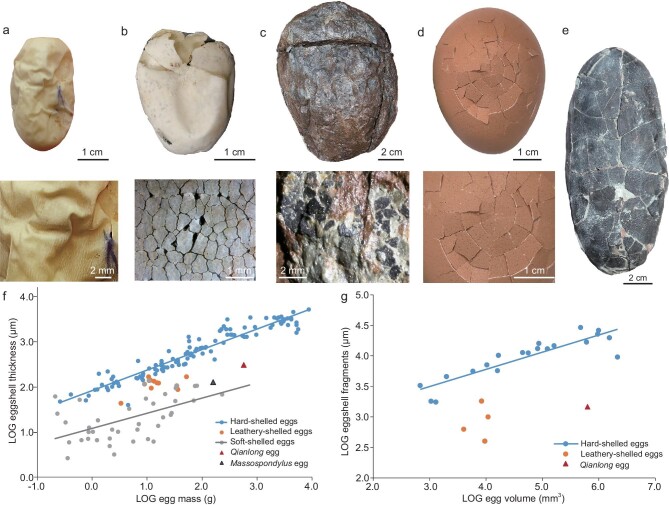
Photographs and scaling of diapsid eggshell type, thickness and fragment size. (a) Soft eggshell. *Pantherophis guttatus*, strongly folded eggshell without broken fragments. (b and c) Leathery eggshells. (b) *Pseudemys nelson*, moderately folded eggshell with small fragments (∼1–2 mm). (c) *Qianlong shouhu*, showing rugose eggshell surface with small fragments (∼2 mm). (d and e) Rigid eggshells. (d) *Gallus gallus domesticus*, showing large eggshell fragments. (e) *Elongatoolithus magnus* (CUGW EH023), showing large eggshell fragments. (f) Plot of LOG egg mass vs. LOG eggshell thickness in diapsids. (g) Plot of LOG egg volume vs. LOG eggshell fragment sizes. Both charts support that *Qianlong* probably laid leathery eggs.

There are several lines of evidence supporting the identifications of the eggs of *Qianlong* and probably other early-diverging sauropodomorphs as leathery ones. First, their eggs have a shell thickness that is similar to that of extant leathery eggs (usually 70–200 μm) (see also Supplementary Table S8). Second, *Qianlong* eggs display sharp edges of broken shells (Fig. 2c), as in some leathery eggs of extant turtles and hard-shelled eggs (Fig. 5b and c), and they further resemble leathery eggs in having small eggshell pieces when eggs are broken (Fig. 5b). Finally, our statistic analyses of relative eggshell thickness (Fig. 5f) and the relative size of eggshell pieces (Fig. 5g) demonstrate that *Qianlong* eggs are more similar to leathery eggs than to either hard-shelled eggs or soft-shelled eggs. In summary, the relatively thin eggshell thickness compared with the egg mass, the rugose egg surface, the slightly irregular egg shape and the strongly pieced eggshells provide strong support for the leathery nature of eggs of *Qianlong* and probably other early-diverging sauropodomorphs (Fig. 5 and Supplementary Fig. S3c).

### Evolution of selected reproduction features

**Figure 6.**
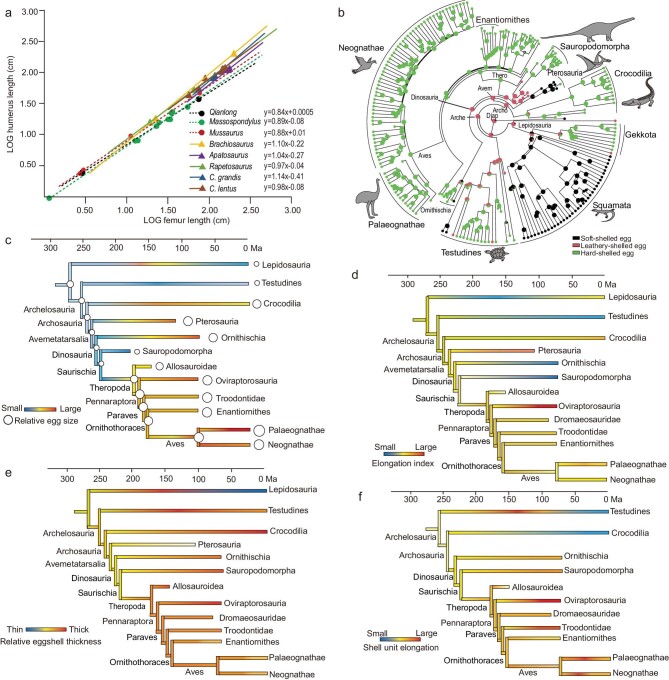
Sauropodomorph growth strategies and diapsid reproduction evolution. (a) Regression analysis shows growth trajectories of selected sauropodomorphs. (b) Eggshell type ASR under hierarchal Bayes framework with new scoring and ARD model (two rate classes; using majority rule consensus tree of run1 in the first dating analysis). A simplified time-calibrated diapsid tree showing the (c) egg-size evolution, (d) egg elongation index, (e) eggshell-thickness evolution and (f) eggshell unit based on our ASR analyses (Supplementary Figs S18–S25). Color changes from blue to red indicate an increase in all values. Arche, Archelosauria; Archo, Archosauria; Avem, Avemetatarsalia; Diap, Diapsida; Thero, Theropoda.

To better understand the evolution of avian reproductive biology, we performed ancestral-state reconstruction (ASR) analyses to trace the evolution of egg size and shape as well as eggshell type, microstructure and thickness. The new data sets were compiled from several recent studies [2,3,6,48] but with significant expansion, and they contain 210 diapsid taxa with both ascertained systematic positions and relevant reproduction data for our analyses (Fig. 6 and Supplementary Figs S16 and S17).

Our egg-size ASR analyses show that the evolution of relative egg size (egg volume relative to adult body mass) displays a decreasing trend from the base of the Diapsida to that of the Saurischia, followed by an egg-size-increase trend from early theropods to the crown bird node (Fig. 6c and Supplementary Figs S18 and S19). The former trend leads to plesiomorphically smaller eggs in Dinosauria (with the exception of turtles) and the latter to plesiomorphically larger eggs in Aves compared with all other diapsid groups, though the most significant egg-size increase occurred early in theropod evolution. Meanwhile, an egg-size-increase trend is also seen in some lineages of lepidosaurs, turtles, crocodilians, pterosaurs, ornithischians, oviraptorosaurians, palaeognaths and neognaths, though only the trend in the oviraptorosaurian and paleognath evolution has been relatively well supported by the data. An egg-size-decrease trend has also been detected in some lepidosaur lineages and in the evolution of sauropodomorphs and neognaths, leading to some of the smallest eggs found in some sauropodomorph clades, among the known archosaurian clades.

Egg-shape evolution displays a different pattern from size evolution. Egg shape (measured by using the elongation index) is generally conservative along the line to living birds in diapsid evolution: nearly all nodes (e.g. the Diapsida, Archelosauria, Archosauria, Ornithodira and Aves) except several non-avialan dinosaurian nodes display an egg elongation index of 0.13–0.15 (Fig. 6d and Supplementary Figs S20 and S21). This lack of shape change is also seen in most crown bird clades, in stark contrast to most reptilian groups and their subclades that display either a much smaller or a much larger egg elongation index (Fig. 6d and Supplementary Figs S20 and S21). The former is seen in sauropodomorphs, ornithischians, turtles and a few lepidosaur clades, which have nearly rounded eggs, and the latter is present in non-avialan theropods, pterosaurs, crocodilians and some lepidosaur clades, which show much more elongated eggs. The theropod egg elongation leads to the most elongated diapsid eggs in oviraptorosaurs, but would later be reversed to the plesiomorphic, slightly elongated eggs that are inherited by all crown bird clades.

Similarly, the relative eggshell thickness (eggshell thickness relative to egg volume) also displays a relatively complex evolutionary pattern (Fig. 6e and Supplementary Figs S22 and S23). Along the line to extant birds in archosaur evolution, there is an evolutionary trend of eggshell-thickness decrease from the base of the group to that of the Saurischia, followed by a significant eggshell-thickness increase early in theropod evolution. An evolutionary trend of eggshell-thickness decrease is also seen in neognaths, paleognaths, enantiornithines, some turtle lineages and some lepidosaur lineages whereas the reverse is seen in sauropodomorphs, ornithischians, some lineages of oviraptorosaurian theropods, crocodilians, turtles and lepidosaurs.

Although the homologous relationships of diapsid eggshells are highly debated [48,49], the eggshell units are widely accepted to be the basic components of the calcareous shell layer [50]. Thus, the eggshell unit evolution is key to our understanding of diapsid egg evolution. Our ASR analyses of the eggshell-unit-elongation index (EI, the ratio of eggshell unit length to width) show that there is an evolutionary trend of eggshell unit elongation from the base of Archelosauria to that of Pennaraptora, and along some lineages of neognath and paleognath birds, oviraptorosaurian theropods, sauropodomorphs and turtles. Meanwhile, an opposite trend is present in enantiornithines and some paleognath, neognath, turtle, crocodilian and sauropodomorph lineages (Fig. 6f and Supplementary Figs S24 and S25). Among diapsids, some oviraptorosaurian and troodontid clades have the most elongated eggshell units while some crocodilian and turtle clades have the shortest ones.

Extant amniotic eggs are traditionally classified into soft-shelled, leathery and hard-shelled ones [45,50–52] (see Supplementary Data), though this classification oversimplifies the great variety of eggshell morphologies [48]. Nevertheless, a calcareous layer formed by eggshell units characterizes both leathery and hard-shelled eggs, the appearance of which represents a key event in egg evolution [2]. Eggs of early-diverging sauropodomorphs are only known in three species, but are controversial in their morphologies. The calcareous layer of *Massospondylus* eggshells seems to be composed of columnar structural units, but whether they are original eggshell units is uncertain due to severe recrystallization of the eggshells [2]; the known *Lufengosaurus* eggshell is composed of crown-shaped eggshell units with radially arranged calcite crystals, comparable to the inner portion of the *Qianlong* eggshell (Fig. 2e–h), suggesting that the preserved *Lufengosaurus* eggshells likely represent eggshell interior with the exterior being weathered away; the soft-shelled nature of *Mussaurus* eggs has been inferred based on the chemical composition revealed by Raman spectra [3], but our comparative chemical data from *Qianlong* eggs support the argument that the chemical evidence for the presence of soft-shelled eggs in *Mussaurus* needs be re-evaluated [51]. Our eggshell type ASR analyses incorporate new data from *Qianlong* and other key taxa and are conducted with consideration of temporal and character scoring uncertainty—issues that might have affected significantly the ASR analyses [48]. For example, to consider temporal and character scoring uncertainty in eggshell type ASR, we respectively used 22 different time-scaled trees and two different criteria for identifying eggshell types (Supplementary Data and https://figshare.com/s/14374b47d33d96aef963). These analyses produced similar and robust results, and recovered pterosaurs as ancestrally soft-shelled and Archelosauria, Testudines, Archosauria, Avemetatarsalia, Dinosauria and Saurischia as ancestrally leathery eggshell in most results (Fig. 6b and Supplementary Figs S26–S32).

Some results of our analyses are different from those of some previous studies [4,8,48]. For example, the first dinosaur eggs were suggested to be either hard [52] or soft [3]; other studies suggest that the major changes in the avian reproduction system have occurred incrementally, including an evolutionary trend of increasing egg size along the line to crown birds [4,8] and an increasing eggshell thickness after the Early Jurassic corresponding to an increase in global atmospheric oxygen during the same temporal period [2]. However, our study provides strong evidence for the leathery eggs in early-diverging sauropodomorphs and suggests a leathery eggshell origin for major diapsid subclades including the Dinosauria; our study also reveals a complex evolutionary history of egg size and eggshell thickness along the line to crown group birds. Most significantly, our analyses indicate that dinosaurs ancestrally had distinct eggs compared with other reptilian groups, which were relatively small, moderately elongated and thin-shelled but with moderately elongated eggshell units, and probably leathery. Along the line to living birds in dinosaur evolution, the most significant change in egg morphology occurred early in theropod evolution and stem birds closely resemble non-avialan theropods and particularly non-avialan maniraptorans in egg morphology. Except for relatively large egg size, extant birds either inherited their theropod ancestral condition (e.g. relatively thick eggshell and long eggshell units) or reversed to the primitive condition (e.g. relatively short eggs) in egg morphology. The discovery of *Qianlong* and our analyses clearly show that the evolution of the dinosaur reproduction system is a complex process and the evolution of some important reproduction features such as egg size and shape and eggshell thickness are more likely to have been driven by multiple factors rather than by a single factor such as phylogeny, development or environment.

## CONCLUSION

This study reports some exceptional fossils of a new early-diverging sauropodomorph dinosaur, *Qianlong shouhu* gen. et sp. nov., from the Lower Jurassic Ziliujing Formation of southwestern China and makes several novel findings pertaining to diapsid reproduction biology: (i) The early-diverging sauropodomorph *Qianlong* has relatively large eggs with a relatively thick calcareous shell formed by prominent mammillary cones compared with other early-diverging sauropodomorphs, a transitional prehatching posture between the crocodilians and living birds, and a synchronous hatching pattern. (ii) *Qianlong* and other early-diverging sauropodomorphs have leathery eggs. (iii) ASR analyses demonstrate that the first dinosaur eggs were probably leathery, elliptical and relatively small, but with relatively long eggshell units, and that egg shape is generally conservative among extant birds and the most significant change in reptilian egg morphology occurred early in theropod evolution. These findings are significant to our knowledge of the reproductive biology of diapsids, particularly of dinosaurs.

## METHODS

### Phylogenetic analyses

We analyse a recently published data set for sauropodomorph phylogeny [36] with *Qianlong* added in Supplementary Table S3. A total of 80 taxa and 419 characters were included in the data matrix. The analysis was run using TNT V. 1.5 [53] with the maximum trees set to 10 000. All the characters were equally weighted and 41 additive characters were set [36]. A heuristic search using a new technologies algorithm was used, with 100 hits to minimum length, followed by tree swapping using TBR (tree bisection reconnection) on the trees in memory (see details in Supplementary Data).

### Computed tomographic scan and 3D reconstruction

Four embryo-containing eggs were scanned using a Phoenix Vtomex M micro-computed tomography scanner at the Yinghua Inspection and Testing Shanghai Company and the Key Laboratory of Vertebrate Evolution and Human Origin of Chinese Academy of Sciences, IVPP. Scanning parameters were set to a tube voltage of 180–200 KV and a current of 100–150 μA with a voxel size of 22.49–35.044 μm^3^ (Supplementary Table S13). Reconstruction of radiographs was performed using the software Mimics 17 at the IVPP.

### Raman analyses

*In situ* Raman microspectroscopy was conducted using a WITec α300 Confocal Raman system coupled with a Peltier cooled EMCCD detector at the State Key Laboratory of Biogeology and Environmental Geology, China University of Geosciences (Wuhan). Laser excitation was provided at 532 nm with 7.9 mW of output laser power at the surface of the sample. Each sample was scanned in the spectral range from 0 to 4000 cm^−1^. The integration time for each spectrum was 3 s and the number of accumulations was 10. Software WITec Project Five 5.1 Plus was used to process the Raman spectra.

### Allometric growth analysis

Allometric growths of three non-sauropod sauropodomorph species and five sauropod species were investigated by using a bivariate plot of the humeral length relative to the femoral length of 33 individuals representing different ontogenetic stages of these eight species (Supplementary Table S4). Unitary linear regression analyses were performed to detect the allometric relationships between the log-transformed humerus and femur in Excel (2016).

### ASR analyses

The sampled taxa cover major reptilian clades, including crocodilians, birds, non-avian dinosaurs, pterosaurs, turtles, lepidosaurs and choristoderes (Supplementary Tables S9 and S10) and in total the data sets include 210 taxa. Here we use two criteria (new scoring and ratio scoring) to do ASR analysis for they are widely used in eggshell type definition [48]. We assembled an informal supertree manually in Mesquite v3.6.1 and used hidden Markov chain model that considers rate heterogeneity and performed ASR analyses of eggshell type under a Hierarchal Bayesian framework in RevBayes.1.1.1 using all rate different model (ARD) under two hidden rate classes. Relative egg size and relative eggshell thickness were determined by using phylogenetic linear regression with Log10 transformed data. Residuals taken from the regression models were used to indicate the relative egg size and relative eggshell thickness. The phylogenetic linear regression analyses were performed in R 4.1.3 with the package ‘caper’ and Pagel's Lambda was used to consider the phylogenetic signal (Supplementary Fig. S17). Identically, we performed ASR on log 10 transformed egg EI (ratio of egg long axis to short axis) and eggshell unit index (ratio of eggshell unit depth to width) with supertrees rescaled by Pagel's Lambda by using the function ‘fastAnc’ (see detailed in Supplementary Data).

## Supplementary Material

nwad258_Supplemental_File
