# Supplementary material for: Exceptional Early Jurassic fossils with leathery eggs shed light on dinosaur reproductive biology
Source: Natl Sci Rev. 2023 Oct 9;11(6):nwad258. doi: 10.1093/nsr/nwad258 (PMC11067957; doi:10.1093/nsr/nwad258)
Supplement: nwad258_Supplemental_File [file nwad258_supplemental_file.docx]

Supplementary Information to

**Exceptional Early Jurassic fossils with leathery eggs shed light on dinosaur reproductive biology**

Content

[Supplementary methods 4](#_Toc148474098)

[Discovery, excavation and preparation of *Qianlong* fossils 12](#_Toc148474099)

[Geological setting, sedimentology and taphonomy 13](#_Toc148474100)

[Additional morphological description 16](#_Toc148474101)

[Amniotic egg type and *Qianlong* eggshell 20](#_Toc148474102)

[Supplementary Figures 22](#_Toc148474103)

[Supplementary Figure 1. Location, geology and stratigraphy of Pingba dinosaur site and distribution of *Qianlong* fossils within the quarry. 22](#_Toc148474104)

[Supplementary Figure 2. Selected *Qianlong* skeletal elements. 23](#_Toc148474105)

[Supplementary Figure 3. Egg clutches collected from the Pingba dinosaur site. 24](#_Toc148474106)

[Supplementary Figure 4. A reduced consensus tree of 2436 most parsimonious trees produced by a parsimonious analysis of a dataset modified from ref. [1] with a posteriori pruning of thirteen unstable taxa 25](#_Toc148474107)

[Supplementary Figure 5. Bone histology of the embryo GZPM VN004. 26](#_Toc148474108)

[Supplementary Figure 6. Selected embryonic elements. 27](#_Toc148474109)

[Supplementary Figure 7. Collecting dinosaur fossils at the Pingba dinosaur site in 1999. 28](#_Toc148474110)

[Supplementary Figure 8. Filed photographs of paleosols from the Lower Jurassic Ziliujing Formation of Pingba Section. 29](#_Toc148474111)

[Supplementary Figure 9. Micromorphological features in paleosols from the Lower Jurassic Ziliujing Formation of Pingba Section. 30](#_Toc148474112)

[Supplementary Figure 10. Lithostratigraphic logs and δ^13^C_org_, δ^13^C (VPDB), and δ^18^O values from Pingba sections. 31](#_Toc148474113)

[Supplementary Figure 11. Plots of δ^13^C vs. δ^18^O values in carbonate nodules from Pingba sections (a) and fossil beds (b). 32](#_Toc148474114)

[Supplementary Figure 12. Bivariate plot of calcified eggshell thickness and total eggshell thickness in 22 living birds (Supplementary Table 6) 32](#_Toc148474115)

[Supplementary Figure 13. Bivariate plot of eggshell porosity and egg mass in living and extinct archosaur taxa/ootaxa. 33](#_Toc148474116)

[Supplementary Figure 14. Comparison of eggshell microstructure between *Qianlong* and other dinosaur eggs. 34](#_Toc148474117)

[Supplementary Figure 15. Comparison of eggshell microstructure between *Qianlong* and *Deinonychus* through tangential section. 35](#_Toc148474118)

[Supplementary Figure 16. Time-scaled supertree (majority rule consensus tree of run1 in the first dating analysis) of 210 diapsid taxa used for the character ancestral state reconstruction analysis in this study. 37](#_Toc148474119)

[Supplementary Figure 17. Time-scaled supertree (majority rule consensus tree of run2 in the first dating analysis) of 210 diapsid taxa used for the character ancestral state reconstruction analysis in this study. 39](#_Toc148474120)

[Supplementary Figure 18. Relative egg size ASR with majority rule consensus tree of run1 in the first dating analysis. 41](#_Toc148474121)

[Supplementary Figure 19. Relative egg size ASR with majority rule consensus tree of run2 in the first dating analysis. 42](#_Toc148474122)

[Supplementary Figure 20. Egg elongation index ASR with majority rule consensus tree of run1 in the first dating analysis. 44](#_Toc148474123)

[Supplementary Figure 21. Egg elongation index ASR with majority rule consensus tree of run2 in the first dating analysis. 46](#_Toc148474124)

[Supplementary Figure 22. Relative egg thickness ASR with majority rule consensus tree of run1 in the first dating analysis. 48](#_Toc148474125)

[Supplementary Figure 23. Relative egg thickness ASR with majority rule consensus tree of run2 in the first dating analysis. 50](#_Toc148474126)

[Supplementary Figure 24. Eggshell unit index ASR with majority rule consensus tree of run1 in the first dating analysis. 51](#_Toc148474127)

[Supplementary Figure 25. Eggshell unit index ASR with majority rule consensus tree of run2 in the first dating analysis. 52](#_Toc148474128)

[Supplementary Figure 26. Summary of ancestral eggshell type with the maximum posterior probabilities in all ASR analyses. 53](#_Toc148474129)

[Supplementary Figure 27. Egg type for the Dinosauria node based on ASR analyses using Hierarchal Bayes framework with ARD model (2 rate classes) 54](#_Toc148474130)

[Supplementary Figure 28. Eggshell type ASR under hierarchal Bayes framework with new scoring and ARD model (2 rate classes; using majority rule consensus tree of run1 in the first dating analysis). 55](#_Toc148474131)

[Supplementary Figure 29. Eggshell type ASR under hierarchal Bayes framework with new scoring and ARD model (2 rate classes; using majority rule consensus tree of run2 in the first dating analysis). 56](#_Toc148474132)

[Supplementary Figure 30. Eggshell type ASR under hierarchal Bayes framework with ratio scoring and ARD model (2 rate classes; using majority rule consensus tree of run1 in the first dating analysis). 57](#_Toc148474133)

[Supplementary Figure 31. Eggshell type ASR under hierarchal Bayes framework with ratio scoring and ARD model (2 rate classes; using majority rule consensus tree of run2 in the first dating analysis). 58](#_Toc148474134)

[Supplementary Figure 32. Phylogenetic linear regression between log_10_ transformed egg volume and body mass, and between log_10_ transformed eggshell thickness and egg volume. 59](#_Toc148474135)

[Supplementary Tables 60](#_Toc148474136)

[Supplementary Table 1. Main measurements of the adult and embryo of *Qianlong* 60](#_Toc148474137)

[Supplementary Table 2. Results of δ^13^C_org_, δ^13^C (VPDB), δ^18^O (VPDB) analyses in Pingba Section and fossil beds. 62](#_Toc148474138)

[Supplementary Table 3. Character coding of *Qianlong shouhu.* 63](#_Toc148474139)

[Supplementary Table 4. Collection of limb bone measurements in basal sauropodomorphs and sauropods. 63](#_Toc148474140)

[Supplementary Table 5. List of variables and equations used for this study and analysis results. 65](#_Toc148474141)

[Supplementary Table 6. Collections of eggshell thickness in 22 extant birds. 66](#_Toc148474142)

[Supplementary Table 7. Measurements of average eggshell fragments length in some extant and extinct taxa. 67](#_Toc148474143)

[Supplementary Table 8. Collection of leathery egg data of extant reptiles for analyzing eggshell type. 68](#_Toc148474144)

[Supplementary Table 9. Dataset used for reproduction evolution analyses. 69](#_Toc148474145)

[Supplementary Table 10. Sample size for each major clade in this study. 86](#_Toc148474146)

[Supplementary Table 11. Differences of eggshell type Scoring between Legendre et al. (*48*) and this study 86](#_Toc148474147)

[Supplementary Table 12. Comparison of EDS results of eggshell for *Qianlong* and *Gallus gallus domesticus* 87](#_Toc148474148)

[Supplementary Table 13. Scanning parameters of four embryos of *Qianlong* in this study. 87](#_Toc148474149)

[Supplementary Table 14. References used to associate egg fossils with known adult taxa in this study. 88](#_Toc148474150)

[References 93](#_Toc148474151)

## Supplementary methods

**Phylogenetic analyses.** To determine the systematic position of *Qianlong*, we analyzed a recently published dataset for sauropodomorph phylogeny [1] with *Qianlong* added in (Supplementary Table 3). A total of 80 taxa and 419 characters were included in the data matrix. The analysis was run using TNT V. 1.5 [2] with the maximum trees set to 10, 000. All the characters were equally weighted and 41 additive characters were set [1]. A heuristic search using new technologies algorithms was used, with 100 hits to minimum length, followed by tree swapping using TBR on the trees in memory. The analysis recovered 2,436 most parsimonious trees with a tree length of 1,707 steps (Consistency Index = 0.286; Retention Index = 0.651), the strict consensus tree of which displays a poor resolution at the base of the Sauropodomopha. After pruning thirteen wild-card taxa, we produced a highly resolved reduced consensus tree, which posits *Qianlong* as the sister taxon to *Yunnanosaurus huangi* near the base of Sauropodiformes (Supplementary Fig. 4) and they are supported by three synapomorphies, including teeth crowns not overlapping, the absence of denticles on check teeth and fourth trochanter along the mediolateral axis of the femur centrally located. The clade of Sauropodiformes was supported by three synapomorphies, including the straight anterolateral margin of the deltopectoral crest of the humerus, at least distal phalanges as wide as long in non-terminal pedal phalanges, and the absence of serrations on premaxillary teeth. A bootstrap analysis was carried out with 1000 replications and the “maxtrees” option in TNT was set to 1000. Bootstrap values are low throughout much of the tree (Supplementary Fig. 4).

**Histological analysis**. Bone and eggshell histological samples were taken from two embryo-containing eggs from GZPM VN004 (Supplementary Fig. 3b). The cross-section of the preserved radius (GPZM VN004-1) near the mid-diaphysis was taken to make a thin section. Eggshell samples were taken directly from the broken shell pieces by tweezers. Both radial and tangential sections were made for observation and comparison. The preparation of thin sections was carried out at the Institute of Vertebrate Paleontology and Paleoanthropology (IVPP) using the EXAKT-cutting and grinding systems. All the samples were embedded in EXAKT Technovit 7200 one-component resin and then cut to thin sections using an EXAKT300CL automatic cutting system. All these sections were grounded using an EXAKT 400CS variable speed grinding system with P800 and P4000 abrasive papers. The polished thin sections have a thickness of about 60 μm for bone and 30 μm for eggshells. After that, these sections were observed and photographed using a Zeiss Primotech polarized light microscope for general morphology and under a Zeiss MA EVO25 scanning electron microscope for the ultrastructure of eggshells, respectively.

**Computed tomographic scan and 3D reconstruction**. The embryo-containing eggs GZPM VN004-1 and 004-2 were scanned using Phoenix Vtomex M micro-computed tomography Scanner at the Yinghua Inspection and Testing Shanghai Company in Shanghai City. Scanning parameters were set to 200 KV tube voltage and 100 μA current with a voxel size of 22.49 μm^3^. Two additional embryos (GZPM VN006-1; GZPM VN006-2) were scanned using the same instrument at the Key Laboratory of Vertebrate Evolution and Human Origin of Chinese Academy of Sciences, IVPP (energy setting of 180-200 kV and 140-150 Μa with a voxel size of 34.573 and 35.044 µm^3^, respectively; see Supplementary Table 13 for detailed parameters). Then the scanning images were optimized by selecting automatic geometric correction and outputted by tiff format using the software of Phoenix datosx 2.0. Bones were detected clearly in the resulting images (Supplementary Fig. 6a). Reconstruction of radiographs was performed using the software Mimics 17 at the IVPP. A global threshold was manually selected for each specimen to obtain as many bones as possible. Segmentation of all the bones mainly uses the “Region growing” tool, and the bones were drawn manually if they were not distinguished from the surrounding rock by threshold.

**EDS analyses.** In EDS analysis, spectra were obtained with an Oxford X-act detector. A beam current of 700 pA, 30 kV accelerating voltage was used. The working distance was kept between 8-10 mm. Acquisition time was set up to 60 seconds for each EDS spectrum and a count of 10000 to 20000 per second.

**EBSD analysis**. One eggshell thin section was polished with 0.5 μm alumina compound for 30 min, and then with 0.05 alumina compound for 1 h using a MP-1A automated polishing machine to remove the superficial amorphous layer. SEM and EBSD analyses were carried out on a Zeiss Sigma 300VP SEM and an Oxford Instruments Aztec Symmetry EBSD detector at the SEM-EBSD laboratory, School of Earth Sciences, China University of Geosciences (Wuhan). Electron backscatter diffraction patterns (EBSPs) were obtained using 20 kV acceleration voltage working distance of 18.5 mm, 70° sample tilt angle and low-vacuum mode of 20 Pa. The grad spacing used was 0.3 μm. EBSPs were collected and indexed with an automatic mapping mode using the Aztec 6.0 software from Oxford Instruments. All EBSPs of calcite were indexed with the crystal structure parameters from the data in EBSD system: space group RC, a = 4.938Å, c = 116.832 Å [3]. To assure data quality, only those measurements with mean angular deviation (MAD) values below 1° were accepted for analyses.

**Organic δ^13^C analysis**. Sampling for Organic δ^13^C was carried out at the Pingba Section. 29 samples were collected at intervals of about 0.1 m (Supplementary Table 2). Sampling covered a total thickness of about 5 m, and encompassed the fossil beds. We also collected nine samples from fossil beds for comparison. Approximately 2-3 g powdered samples were decarbonated with 10% HCl at room temperature for 24 h in order to completely dissolve the carbonate in the samples. Insoluble residues were washed with deionized water and centrifuged until neutral and then dried overnight in an oven at 60 ℃. The carbonate contents of these samples were calculated by the weights of the powders prior to and after acid treatment.

The organic carbon isotope compositions (δ^13^C_org_) were determined by an elemental analyzer (Flash EA2000) coupled to a ThermoFisher MAT 253 IRMS at State Key Laboratory of Geological Processes and Mineral Resources, China University of Geoscience (Wuhan). Approximately 26-30 mg of samples were accurately weighed and folded into small tin cups that were sequentially dropped into a catalytic combustion furnace (Cr_2_O_3_ and silver Co_3_O_4_ as the fillers, respectively) operating at 1040 ℃. After 3 seconds of flash combustion, O_2_ was resorbed in the reduction furnace and the temperature was fixed at 680 ℃. Water was removed from the combustion products with a magnesium perchlorate column, and the CO_2_ was separated from other gases with a GC column (The oxygen flow was set as 250 mL/min, and the carrier helium flow was set as 90-110 mL/min.). The effluent from the elemental analyzer (EA) was introduced in a flow of He to the isotope ratio mass spectrometer (IRMS) through a ConFio IV to test. Standards GBW04407 (δ^13^C_org_ = –22.4‰) and GBW04408 (δ^13^C_org_ = –36.9‰) were used to calibrate the carbon isotope analyses.

**Carbonate ^13^C and ^18^O analysis.** Carbonate concretions were selected from the sediment (29 samples) and fossil beds (nine samples) and were powdered for carbonate ^13^C and ^18^O analyses (Supplementary Table 2). The carbonate carbon and oxygen isotope compositions were analyzed online by a Kiel IV carbonate device coupled to a ThermoFisher MAT 253 isotope ratio mass spectrometer (IRMS) at China University of Geosciences (Wuhan). Approximately 90-120 μg of powdered samples were reacted with 100% H_3_PO_4_ under vacuum at 70 ℃. The produced CO_2_ was collected in trap 1 using liquid nitrogen (-196 ℃). The trap 1 was subsequently heated (-90℃) in order to transfer the CO_2_ into trap 2. Then, trap 2 was heated to 30 ℃ and the CO_2_ was transferred to the MAT 253 IRMS for isotope analysis. All δ^13^C_carb_ and δ^18^O_carb_ values are reported in ‰ relative to the Vienna Pee Dee belemnite (V-PDB) with an accuracy better than ± 0.1‰ and ± 0.2‰ in δ^13^C_carb_ and δ^18^O_carb_ isotope, respectively based on multiple analyses of laboratory standards (GBW04416, δ^13^C_carb_ = +1.61‰, δ^18^O_carb_ = –11.59‰; GBW04417, δ^13^C_carb_ = –6.06‰, δ^18^O_carb_ = –24.12‰; and internal standard, ISTB-1, δ^13^C_carb_ = –10.63‰, δ^18^O_carb_ = –8.63‰).

**Eggshell porosity calculation and nest type inference.** Eggshell porosity relative to egg mass has been demonstrated to be highly correlated to nest types in living species and thus the produced regression models from living species can be used to predict the nest type of extinct species [4]. We inferred the nest type of *Qianlong* using the method and dataset from ref. [4] with *Qianlong* added in (Supplementary Table 5). The average individual pore area and pore density data were collected by using AxioVision SE64 software. Eggshell porosity (Ap * Ls^-1^, in mm) was determined by total pore area of an egg (Ap) and pore length (Ls). The latter is difficult to measure and thus eggshell thickness is used for the pore length. Tangential sections were photographed with microscope to measure the average individual pore area (A). Number of pores was divided by the examined eggshell area to calculate Pore density (D). Total pore area of an egg (Ap) was calculated by multiplying average individual pore area (A), eggshell surface area (As) and Pore density (D). *Qianlong* has a log Ap·Ls^-1^ value of 2.062 and a log M value of 2.746, and is plotted near the regression line of the covered nesters (Supplementary Fig. 13).

**Ancestral state reconstruction (ASR) of selected reproductive characters**

**Taxon sampling.** The sampled taxa for the reconstruction of the ancestral states of egg traits are mainly from four recent studies on this subject [5-8], but are also with addition of 10 taxa that are not covered in these studies (see below for detailed information). The sampled taxa cover major reptilian clades, including crocodilians, birds, non-avialan dinosaurs, pterosaurs, turtles, lepidosaurs, and choristoderes (Supplementary Tables 9, 10), and in total the datasets include 210 taxa. For the extinct taxa, we only sampled taxa that are represented by both skeletal and egg fossils.

Thirty-eight non-avialan dinosaur taxa were sampled in our analyses. Non-avialan dinosaur eggs are in general not able to be associated with a particular theropod taxon, though eggs of several sauropodomorphs such as *Mussaurus* [9] and *Massospondylus* [10], some deinonychosaurs such as *Troodon* [11] and *Deinonychus* [12], and a number of oviraptorosaurs [13, 14] are known based on the associated embryonic or adult skeletons. Some ootaxa such as *Megaloolithus patagonicus* and *Megaloolithus sirugei* are widely accepted to be titanosaurs based on associated embryos, though they are not assignable to a specific low-ranked taxon [15]. Some ootaxa (e.g., some titanosaur and oviraptorosaur egg species) were able to be confirmed at a low taxonomical level based on published data of morphology, age, and geographical distribution (Supplementary Table 14).

Sixty-eight extant birds and seventeen extinct species were sampled in our analyses. Eggs of extinct birds have been identified based on either associated embryonic or adult skeletons, and they include the enantiornithines *Neuquenornis volans* [16], *Gobipteryx minuta* [17], *Gobipipus reshetovi* [18], *Avimaia schweitzerae* [19], some other indeterminate taxa [20-23] as well as a phoenicopterid [24] and *Aepyornis* [25].

Five pterosaur species were included in this study. Pterosaur eggs are rare and only known in *Pterodaustro* [26, 27], *Beipiaopterus* [28], *Yixianopterus* [29, 30], *Kunpengopterus* [31, 32] and *Hamipterus* [33, 34]. Legendre *et al.* [7] include *Darwinopterus* in their analysis, but as the supposed *Darwinopterus* specimen (ZMNH M8802) is actually the counter slab of IVPP V18403 that was identified as *Kunpengopterus* sp. [32], we only keep *Kunpengopterus* for the analysis. In addition, the value of calcareous layer of *Hamipterus* was updated to 0 μm based on ref. [35].

Fourteen crocodylomorph species are included in our datasets for the ASR analyses. Among them, 10 taxa are from Legendre *et al.* [7], two extant crocodiles (i.e., *Paleosuchus palpebrosus* and *Mecistops cataphractus*) from Moore and Varricchio [5], and two extinct crocodylomorphs (i.e., the Late Jurassic *Suchoolithus portucalfensis* and the Late Cretaceous *Pissarrachampsa sera* [36, 37] are newly added.

Testudines sampling includes nineteen taxa from Legendre *et al.* [7] and three new fossil taxa (i.e., *Neixiangoolithus yani*, *Adocus* and *Desmatochelys padillai*). *Neixiangoolithus* *yani* was discovered from the Upper Cretaceous of Henan Province and the embryonic remains suggest it belongs to the extinct group Nanhsiungchelyidae [38]. *Adocus* eggs from the Late Cretaceous of Alberta were found inside the body cavity of a turtle fossil that is closely related to Nanhsiungchelyidae [38]. *Desmatochelys padillai* is a marine turtle discovered from the Early Cretaceous of Colombia, but it lays rigid eggs as in terrestrial turtles[39]. We excluded most turtle egg fossils in Legendre *et al.* [7] dataset because these fossils have uncertain systematic positions within the Testudines. However, we include *Testudoflexoolithus bathonicae* and *Testudoolithus lordhowensis* in our analysis because the systematic positions of these two taxa are better known. The Late Jurassic *Testudoflexoolithus bathonicae* has flexible eggshells as extant marine turtles [40]. The Pleistocene *Testudoolithus lordhowensis* is large and spherical in shape, and with a thick eggshell, and it is associated with *Meiolania platyceps* [41], which is basal to Testudines [42].

Forty-five lepidosaurs are included in our dataset, including forty-two species from Legendre et al. [7], and three newly added taxa. The latter include an Early Cretaceous anguimorph [43] and two varanids (i.e., *Varanus komodoensis* and *Varanus indicus*) that are closely related to this fossil taxon.

For Choristoderes, the only known species associated with eggs is *Hyphalosaurus baitaigouensis* from the Early Cretaceous of China [44].

**Eggshell type scoring**. Legendre *et al.* [7] divide diapsid eggs into three types (i.e., soft, hard, and semi-rigid), and list three different ways to score egg type: the “new scoring” is based on original references; “ratio scoring” is based on the ratio of calcareous layer to the total thickness; and “shell unit scoring” is based on presence/absence of interlocking shell unit. This results in three different datasets for eggshell-type ASR analyses. Here we use the criterion of new scoring and ratio scoring to do ASR analysis for their widely used in eggshell-type definition.

It should be noted that we modified a few scorings in Legendre et al. [7] (Supplementary Table 11). The ratios of the calcareous layer to the whole eggshell thickness of *Masssospondylus* and *Lefengosaurus* egg are greater than 50%, suggesting a leathery eggshell type, and thus we modified these scorings. Legendre et al. [7] scored *Pterodaustro* egg as hard-shelled in all three scoring strategies, though *Pterodaustro* had a very thin calcareous layer (50 μm) and showed no evidence for the presence of eggshell unit. Therefore, it is more reasonable to change the scores to leathery (new scoring) and question mark (ratio scoring), respectively. The Middle Jurassic ootaxon *Testudoflexoolithus bathonicae* is diagnosed by its small size, ellipsoidal shape, relatively thin eggshell thickness (between 0.2 and 0.25 mm) and a shell unit with a height-to-width ratio of 1.0 [40]. The loosely abutting shell units are similar to those of sea turtle soft eggshells. Therefore, the “new scoring” was modified as soft, and the “ratio” scoring was changed to leathery basal on Hirsch [40]. Two extant turtles, *Testudo graeca* and *Testudo marginata* were scored as soft eggshells based on ratio scoring [7]. However, the value of ratio scoring of *T. graeca* was calculated to be 77 percent in recent research [45] and thus we changed the scoring to the hard eggshell.

**Body mass**. Body mass data of extant female crocodilians are directly obtained from Thorbjarnarson [46] and those of extant female birds from refs. [47, 48]. Pterosaur body mass data is derived from the equation M= 0.519b^2.550^ (b represents wingspan) [49] and wingspan length data from various literatures [31, 33, 49-51]. Body mass data of most non-avialan dinosaurs are directly obtained from ref. [52]; those of some other non-avialan dinosaurs including *Qianlong shouhu* are obtained using the equation:

${\text{mass}_{\text{biped}}\text{ = (10}}^{\text{(2.749 * log10}\left( \text{FC *}\text{ 2}^{\text{0.5}} \right) \text{- 1.104)}}\text{) / 1000 }$ [53]

Femoral minimum circumference (FC) is measured from relevant species [54, 55]; Body mass data of most fossil birds are obtained by using the equations from ref. [56] and relevant measures from literatures [19, 57, 58] and those of the palaeognaths *Aepyornis*, *Euryapteryx*, *Dinornis*, *Pachyornis* are from literatures [59, 60]. Body mass of extant birds was taken from Dunning Jr [48].

**Egg volume.** Crocodilian egg volumes are estimated using the equation V=0.524L x D^2^ [5] and published relevant measurements [61, 62]. Egg volumes of pterosaurs and birds are estimated using the equation V=0.51 LB^2^ [63] and published relevant measurements [26, 28, 29, 31, 64]. Egg volumes of spherical turtles and non-avialan dinosaur eggs (e.g., hadrosaur and titanosaur eggs) are estimated using the equation 4/3πR^3^ [65] and published relevant measurements [66-72]; elliptical eggs such as *Mussaurus*, *Qianlong*, *Protoceratops*, allosauroid (e.g., *Lourinhanosaurus*), therizinosauroid, and some turtle eggs are estimated using the equation V=0.524L x D^2^ and published relevant measurements[6, 73-75]; Egg volumes of oviraptosaurs, dromaeosaurids, alvarezsaurids, and fossil birds are estimated using the equation V=0.51L x D^2^. Egg volume of lizards is estimated using the equation from Legendre *et al.* [76].

**Eggshell thickness and eggshell calcite layer thickness estimation**. We use the mineralized calcite layer thickness representing the eggshell thickness in the analyses because membrane data are not available for most fossil eggs. For crocodilians, the calcified eggshell thickness data are obtained from a variety of sources [61, 77-80]. For birds, the references for eggshell thickness come from various resources but always without membranes [81-84]. In order to obtain calcite layer thickness data for 35 selected species of extant birds, from which we can only collect the whole eggshell thickness data [85, 86], we have conducted an analysis of the relationship between the calcite layer and total eggshell thickness in eggs of 22 species of extant birds. This analysis found a strong correlation between the calcite layer thickness and total eggshell thickness (Supplementary Fig. 12), and resulted in the equation: Y=0.8897X (X represents the total eggshell thickness and Y represents calcified eggshell thickness), based on which the calcified eggshell thickness can be estimated from the total eggshell thickness data. Using this equation, we are able to estimate the calcite layer thickness for 35 selected species of extant birds in our eggshell thickness evolution analysis.

**Time-scaled informal supertree assembly.** In order to test the macroevolutionary history of several reproduction-related traits, we assembled an informal supertree manually in Mesquite v3.6.1. Informal supertrees, which can combine different source trees and incorporate a large number of taxa or taxonomic occurrences that have not been included in previous quantitative phylogenetic analyses, have been widely used in paleontological research [6, 7, 87].

The supertree assembled for our ASR analyses covers the major clades of Reptilia, and its topology is based on published trees for various reptilian clades. For the extant taxa, avian topology and calibration of extant birds (n=52) were taken from Legendre *et al.* [7]. However, we added more species at the base of Aves, and have a total number of extant birds of 68 and the final topology and node ages of crown birds were constrained based on Prum et al. [88], Feng et al. [89] and Legendre et al. [7]. For the other extant taxa, we refer to ref. [90] for Crocodylia, ref. [91] for Testudines, and ref. [7] for Lepidosauria. For the fossil taxa, the ornithischian topology is based on McDonald *et al.* [92], the sauropodomorph on Chapelle *et al.* [93] and Mannion *et al.* [94], the non-avialan theropod topology on Foth and Rauhut [95] and Bi *et al.* [54], the pterosaurian topology is based on Longrich *et al.* [96]. The supertree was time-scaled with fossilized-birth-death-model with an empty matrix [97, 98] and a strict clock model, with the node ages of the most recent common ancestors (MRCA) of those extant taxa constrained according to recently published molecular time trees, including refs. [88, 89] for birds, ref. [90] for Crocodylia, ref. [91] for Testudines, ref. [7] for Lepidosauria. The fossil ages were fixed as the mean age of the lower and upper bounds from the corresponding stratigraphic ranges that were collected from previous references and the Paleobiology Database (PBDB). To make the model convergent more easily, we also constrain the age of several nodes which include both fossil and extant taxa, such as Archosauria [99], Crocodylomorpha [99], and Lepidosauria [100]. The root age was fixed to 300.9 Ma according to ref. [7].

We executed two independent MCMC runs with four chains (one cold and three hot) per run for 200,000,000 iterations and sampled every 20,000 iterations. The first 10% samples were discarded as burn-in for each run. Convergence was diagnosed with Tracer 1.7 (effective sample size [ESS] > 100 for every parameter) before and after combining the samples from independent runs. The two runs were convergent separately, but achieved a sick convergency when they were combined, so the majority rule consensus tree was summarized for each run.

To address the potential temporal uncertainty problem, we used different ways to date the tree. In this case, only the node ages of the most recent common ancestors (MRCA) of those extant taxa were constrained according to molecular time trees. We executed two independent MCMC runs with four chains per run for 100,000,000 iterations and sampled every 10,000 iterations, and we sampled 20 trees randomly from the posterior distribution combined from the two runs. The total 22 time-scaled supertrees (2 majority rule consensus trees of each run in the first dating analyses, and 20 random posterior trees sampled from the second dating analyses) were used in substantial ASR analyses. The two majority rule consensus trees are shown in Supplementary Figs. 16-17. All tree nexus files are available in online supplementary materials (<https://figshare.com/s/14374b47d33d96aef963>).

**Ancestral State Reconstruction (ASR)**. The sampled taxa cover major reptilian clades, including crocodilians, birds, non-avialan dinosaurs, pterosaurs, turtles, lepidosaurs, and choristoderes (Supplementary Tables 9, 10), and in total the datasets include 210 taxa. Here we use two criteria (new scoring and ratio scoring) to do ASR analysis for their widely used in eggshell type definition [7]. In order to test the macroevolutionary history of several reproduction-related traits, we assembled an informal supertree manually in Mesquite v3.6.1. Informal supertrees, which can combine different source trees and incorporate a large number of taxa or taxonomic occurrences that have not been included in previous quantitative phylogenetic analyses. We used hidden Markov chain model that considers rate heterogeneity and performed ASR analyses of eggshell type under a Hierarchal Bayesian framework in RevBayes.1.1.1 using all rate different model (ARD) under 2 hidden rate classes. We executed two independent MCMC runs for 10,000 iterations and sampled every 10 iterations. Convergence was diagnosed with Tracer 1.7 (effective sample size [ESS] > 100 for every parameter) before and after combining the samples from independent runs (all analyses were converged). All output results and Revbayes scripts for performing analyses are given in Supplementary Figs. 26-32 and online supplementary materials (<https://figshare.com/s/14374b47d33d96aef963>).

Relative egg size and relative eggshell thickness were determined with phylogenetic linear regression with Log10 transformed data. We chose body mass as the size measurement for indexing the relative egg size, and egg volume as the size measurement for indexing the relative thickness of the calcite eggshell. Residuals taken from the regression models were used to indicate the relative egg size and relative eggshell thickness. The phylogenetic linear regression analyses were performed in R 4.1.3 with package “caper”, and Pagel’s Lambda was used to consider the phylogenetic signal (Supplementary Figs. 16-17). Then we performed ASR of both relative egg size and relative shell thickness with supertrees rescaled by Pagel’s Lambda by using function “fastAnc” in package “phytools”, which could correct the non-brownian motion trait evolution. Analyses were repeated with all 22 time-scaled trees. Identically, we performed ASR on log 10 transformed egg elongation index (Ratio of egg long axis to short axis) and eggshell unit index (Ratio of eggshell unit depth to width) with supertrees rescaled by Pagel’s Lambda by using the function “fastAnc”. Analyses were repeated on the two majority rule consensus trees. All ASR results were given in supplementary Figs. 18-25 and online supplementary materials (<https://figshare.com/s/14374b47d33d96aef963>). For details about dataset assembly, see “Ancestral state reconstruction of selected reproductive characters” in supplementary information.

## Discovery, excavation and preparation of *Qianlong* fossils

Pingba dinosaur site, which has produced *Qianlong* fossils, is located at Zhuanpo, Pingba district, about 50 km southwest of Guiyang City (Supplementary Fig. 1). The first *Qianlong* fossils were discovered in 1999 when local brickmakers were collecting red clay from a small hill at Zhuanpo, Pingba district. Subsequently, a team led by two authors of this study (i.e., Huiyang Cai of Guizhou Provincial Museum and Xinjin Wang of Guizhou Provincial Institute of Cultural Relics and Archaeology) conducted an excavation at this site. The excavation started on 10^th^ August and ended on 12^th^ December, and it led to the collection of the fossils described in this study (Supplementary Fig. 2).

Because numerous additional fossils had yet to be collected from this site, a plan was made to continue the excavation in 2000. Meanwhile, Huiyang Cai and Xinjin Wang contacted Xing Xu of the Institute of Vertebrate Paleontology and Paleoanthropology (IVPP), Beijing, and invited him to join the excavation. Unfortunately, the second excavation was given up because most fossil-bearing strata had been removed for collecting red clay to make bricks by the locals before the team started the next excavation. Nevertheless, Huiyang Cai and Xing Xu planned a joint research project on the fossils collected from the first excavation. Because no agreement was reached on how to prepare the fossils, the research work was delayed to 2016. In that year, Rong Zeng of the Guizhou Provincial Museum invited Chun Li of the IVPP to help with the preparation of the fossils and Fenglu Han of China University of Geosciences (Wuhan), Xing Xu, and others to start a research project on the fossils, which resulted in this paper and some other manuscripts currently in preparation that will provide full descriptions and analyses of all collected fossils.

## Geological setting, sedimentology and taphonomy

The Jurassic stratum in Pingba district has a banding distribution along the northeast-southwest direction with an area of about 2.4 square kilometers. The sedimentary red beds are referred to as the Lower Jurassic Zhenzhuzhong Member of the Ziliujing Formation. Ziliujing Formation is subdivided into five members (in ascending order): Qijiang, Zhenzhuzhong, Dongyuemiao, Maanshan, and Da’anzhai members (Supplementary Fig. 1b, c) [101, 102], and lies disconformably on the Upper Triassic Erqiao Formation. The fossil site outcrops the Zhenzhuchong Member (or Zhenzhuchong Formation in some literatures) is a combination of floodplain and lacustrine facies, which is dominated by dark purple claystone, silty claystone and calcareous claystone, containing small calcareous nodules, with interbedded thin layers of silt-fine quartz sandstone [103]. Zhenzhuchong Member is in conformable contact with the underlying Qijiang Member, and has produced some fossils of early-diverging sauropodomorphs in Weiyuan (Sichuan Province) and Dafang (Guizhou Province) [104]. Palynological analysis from the adjacent Sichuan Basin suggests that the Zhenzhuchong member is Early Jurassic in geological age (possibly in Sinemurian) [104, 105], and plant fossils preferred age of Sinemurian to Pliensbachian [106]. Re-Os isotope analysis suggests an age of 180.3±3.2 Ma for Da’anzhai Member of the Sichuan Basin [107].

When the excavation team collected the *Qianlong* fossils at Pingba dinosaur site in 1999, they made a detailed quarry map with grid lines and collected stratigraphical, sedimentological, and taphonomical data, but some original data including the quarry maps were lost in early 2010s. In the summer field season of 2022, we measured two new sections in this dinosaur site (Supplementary Figs. 1d, 10), and analyzed both organic carbon of paleosols and inorganic carbon and oxygen isotopes of calcareous nodules for stratigraphic comparison (Supplementary Figs. 10-11). Based on the remaining data including photographs and notes taken during the 1999 excavation and new data collected in 2022 field season, we made a preliminary sedimentological and toponymical analysis of the *Qianlong* fossil quarry.

At least five clutches of embryo-containing eggs are preserved in a small area of about 15 m^2^; at least three adult/subadult skeletons are preserved at a distance to the egg clutches ranging from 1 to 3 meters; some additional fossils were collected in close proximity, but which are currently not accessible to us for study (most of them are housed at the Pingba Institute of Cultural Relic Administration and some at GZPM but remain to be prepared out). All *Qianlong* fossils were recovered from the lower portion of a 2-m-thick succession of silty mudstone and siltstone in the middle part of the Zhenzhuchong Member, and more specifically, from two distinct horizons comprising silty mudstones. The fossil-bearing beds are positioned about 0.5 m and 1.5 m above the moderate-thickened sandstone layer. The dip angle of the fossil-bearing bed is about 15° measuring the layer of sandstone below the fossil-bearing bed. The upper fossil-bearing horizon is formed by reddish siltstone, and it produces the postcranial skeleton GZPM VN002 (yellow color on bone surfaces). All fossils except GZPM VN002 were collected from the lower horizon which is formed by dark-purple silty mudstone with a thickness of about 0.4 m. They are black in color and are distributed about 0.7 m below GZPM VN002 (Supplementary Fig. 10).

The newly exposed geological sections are located in the north and east regions of the fossil site and have a distance of about 20 m from each other. Section one is about 3.6 meters in thickness, consisting of reddish silty mudstones with a moderated-thick layer of sandstone (20 cm). Section two consists of reddish silty mudstone with a thickness of about 1.6 m. Deep red clay without bedding, Calcium carbonate accumulations, slickensides of the dinosaur fossil beds all suggest paleosol development under arid or semi-arid conditions (Supplementary Fig. 8) [108]. Calcareous nodules are ubiquitous in the whole section and more concentrated in the sandstone layer (more than 90%, Supplementary Fig. 10). Calcareous nodules are usually between 5 mm to 2 cm with a yellow color surface. They are generally sub-globular, ellipsoid, and irregular. Slickensides and mottles are common beneath the sandstone layer but less common above.

There are several taphonomical features consistent with in situ and relatively quick burial and preservation of *Qianlong* fossils. For example, all adult skeletal elements display fresh bone surfaces, without any evidence for a relatively long time of aerial exposure or aerial weathering; nearly all adult skeletal elements are complete and with few breakages; the recovered adult skeletons are in general well-articulated and even small bones such as wrist bones, ankle bones, and phalanges are in their original anatomical positions; the preservation of large bones in hydraulically incompatible muddy deposits also indicates *in situ* remains. GZPM VN001, which preserves the majority of the skeleton, is in nearly perfect articulation, and most interestingly, the skeleton is preserved in a prostrating posture, with the limbs splayed out relative to the axial column. GZPM VN002 and 003, which are incomplete, are also preserved in a prostrating posture, with dorsal ribs symmetrically distributed on either side (Supplementary Fig. 1).

Although there is no definitive evidence of nest construction, the egg clutches are probably preserved in situ. In most cases, eggs are randomly distributed within the clutch (Fig. 2a and Supplementary Fig. 3), but some eggs seem to be in a row (Supplementary Fig. 3e). Most of the eggs and egg clutches are preserved whole within a restricted 20 cm thick silty mudstone. The best evidence for the in situ preservation of egg clutches is derived from the articulation of the embryonic skeletons (Fig.1; Supplementary Fig. 6). For example, two embryonic skeletons revealed by CT imaging are well articulated and preserved a prehatching posture. Even the phalanges and unguals are well articulated and preserved in their original positions. Well-articulated skeletons are extremely rare in dinosaur embryonic fossils [1, 10, 109] because embryonic skeletons are easily disarticulated during burial and preservation. However, the disarticulated skull elements of known embryos and some bones outside the egg suggest that these eggs may be exposed in the air occasionally and disturbed slightly by water flow.

The δ^13^C_org_ values of the studied sections range from -23.08‰ to -21.26‰. The δ^13^C_org_ values are constant around 22‰ in the first 1 m of the section, and then fluctuate to meter level 2.5 m, followed by a constant up to 3.1 m. A 1‰ negative shift is present at meter level 3.2 m, followed by a 1.7‰ positive excursion. In section 2, The δ^13^C_org_ values are constant among 21.5‰, except for a 1.5‰ decrease at the meter level 1 m.

The δ^13^C (VPDB) values of calcareous nodules of all samples range from -11.73 to -7.58 ‰ and δ^18^O values range from -7.30 to -3.79‰ (Supplementary Fig. 10). The δ^13^C values range from -9.65‰ to -7.58‰ at the lower part of section 1 but decrease to -11.73‰ at the meter level 2.2 m (upper layer of the sandstone), and then are constant around 10‰ to meter level 3 m. A positive excursion is seen at the uppermost 0.5 m of section one. The δ^18^O values increase from -6.44 to -3.79‰ of the bottom to the position of 1 meter’s thickness, and then decrease to - 5.13‰ at the meter level 1.6 m (below the layer of sandstone). They have little fluctuation and range from -7.3 to -7.13‰ in the following two meters. A position excursion can be observed in the uppermost 0.5 m. In section 2, The δ^18^O values slightly fluctuate in the first 0.6 m of the section and a positive excursion is shown to the top of section 2. There is a positive trend displayed by δ^13^C and δ^18^O values in the upper portion of both sections. The matching of the features displayed by the δ^13^C record is also good. In summary, the upper part of section 1 parallels the isotope values from the lower and middle parts of section two, which is consistent with lithological features.

The δ^13^C (VPDB) values of calcareous nodules of the fossil beds concentrate between -8.04 to -10.53‰, and δ^18^O values range from -5.33 to -6.59‰. All these values are consistent with stratigraphic level. The variations of δ^13^C (VPDB) values are consistent in these two fossil beds but δ^13^C (VPDB) values are distinct. The average of δ^18^O values is significantly lower than those from the upper layer of the fossil bed 2. All the egg clutches and skeletons GZPM VN001-003 from fossil bed 1 have similar δ^18^O values, suggesting the same stratigraphic layer (Supplementary Fig. 11).

Palynological study and chemical weathering indices from adjacent areas suggest a warm (or hot) and dry environmental condition [103, 110] for where *Qianlong* lived. The ubiquitous dark red colour of the silty mudstone suggests oxidation of iron-bearing minerals above the water table and at least periodic drought during this time. The massive fine brown mudstone, abundant small calcium carbonate nodules, along with slickensides, weak color mottling suggest a floodplain deposit of low energy and is characterized by weakly to moderately developed paleosols. This is very similar to the sedimentary environment of other nesting sites of early sauropodomorphs [1, 87, 109]. The nesting site of *Massospondylus* was inferred to be low intensity of repeated flooding events. The bonebed of embryonic remains of *Lufengosaurus* was interpreted as a low-relief floodplain system that was characterized by periodic ponding. The nests of *Mussaurus* were discovered in a sequence of loessic silt, interpreted as windblown dust deposits on the margins of a Playa-type arid zone lake. The sedimentary environment suggests that these sauropodomorphs prefer laying eggs in fine sediments near the margin of rivers or lakes. Therefore, the nesting site of *Qianlong* was supposed to be a floodplain environment. *Qianlong* might have preferred nesting in these sparsely vegetated silts. Egg clutches could be submerged and buried quickly during flooding, and adult individuals may be suffocated beneath muddy puddles contemporary.

## Additional morphological description

A detailed description of all these materials will be provided in another paper. Here, we mainly focus on the important features of *Qianlong* and comparison with other early-diverging sauropodomorphs.

The preserved skull is as elongated as in other early-diverging sauropodomorphs (Fig. 1). The external naris is as large as in most early-diverging sauropodomorphs [111]. In lateral view, the ventral margin of the premaxillary is horizontal and at the same level as that of the maxilla, but the former is lower than the latter in *Massospondylus* and *Plateosaurus* [112, 113]. A shallow concavity is present at the base of the premaxilla nasal process, which is only seen in *Jingshanosaurus* and *Melanorosaurus* [114]. The posterodorsal narial ramus extends more dorsally than posteriorly and forms an angle of about 60° to the main shaft. The angle is similar to that of *Jingshanosaurus* [114] and *Massospondylus* [112], apparently larger than that of *Yunnanosaurus* (45°) [115], and *Plateosaurus erlenbergiensis* (35) [113]. The lateral surface of the maxillary is convex dorsoventrally along the entire main body, and no maxillary ridge, unlike the condition of *Lufengosaurus* [116]. Seven nutritive foramina are detected on both the paired maxillae, whereas they are absent in *Yunnanosaurus* [115]. The ventral ramus of the nasal was small, short and disarticulated. It is restricted to the dorsal region of the antorbital fossa, as seen in *Lufengosaurus* [116] and *Mussaurus patagonicus* [117]. The preserved maxillary process of the jugal is shallow and slender, and tapers anteriorly forming a sharp process, as in most early-divering sauropodomorphs, whereas it is deep and blunt in *Jingshanosaurus* [114].

The paired mandibles are completely well preserved except missing the distal part of the left one (Fig. 1). It is elongated with a short retroarticular process. The anterior tip of the dentary is slightly down-turned. The external mandibular fenestra is present and approximately 8% of the mandible length, which is smaller than most early-diverging sauropodomorphs (10-15%) [111], but is similar to *Riojasaurus* (7%) [115], larger than *Jingshanosaurus* (5%) [118, 119]. The coronoid eminence is well-developed and about twice the height of the tooth-bearing dentary. The jaw articulation is well offset ventrally, as in most early-diverging sauropodomorphs, but differs from the higher jaw joint with the same level of tooth-bearing present in *Yunnanosaurus*, *Mossospondylus*, and *Thecodontosaurus* [115]. The angular and surangular are relatively short and do not extend anteriorly beyond the external mandibular fenestra, in contrast to *Xingxiusaurus* [34]. The dentary is elongated, approximately 60% the length of the entire mandible. It is shallow and gradually increases in depth towards the posterior end. The surangular is a long, strap-like bone contributing to the dorsal and posterior margin of the external mandibular fenestra. Angular is strap-like and relatively small compared to the size of the surangular.

The holotype has four premaxillary teeth and 17 maxillary teeth based on the well-preserved right side of the skull (Fig. 2). Four premaxillary teeth are also seen in *Massospondylus* [112], *Xixiposaurus* [119] and *Jingshanosaurus*[118], whereas five premaxillary teeth are present in *Plateosaurus* [113] and *Aardonyx* [120]. The number of maxillary teeth is larger than that of *Yunnanosaurus* (less than 16) [121] and *Jingshanosaurus* [118], but is less than that of *Chuxiongsaurus* (22) [122].

The crowns of the teeth of the premaxilla and maxillae seem to be identical. They are all labiolingually compressed, dorsoventrally elongated, and taper apically and slightly curved posteriorly. This is unlike the strongly recurved premaxillary and anterior maxillary teeth in *Jingshanosaurus* [118]. The crowns are slightly expanded mesiodistally but do not overlap each other. The labial surface is strongly convex both anteroposteriorly and dorsoventrally. The tooth enamel on the labial surface bears several longitudinal striations, as in *Chuxiongsaurus* [122]. The distal margin is prominent, separating from the central part by deep grooves. This feature is also seen in *Yunnanosaurus*, but the latter is very weak. The marginal serrations are absent, as in *Yunnanosaurus*, but differ from all other early-diverging sauropodomorphs [115]. No indication of any tooth wear is present.

For postcranial elements, the last cervical and dorsal vertebrae are well-preserved and articulated (Supplementary Fig. 2a). The last cervical vertebra was identified based on the position of the parapophysis entirely located on the centrum. In ventral view, the centrum is sharply keeled. The diapophysis is robust and horizontally oriented. The neural spine is tall, anteroposteriorly narrow, transversely wide and slightly directed posterodorsally. Ten dorsal vertebrates are well preserved and articulated except for missing the neural arched in the posterior four dorsal vertebrae. The first dorsal centrum has a sharp ventral keel as in the last cervical vertebra, but the ventral keel is less sharpened and absent from dorsal 3. The anterior three centra have the same length and height with subsquare outlines in lateral view, but they gradually increase in length towards the posterior dorsal vertebrae, which have subrectangular outlines. Posteriorly, the neural spines become anteroposteriorly long and transversely narrow. The sacrum is composed of at least three vertebrae as in most early-diverging sauropodomorphs (Supplementary Fig. 2d), and four sacrals are usually present in sauropods [123]. The anterior 12 caudal vertebrae are articulated and well-preserved and associated with chevrons Supplementary Fig. 2c). The centra are anteroposteriorly short and tall in anterior caudals, but they progressively increased in length and shortened in height and transversely width towards the posterior caudals.

The scapula is elongated anteroposteriorly, mediolaterally flattened, and strongly arched laterally (Supplementary Fig. 2e). Both the proximal and distal ends are strongly expanded dorsoventrally, and the proximal end is slightly deeper than the distal end as in most early-diverging sauropodomorphs. The humerus is slender and transversely expanded at both the proximal and distal ends with a constricted midshaft (Supplementary Fig. 2g). It connects with the deltopectoral crest with a thick edge as the condition in *Plateosaurus* but unlike the sharp edge in *Mussaurus*, *Massospondylus* and *Adeopapposaurus* [123]. The deltopectoral crest is well developed and projects laterally with a subtriangular outline, as in *Lufengosaurus* and *Yunnanosaurus huangi*, but unlike the subrectangular outline in most other early-diverging sauropodomorphs, such as *Mussaurus* [123]. Metacarpal I is transversely stout but much shorter than Metacarpal II-IV (Supplementary Fig. 2f). It has a proximodistal length of 70% of Metacarpal IV, which is the longest one. Metacarpals II-IV are relatively slender and gradually reduced. The length of metacarpal V is unknown. The complete manual phalangeal formula of *Qianlong* is 2-3-4-1?-?.

The preacetabular process of the ilium is small, triangular in outline, and not beyond the anterior end of the pubic peduncle (Supplementary Fig. 2b), as in other early-diverging sauropodomorphs. The postacetabular process tapers posterodorsally and subtriangular in outline. This is unlike the subsquare-shaped distal end in *Xingxiulong*. The ventral margin of the postacetabular process is nearly straight, as in *Lufengosaurus*, but is unlike the condition in *Yunnanosaurus* and *Xingxiulong* which are strongly concave [34, 124]. The pubic peduncle is long and curved anteroventrally as in all sauropodomorphs. A sharp ridge extends from the ventral margin of the postacetabular process to the posteroventral margin of the ischial peduncle, as in *Yunnanosaurus* [124]. The shafts of the pubes are transversely compressed and expand transversely. Its proximal end projects a thin, deep shaft with a large obturator foramen (Supplementary Fig. 2p). The foramen is partly obscured in anterior view. The ischium consists of a strongly expanded proximal obturator plate and a slender ischial shaft (Supplementary Fig. 2o). The ischial shaft has a subtriangular cross-section, as in other early-diverging sauropodomorphs. The femur is sigmoid in lateral view. The long axis of the femoral head is perpendicular to the shaft of the femur (Supplementary Fig. 2i), as in other early-diverging sauropodomorphs, such as *Mussaurus* [123]. The fourth trochanter extends proximodistally, subtriangular in outline, and is located on the posteromedial region of the proximal half of the shaft, contrasting with the more posteriorly placed fourth trochanter in *Xingxiulong* [34]. The ratio of tibia length to femur length is about 0.81, which is similar to *Yunnanosaurus* (0.85-0.88, ZMNH-M8739) [124], but much larger than *Lufengosaurus*. The fibula is long and slender. The proximal end is anteroposteriorly expanded and transversely compressed. It has a concave medial surface and a convex lateral surface, as in other early-diverging sauropodomorphs, such as *Mussaurus* [123]. All five metatarsals are well preserved (Fig. 1f). Mt I and Mt V are shorter than others. Mt V is the shortest, about half the length of metatarsal III. Mt III is the longest, but only slightly longer than Mt II and Mt IV. The proximal ends of all metatarsals are transversely expanded, but they have different shapes to articulate with each other. The proximal end of Mt V is strongly expanded mediolaterally and four times width than the distal end and a small bulge is present near the mid-part of the lateral margin (Fig. 1f). This feature is not seen in other early-diverging sauropodomorphs and seems to represent an autapomorphy of *Qianlong* here. The distal end of mt V is narrow and trunked, suggesting no phalanx associated to this element, as in *Yunnanosaurus* [124], but unlike the condition in *Jingshanosaurus* and *Gyposaurus sinensis* with well-preserved phalanx V-1 [118, 125]. The pedal digits are well preserved in the right pes. The phalangeal formula is 2-3-4-5-0. The unguals are transversely compressed.

## Amniotic egg type and *Qianlong* eggshell

Extant amniotic eggs are traditionally classified into soft-shelled, flexible-shelled (or leathery) and hard-shelled ones [40, 126]. The soft eggshells are considered to have no or little calcareous layer and when the calcareous layer is present, it is extremely thin and barely recognizable. This type of egg is known in most squamates. The leathery eggshells have a calcareous layer and a thick membrane that can reach 2/3 of the calcareous layer. This type of egg is known in some turtles such as snapping turtles [79]. The rigid eggshells usually have a thick calcareous layer and a thin membrane (e.g., 2:1 to 4:1 in the rigid eggshell of turtles), and the calcareous layer usually consists of well-organized interlocking eggshell units. This type of egg is known in some turtles, some gekkotans, crocodiles, many non-avialan dinosaurs and birds [7, 127].

However, in practice, it is sometimes not easy to categorize eggs, and it is particularly difficult to categorize fossil eggs. For example, the thickness ratio of calcareous layer and membrane is used in identifying soft-shelled or hard-shelled eggs, but there is no widely accepted objective ratio to separate soft, leathery and hard eggshells [6, 79]. Furthermore, this criterion is difficult to apply to fossil eggs because the shell membrane is normally not preserved or not well-preserved in fossil records.

It should be noted that one recent study suggests that the “soft/hard/semi-rigid” classification of reptilian eggshells should be abandoned given a great variety of eggshell morphologies and continuous distribution of several features such as eggshell thickness and calcium content [7]. Nevertheless, mechanically hard-shelled eggs (both rigid and leathery) are clearly different from soft-shelled eggs in having a relatively thick calcareous layer [8]. Both rigid and leathery eggshells display relatively large and sharply edged eggshell fragments when the eggs are broken. The eggshell pieces of leathery eggs such as *Chelydra serpentina* eggs are usually less than 2 mm in maximum diameter, but those of rigid eggs are normally larger than 5 mm in maximum diameter (Supplementary Table 7), and the separation of these two egg types based on eggshell fragment size is supported by our statistical analysis (Fig. 5g).

*Qianlong* eggshell has interlocked eggshell units that are present in non-soft eggs. It has distinct nucleation centers and radially arranged calcite crystals at the inner surface (Supplementary Fig. 14a), and corresponding features occur in *Paraelongatoolithus reticulatus* (IVPP V16514) [128], which is supposed to be *Deinonychus* eggshell [12, 129] (Supplementary Fig. 14b). The right-angled intersection extinction under cross-polarized light in both radial and tangential views also proves the presence of these structures (Supplementary Fig. 14c-d). The IPF X map shows that the c-axes of the calcite grains in the outer portion of the eggshell are vertical to the outer and inner surfaces, but radially surround the nucleation center (Supplementary Fig. 14e), and an IPF X map from a dendroolithid eggshell (*Placoolithus tumiaolingensis*, TML4) [130] shows a similar configuration (Supplementary Fig. 14f). Under partially polarized light, the boundaries of eggshell units are visible in the tangential section through the outer portion of *Qianlong* eggshell. Round and elongated pores are distributed between adjacent eggshell units (Supplementary Fig. 15a). Similar structures can be seen in *Deinonychus* eggshell. Near the inner surface of the eggshell, the eggshell units are separated from each other in both *Qianlong* and *Deinonychus* eggshells (Supplementary Fig. 15c, d). The rugose eggshell surface and strongly pieced eggshells suggest a leathery eggshell type (Fig. 5).

## Supplementary Figures


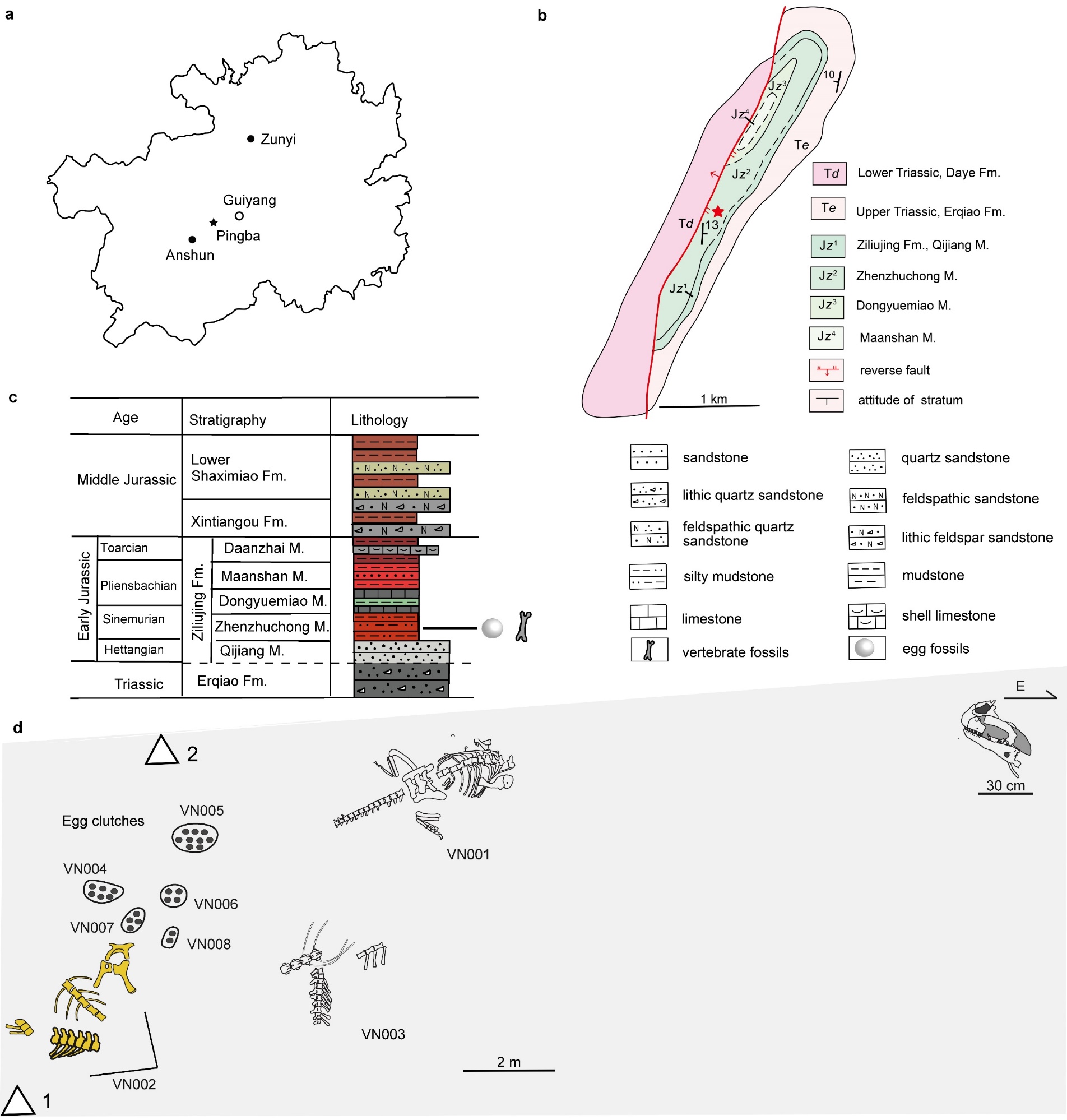


### Supplementary Figure 1. Location, geology and stratigraphy of Pingba dinosaur site and distribution of *Qianlong* fossils within the quarry.

**a**, Map of Guizhou Province showing the fossil site (marked by an asterisk). **b**, General geological map of the fossil site. **c**, Stratigraphic section showing the stratigraphic position of *Qianlong*-fossil-bearing beds. **d**, Distribution of *Qianlong* fossils. GZPM VN002 (yellow color) is from a layer higher than other fossils. Triangles represent the positions of geological sections measured in this study.


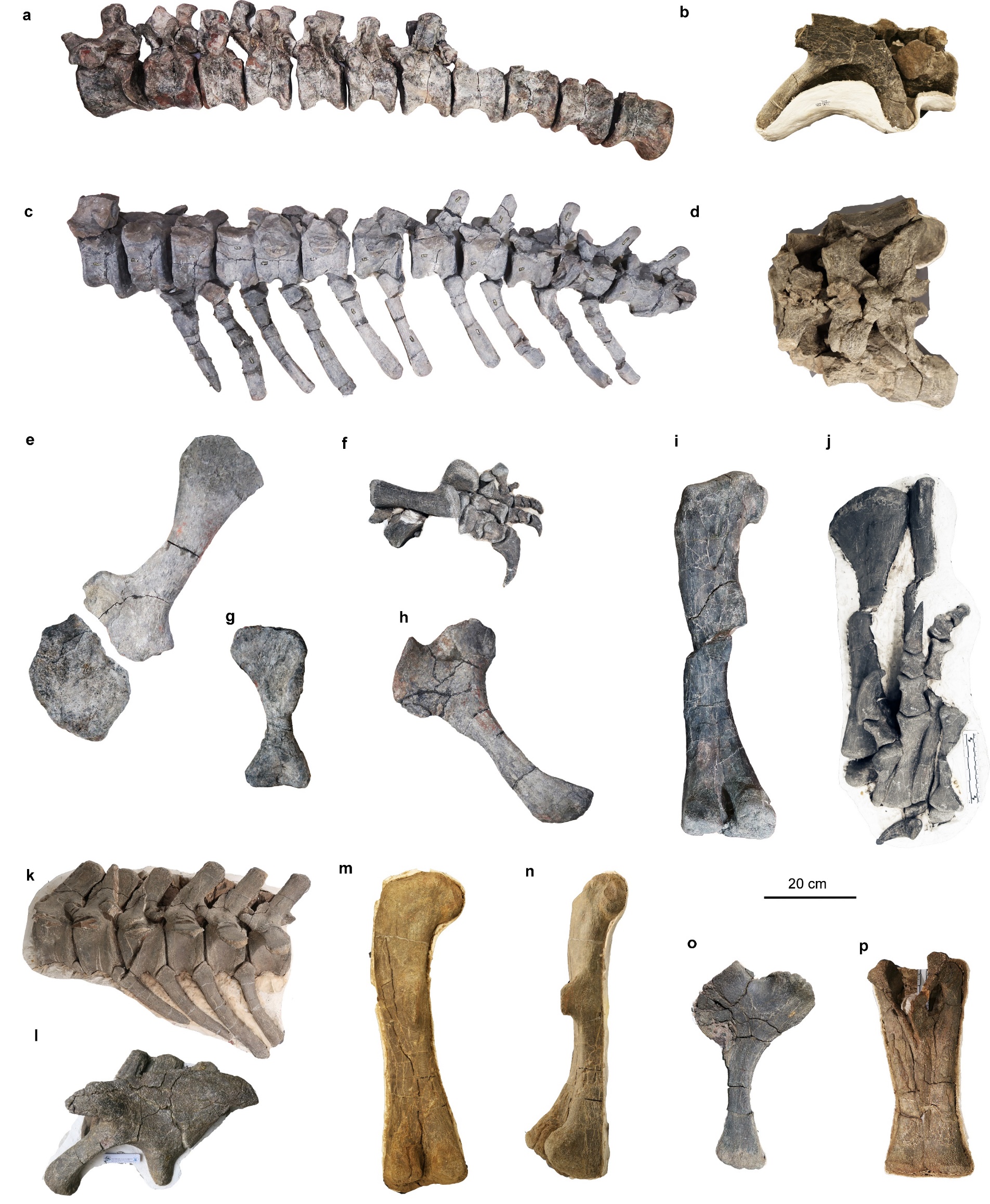


### Supplementary Figure 2. Selected *Qianlong* skeletal elements.

(a-j) GZPM VN001. **a**, Articulated cervical and dorsal vertebrae in lateral view. **b**, Left ilium in lateral view. **c**, Anterior caudal vertebrae associated with chevrons. **d**, Sacral vertebrae associated with ilia in dorsal view. **e**, Left scapula and coracoid in lateral view. **f**, Left ulna, radius and manus in dorsal view. **g**, Left humerus in anterior view. **h**, Left ischium in lateral view; **i**, Left femur in posterior view. **j**, Articulated left tibia, fibula and pes. **(k-p) GZPM VN002.** **k**, Articulated caudal vertebrae with chevrons; **l**, Left ilium in lateral view. **m**, Left femur in posterior view; **n**, Left femur in medial view; **o**, Left ischium in lateral view; **p**, Paired pubes in posterior view.


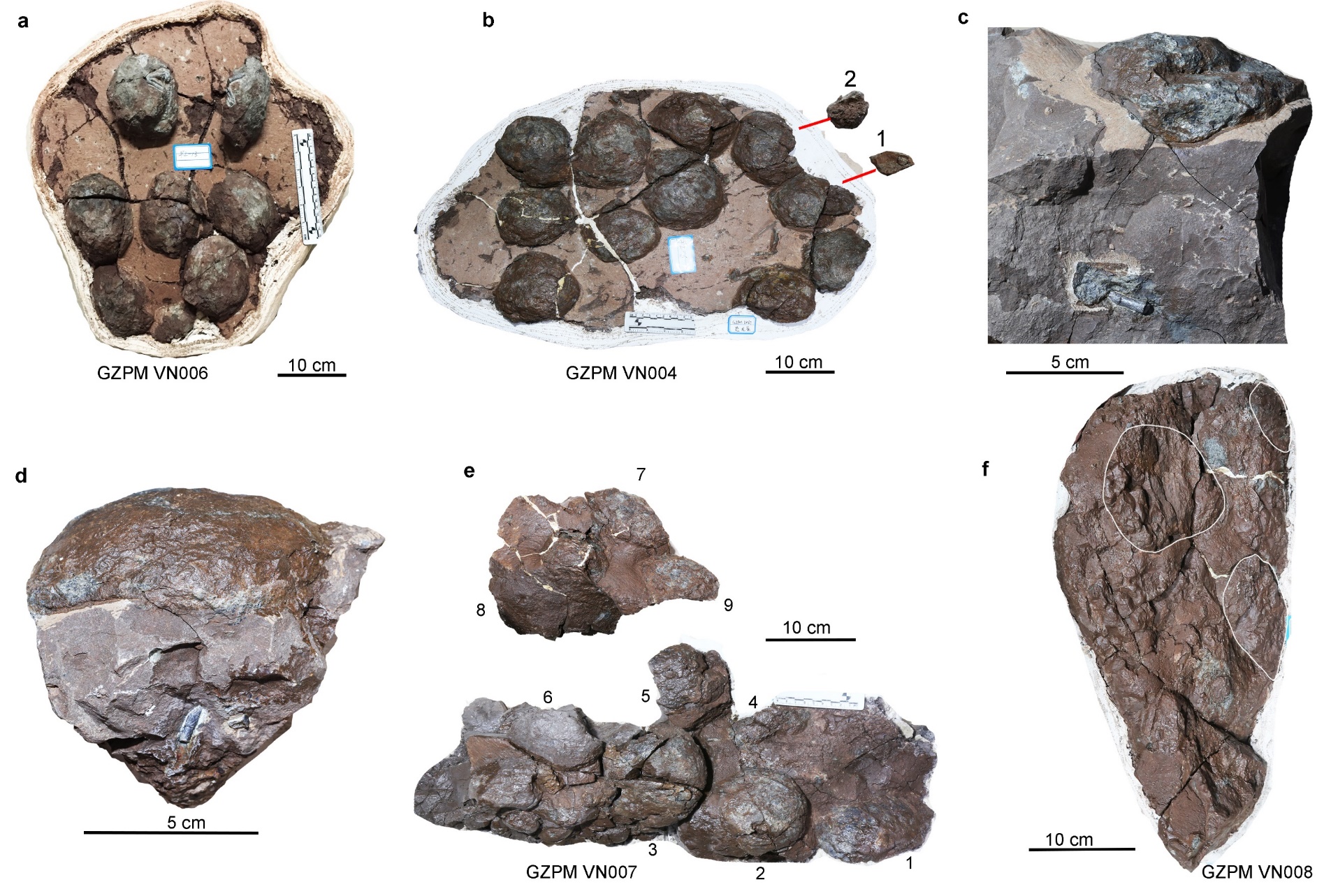


### Supplementary Figure 3. Egg clutches collected from the Pingba dinosaur site.

**a**, GZPM VN006. **b**, GZPM VN004. Numbers 1 and 2 represent the samples taken for histology and CT scans, respectively. **c**, Embryonic remains outside an egg in GZPM VN004. **d**, A complete egg and embryonic remains outside an egg in GZPM VN004. **e**, GZPM VN007 showing nine eggs preserved within two blocks. **f,** GZPM VN008 showing three partial eggs marked by white lines.


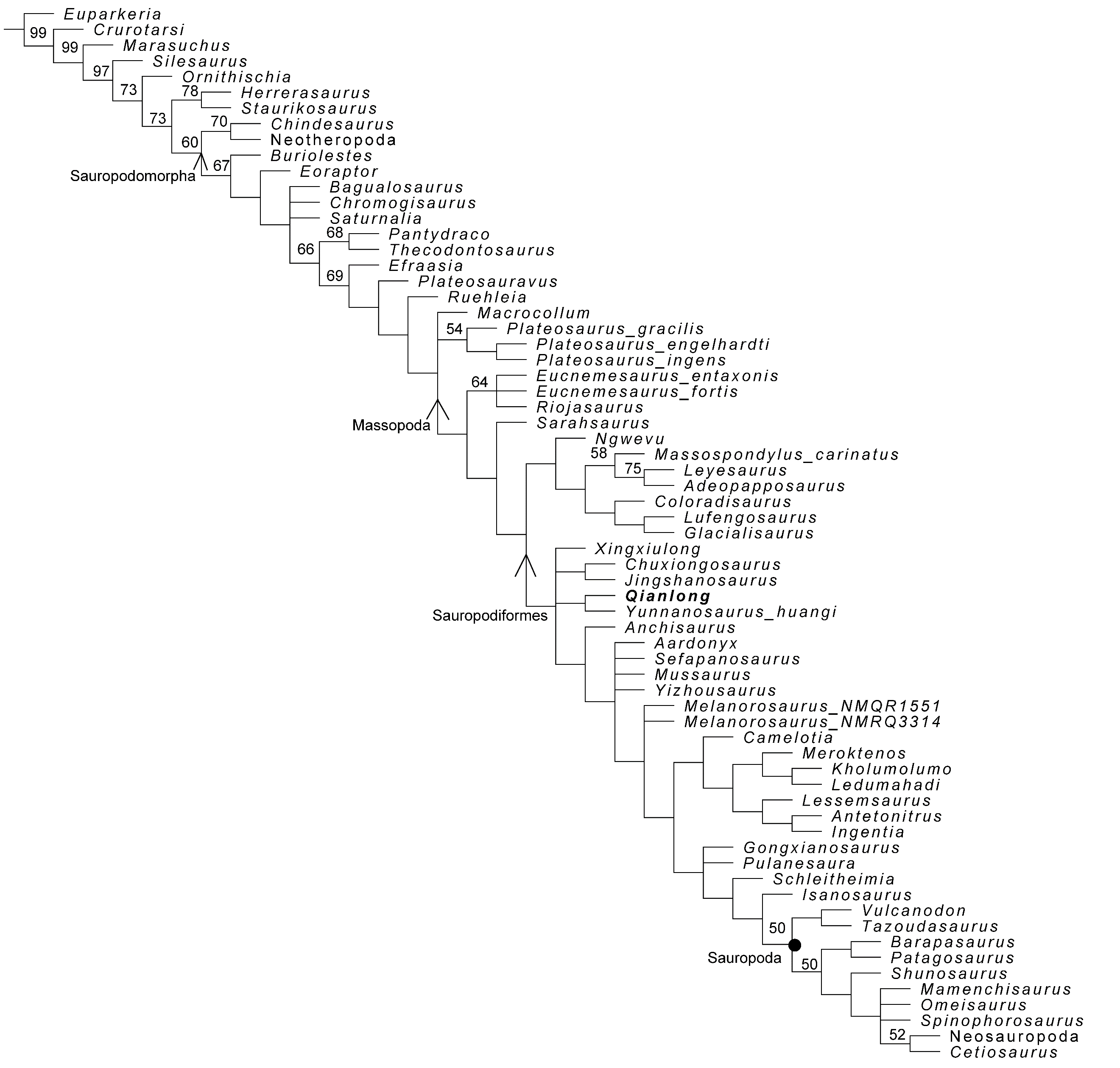


### Supplementary Figure 4. A reduced consensus tree of 2436 most parsimonious trees produced by a parsimonious analysis of a dataset modified from ref. [1] with a posteriori pruning of thirteen unstable taxa

(*Nambalia*, *Jaklapalisaurus*, *Seitaad*, *Leonerasaurus*, *Blikanasaurus*, *Guaibasaurus*, *Irisosaurus*, *Pradhania*, *Xixiposaurus*, *Unaysaurus*, *Agnosphitys*, *Pampadromaeus*, and *Panphagia*). Values above nodes represent bootstrap proportions.


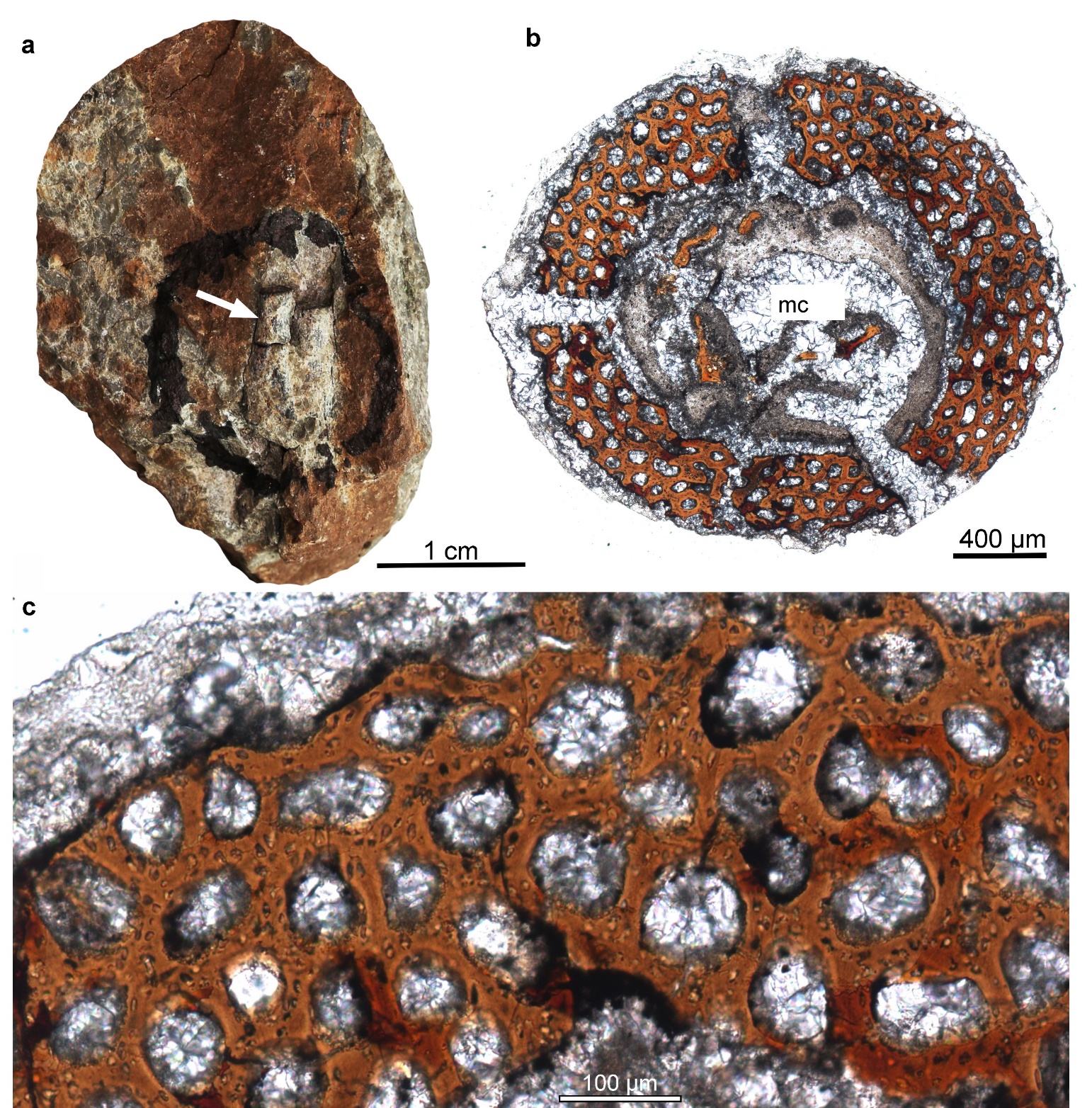


### Supplementary Figure 5. Bone histology of the embryo GZPM VN004.

**a,** Photograph showing histological sample position within the egg. The white arrow indicates the bones (i.e., radius and ulna) that are sampled. **b**, Whole cross-section of the radius at the mid-region of the shaft. **c**, Enlargement of the cortex showing large vascular canals and abundant osteocyte lacunae.


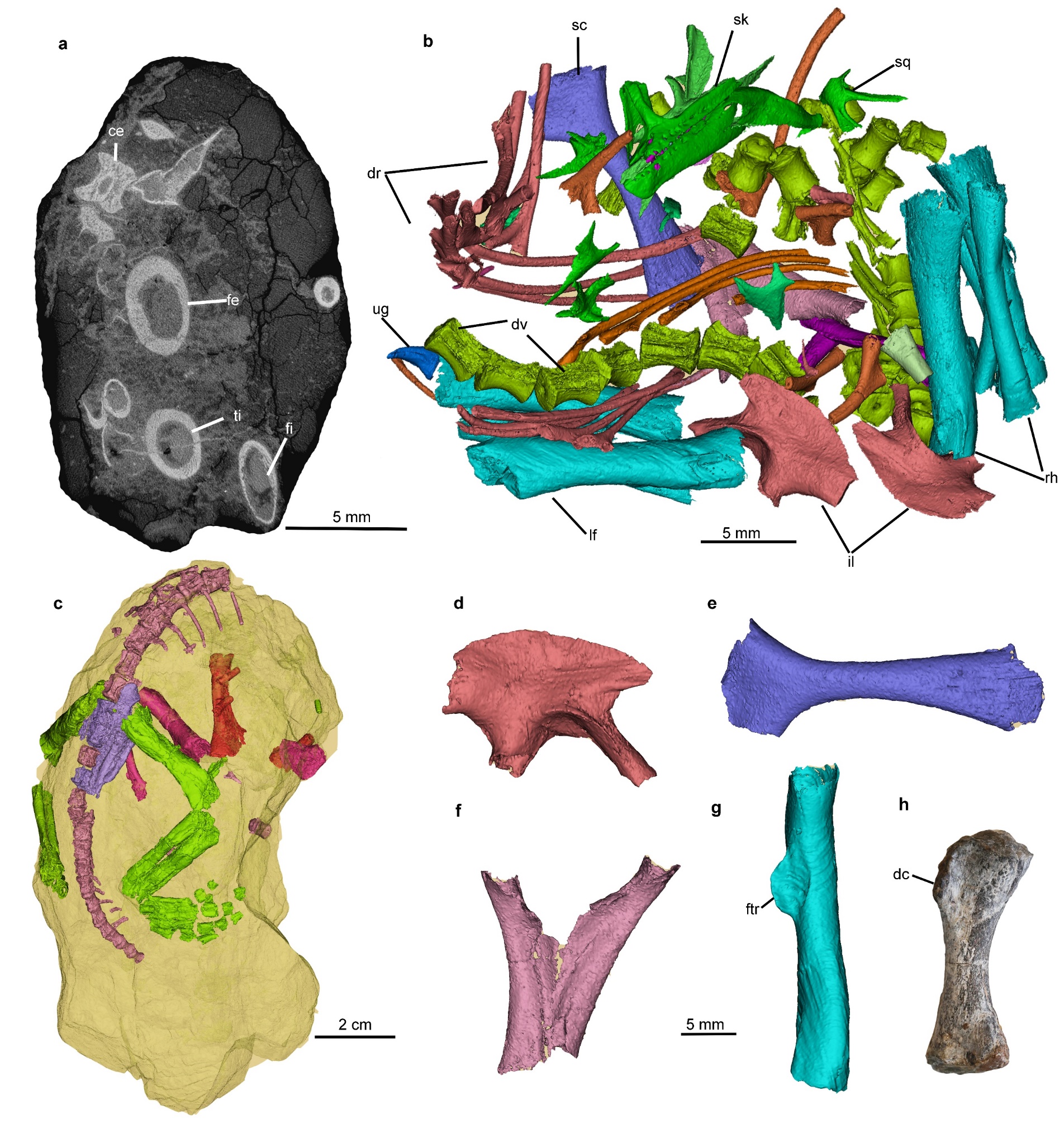


### Supplementary Figure 6. Selected embryonic elements.

CT image (**a**) and 3D Reconstruction (**b**) of GZPM GPZNV004-2. **c,** 3D reconstruction of GZPM VN006-2. (**d-g**) 3D reconstruction of selected elements in GZPM VN004-2. **d,** Right ilium in lateral view; **e**, Left scapula in lateral view; **f,** Right and left pubes in posterior view; **g**, Left femur in lateral view. **h**, Right humerus in anterior view (GZPM VN006-I1). **Abbreviations**: **ce**, centrum; **dc**, deltopectoral crest; **dr**, dorsal rib; **dv**, dorsal vertebrae; **fe**, femur; **fi**, fibula; **ftr**, fourth trochanter; **il**, ilium; **lf**, left femur; **rh**, right handlimb; **sc**, scapula; **sk**, skull; **sq**, squamosal; **ti**, tibia; **ug**, ungual.


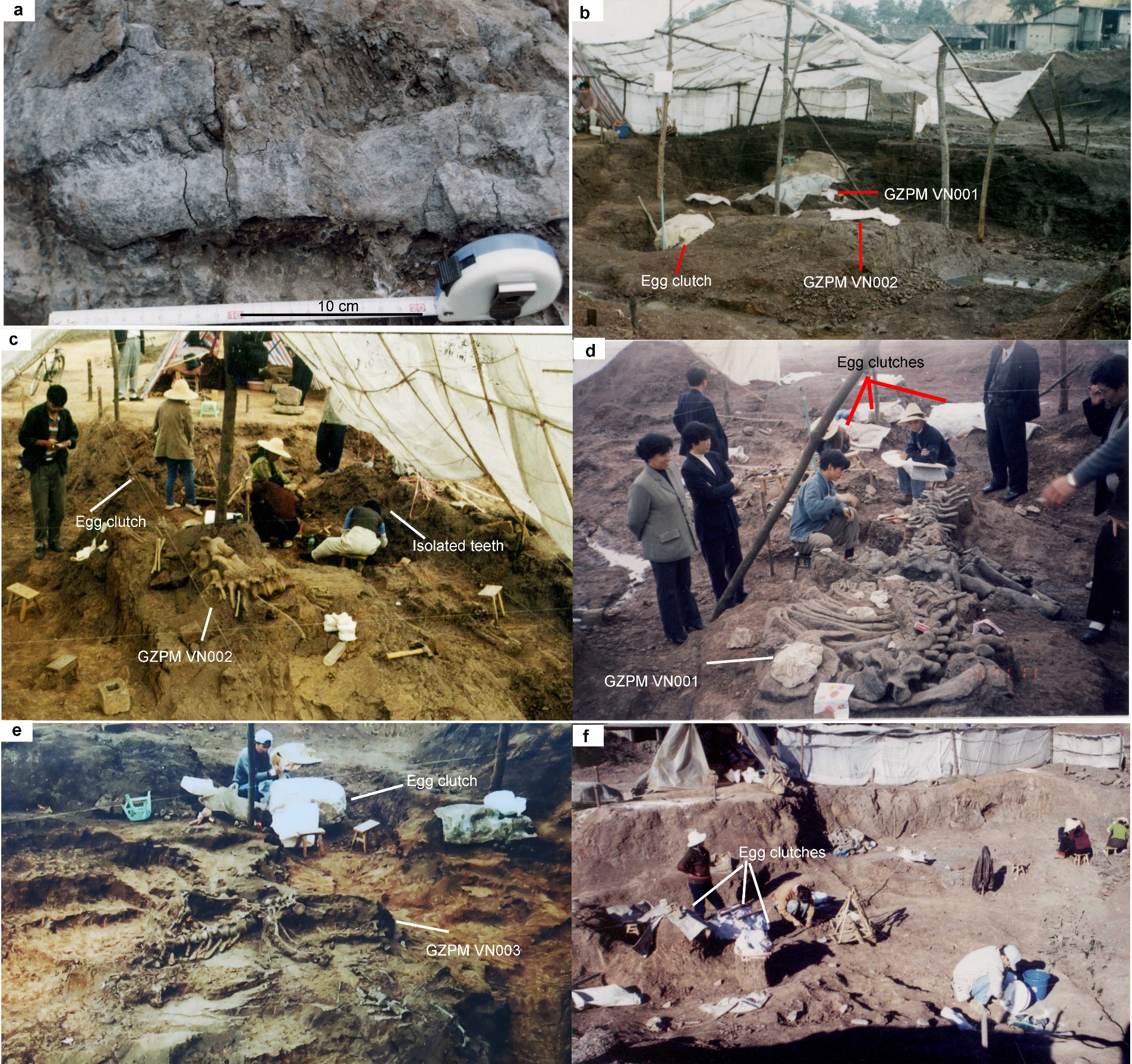


### Supplementary Figure 7. Collecting dinosaur fossils at the Pingba dinosaur site in 1999.

**a,** Partial skull of GZPM VN001 exposed during the 1999 excavation. **b-f**, Photographs of the Pingba dinosaur site taken during the 1999 field season showing the preservation of three articulated skeletons and the egg clutches of *Qianlong* studied in this work.


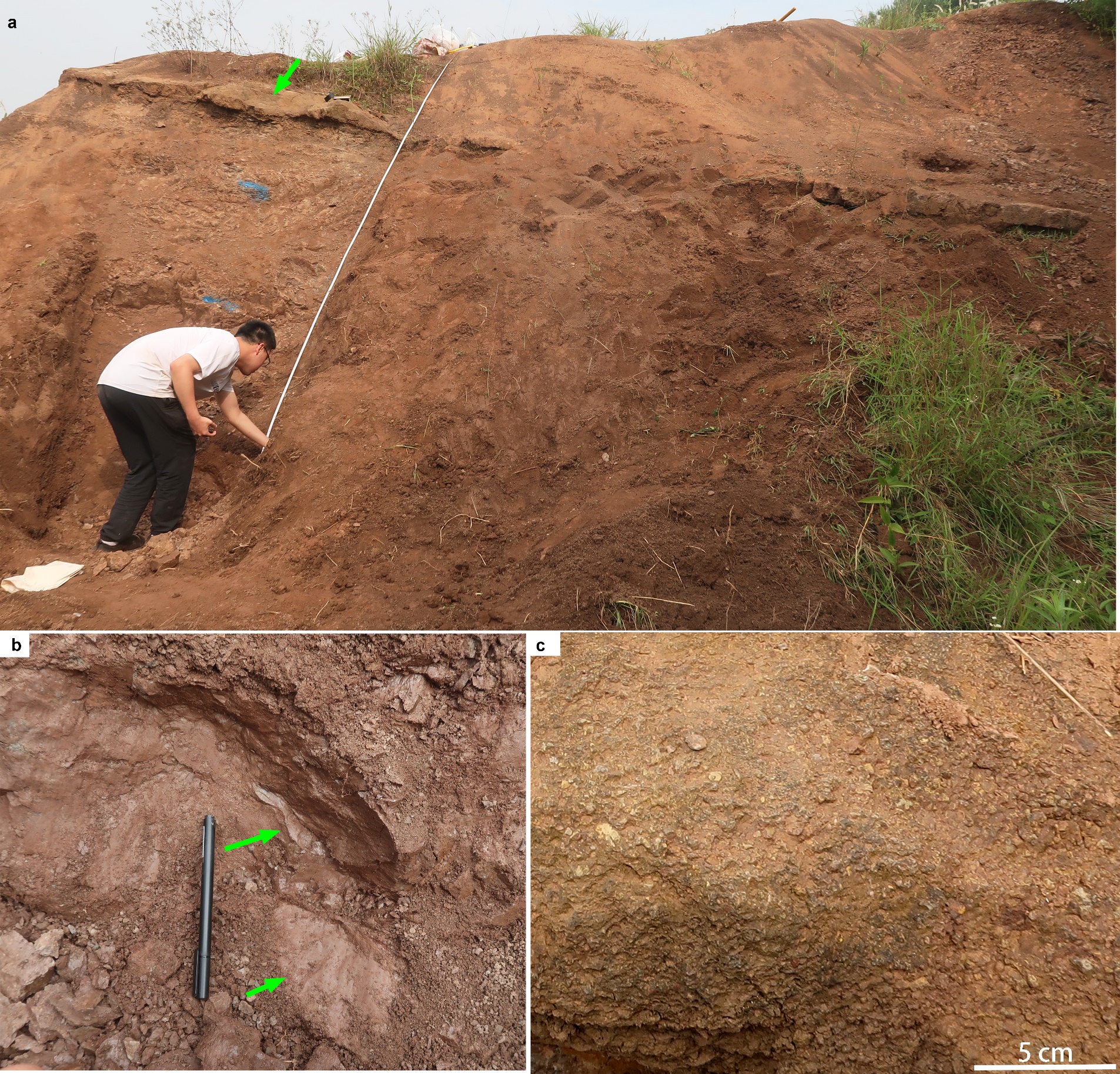


### Supplementary Figure 8. Filed photographs of paleosols from the Lower Jurassic Ziliujing Formation of Pingba Section.

**a**, Thick layer of reddish calcisol interbedded with a medium-thickened layer of carbonate horizon (BK, denoted by arrow). **b**, A typical slickenside in the reddish calcisol, 1 m beneath the concrete horizon. **c**, Dense calcareous nodules in carbonate horizon.


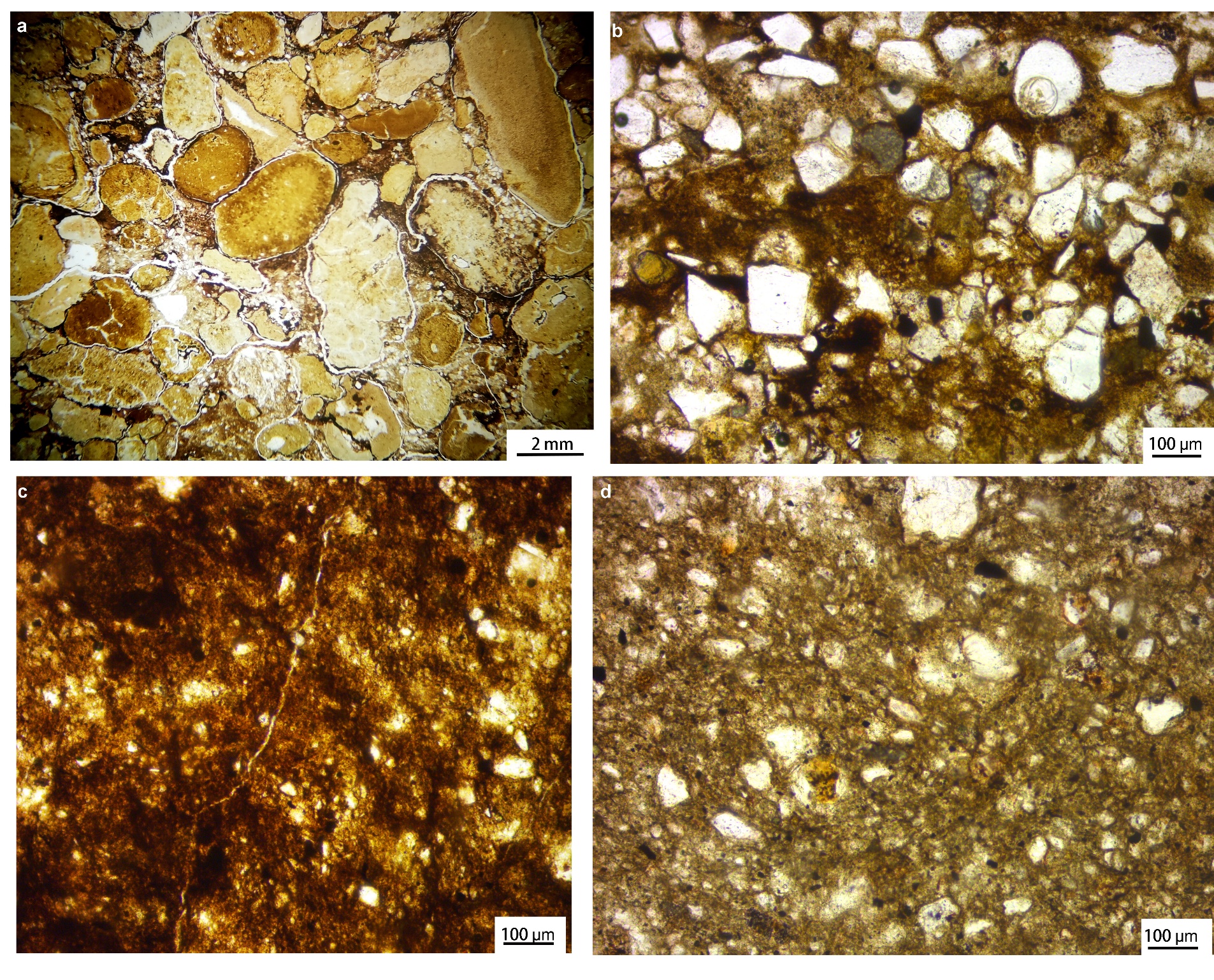


### Supplementary Figure 9. Micromorphological features in paleosols from the Lower Jurassic Ziliujing Formation of Pingba Section.

**a**, Calcareous nodules from BK horizon. **b,** Fine sandstone above BK horizon (bright minerals are quartz). **c**, Reddish silty mudstone from fossil bed 1. **d**, Siltstone from fossil bed 2.


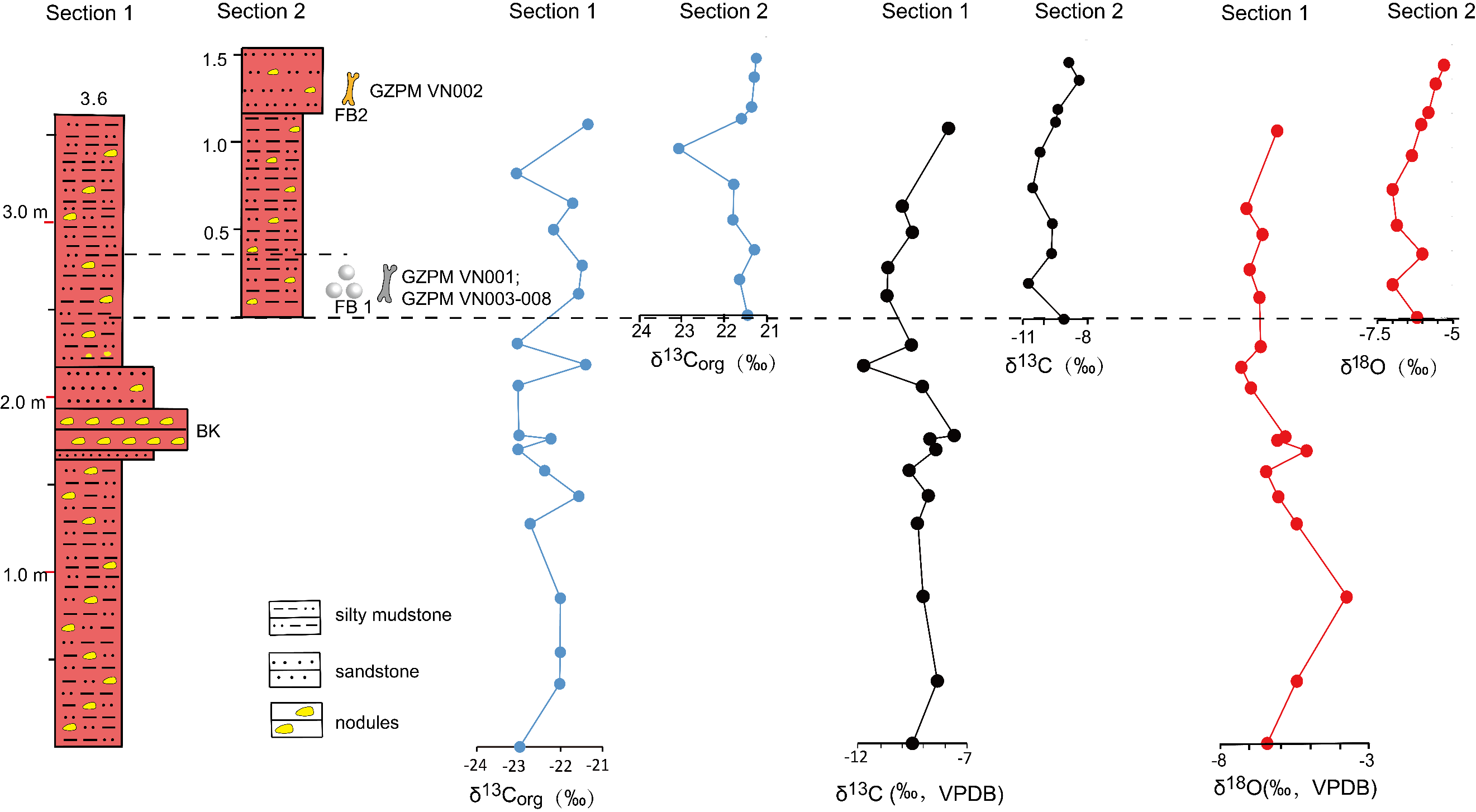


### Supplementary Figure 10. Lithostratigraphic logs and δ^13^C_org_, δ^13^C (VPDB), and δ^18^O values from Pingba sections.


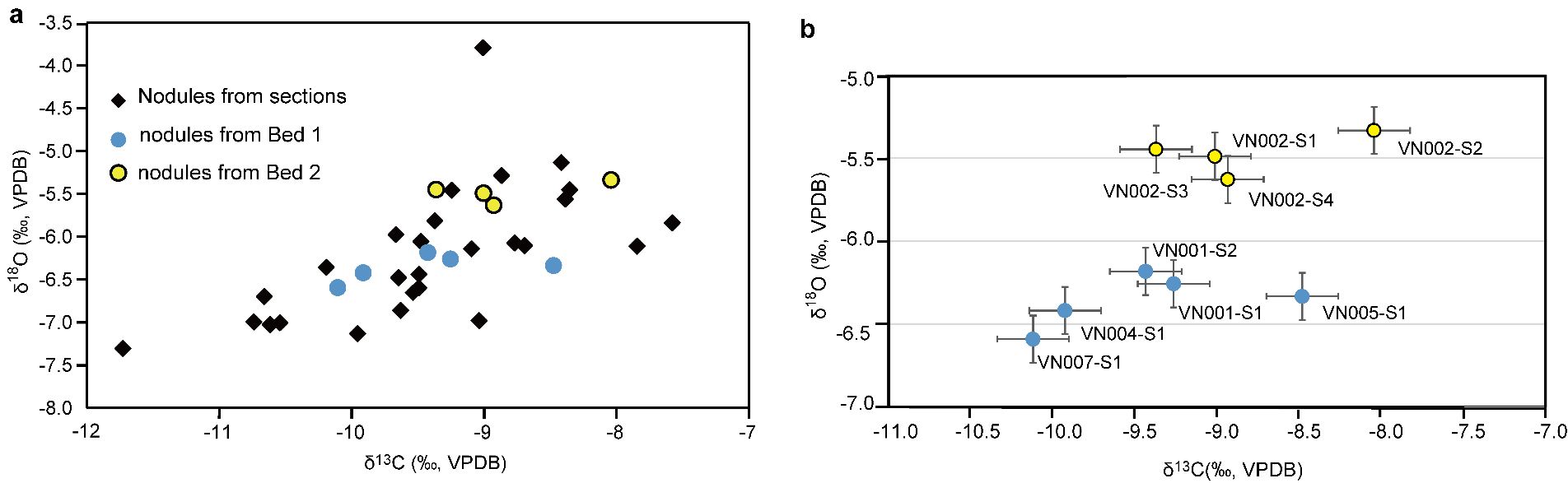


### Supplementary Figure 11. Plots of δ^13^C vs. δ^18^O values in carbonate nodules from Pingba sections (a) and fossil beds (b).

### **Supplementary Figure 12. Bivariate plot of calcified eggshell thickness and total eggshell thickness** in 22 living birds (Supplementary Table 6)

, showing a high and positive correlation between these two values.


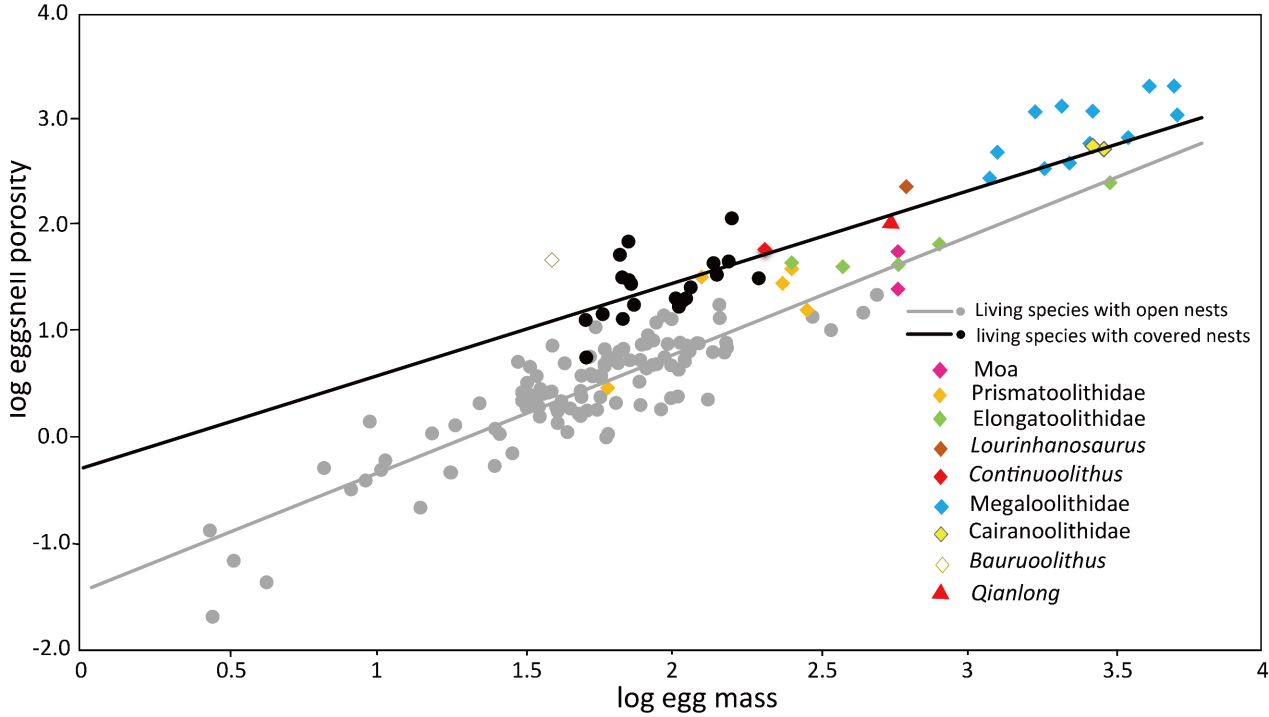


### **Supplementary Figure** 13. Bivariate plot of eggshell porosity and egg mass in living and extinct archosaur taxa/ootaxa.

*Qianlong* shows high eggshell porosity that is suggestive of covered nests.


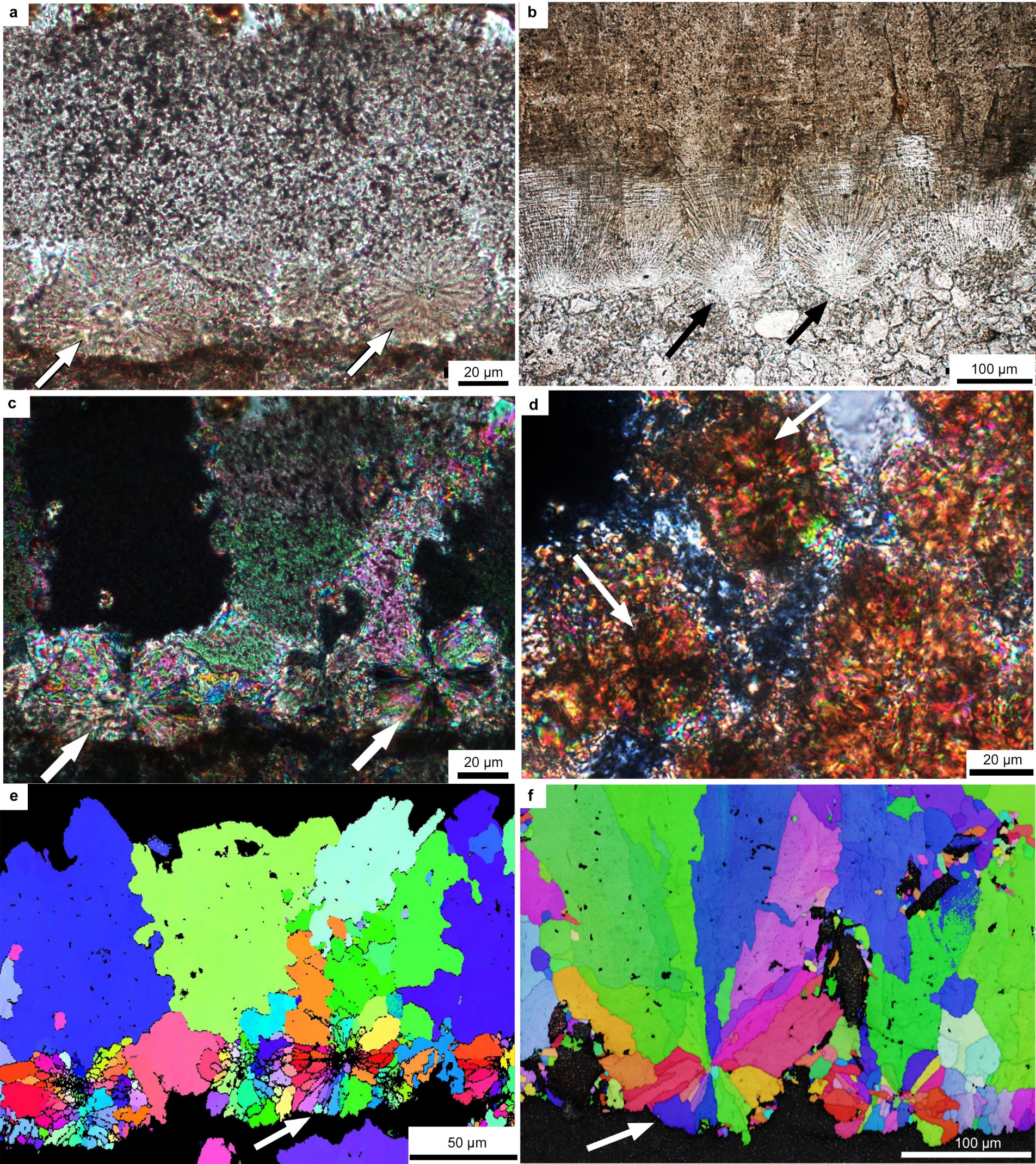


### **Supplementary Figure 14.** Comparison of eggshell microstructure between *Qianlong* and other dinosaur eggs.

Nucleation centers and radial arranged calcite crystals (arrows) at the inner surface of *Qianlong* (**a**) and *Deinonychus* (IVPP V16514) (**b**) eggshells; the right-angled intersection extinction (white arrows) under cross-polarized light in radial (**c**) and tangential (**d**) thin sections of *Qianlong* eggshell; IPF X maps of *Qianlong* (**e**) and *Placoolithus* (**f**) eggshells showing the c-axes of the calcite grains are radially arranged around the nucleation centers (white arrows).


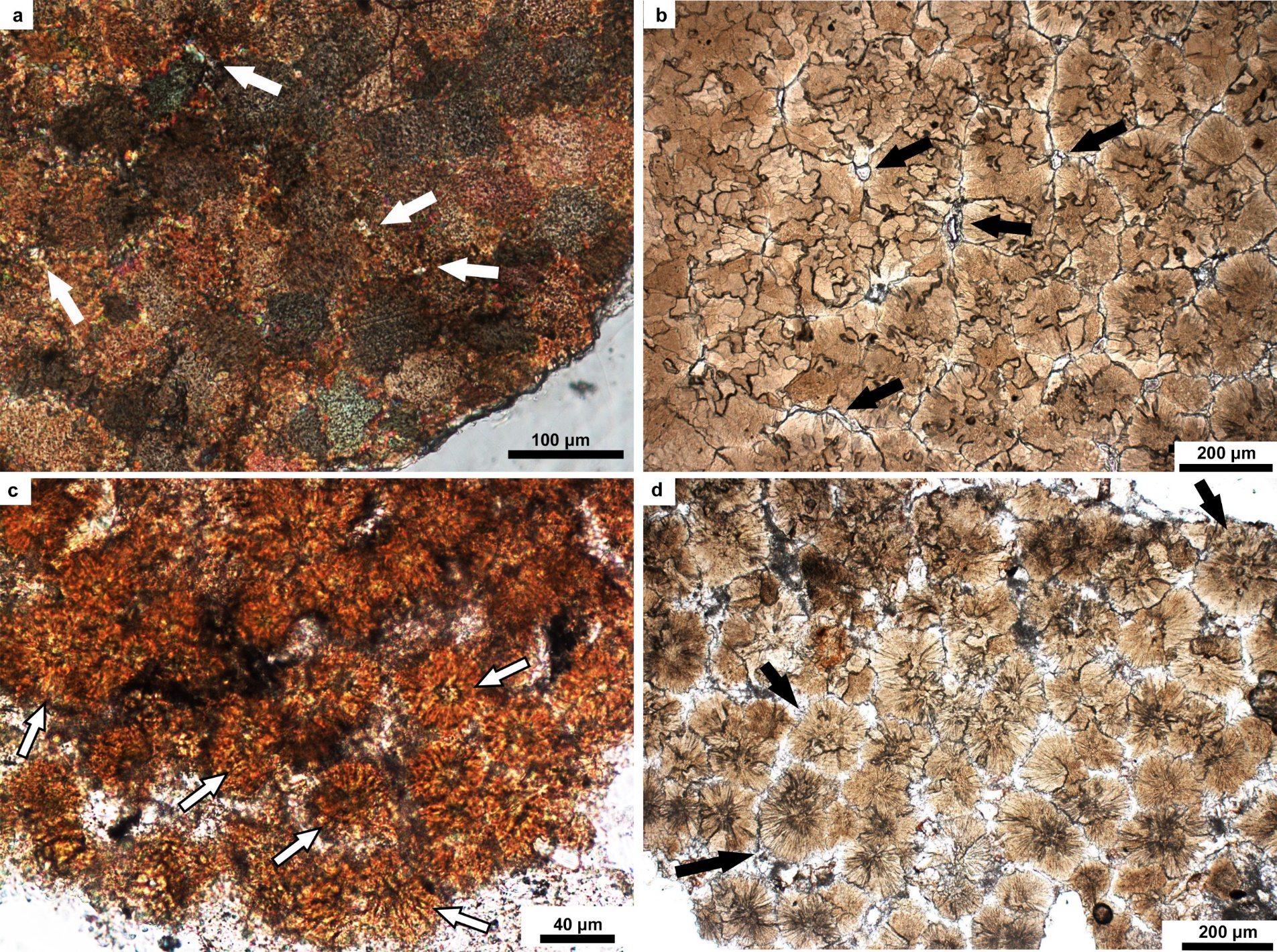


### **Supplementary Figure 15.** Comparison of eggshell microstructure between *Qianlong* and *Deinonychus* through tangential section.

The outer portion of *Qianlong* (**a**) and *Deinonychus* (**b**) eggshells, showing the pores and blocky eggshell units; tangential section through the inner portion of *Qianlong* (**c**) and *Deinonychus* (**d**) eggshells, showing the isolated eggshell units with radial microstructure. Arrows indicate pores in **a** and **b**, eggshell units in **c** and **d**.

### **Supplementary Figure** 16. Time-scaled supertree (majority rule consensus tree of run1 in the first dating analysis) of 210 diapsid taxa used for the character ancestral state reconstruction analysis in this study.

### **Supplementary Figure** 17. Time-scaled supertree (majority rule consensus tree of run2 in the first dating analysis) of 210 diapsid taxa used for the character ancestral state reconstruction analysis in this study.

### **Supplementary Figure** 18. Relative egg size ASR with majority rule consensus tree of run1 in the first dating analysis.

### **Supplementary Figure** 19. Relative egg size ASR with majority rule consensus tree of run2 in the first dating analysis.

### **Supplementary Figure** 20. Egg elongation index ASR with majority rule consensus tree of run1 in the first dating analysis.

### **Supplementary Figure** 21. Egg elongation index ASR with majority rule consensus tree of run2 in the first dating analysis.

### **Supplementary Figure** 22. Relative egg thickness ASR with majority rule consensus tree of run1 in the first dating analysis.

### **Supplementary Figure** 23. Relative egg thickness ASR with majority rule consensus tree of run2 in the first dating analysis.

### **Supplementary Figure** 24. Eggshell unit index ASR with majority rule consensus tree of run1 in the first dating analysis.

### **Supplementary Figure** 25. Eggshell unit index ASR with majority rule consensus tree of run2 in the first dating analysis.


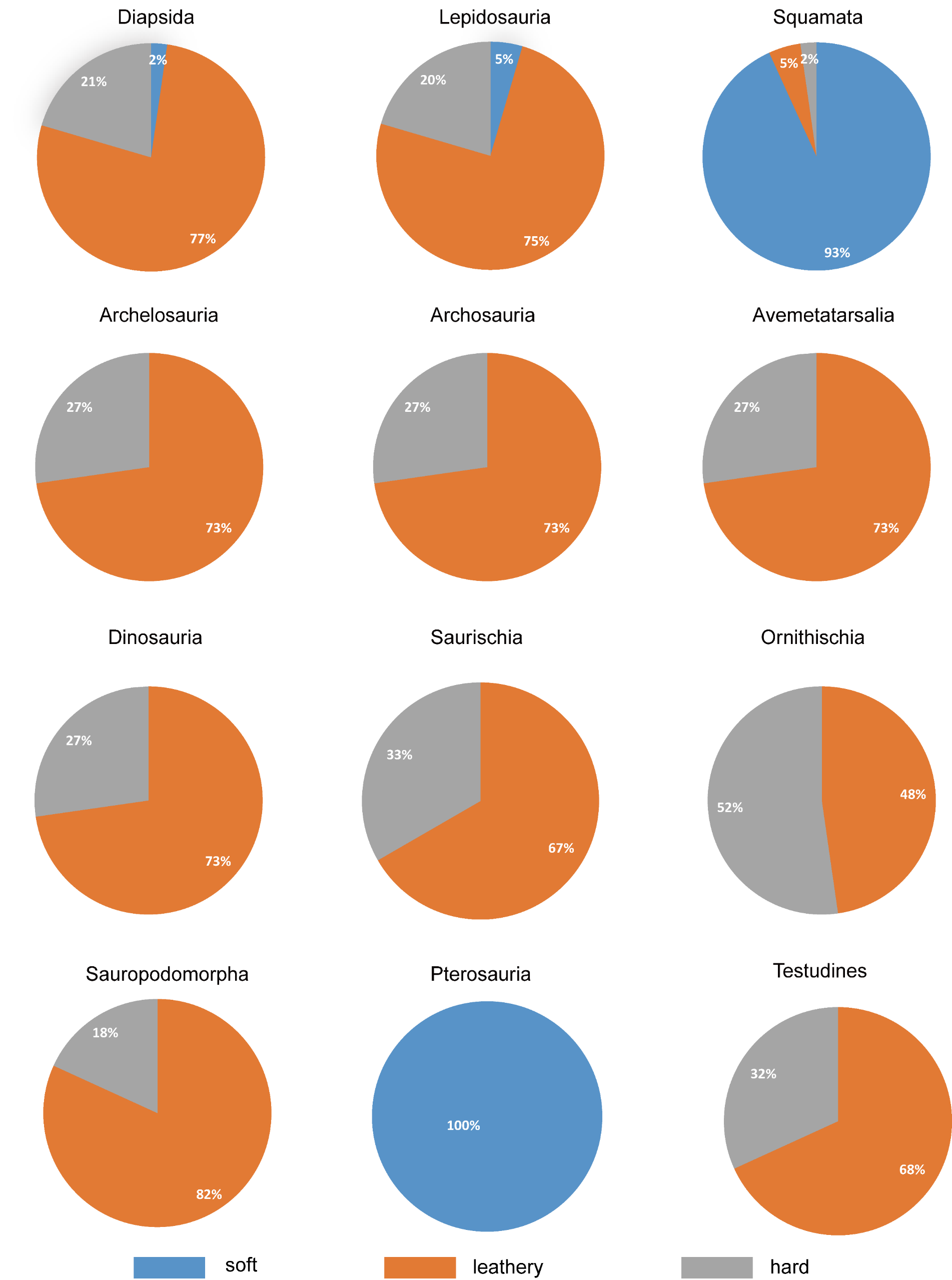


### **Supplementary Figure** 26. Summary of ancestral eggshell type with the maximum posterior probabilities in all ASR analyses.


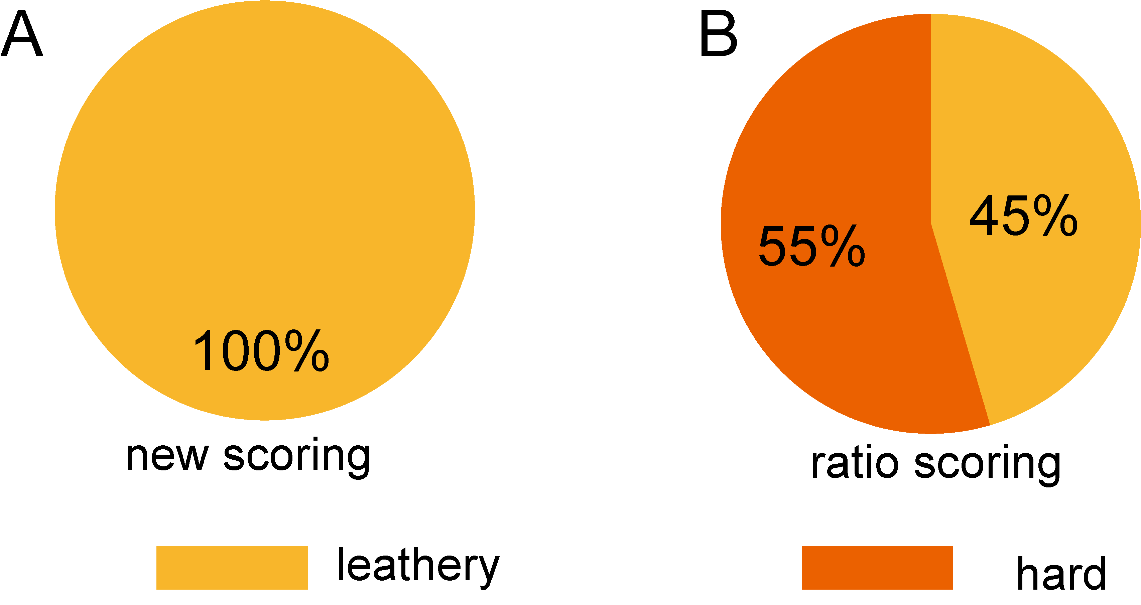


### **Supplementary Figure** 27. Egg type for the Dinosauria node based on ASR analyses using Hierarchal Bayes framework with ARD model (2 rate classes)

**
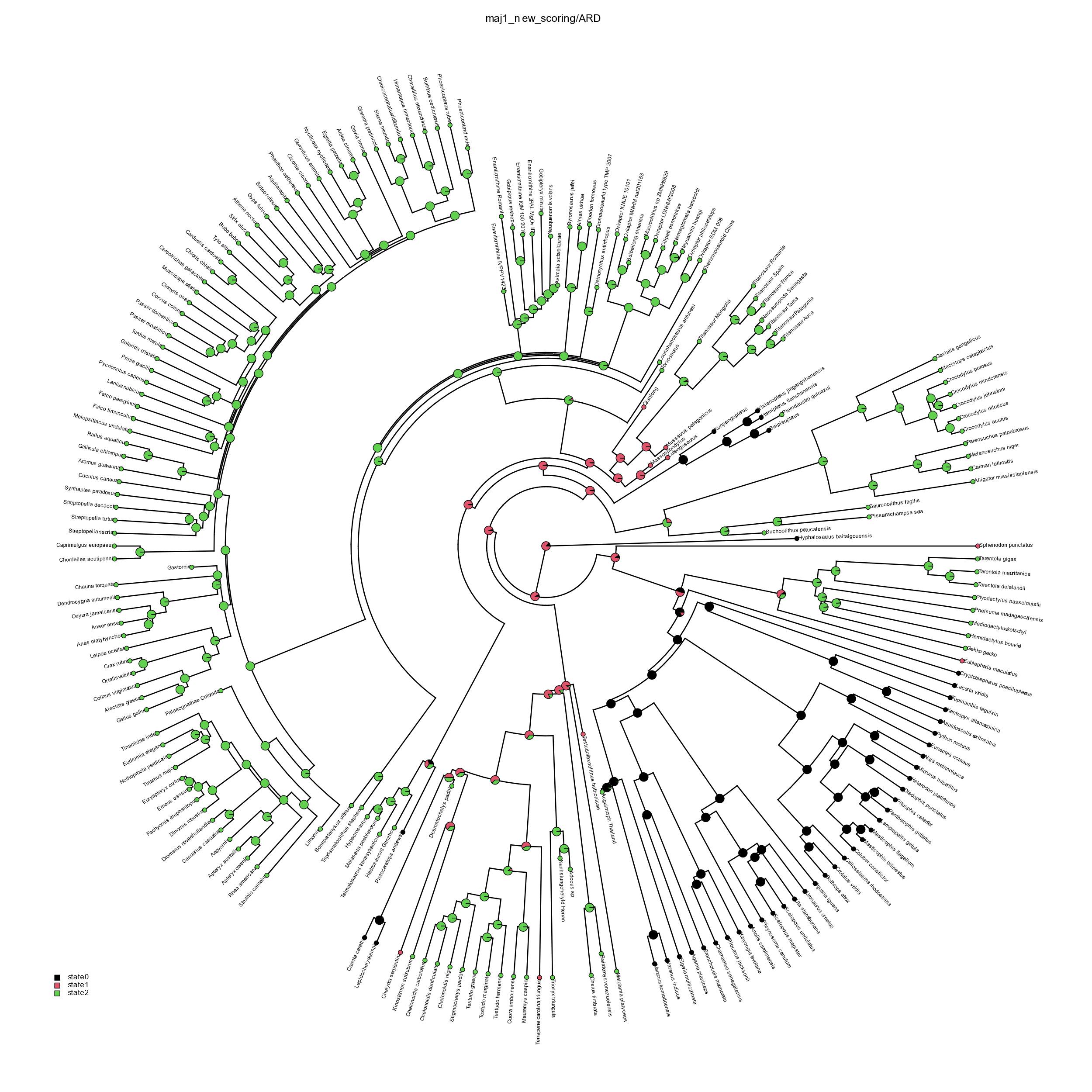
**

### **Supplementary Figure** 28. Eggshell type ASR under hierarchal Bayes framework with new scoring and ARD model (2 rate classes; using majority rule consensus tree of run1 in the first dating analysis).

State 0: soft; State1: leathery; State 2: hard.

**
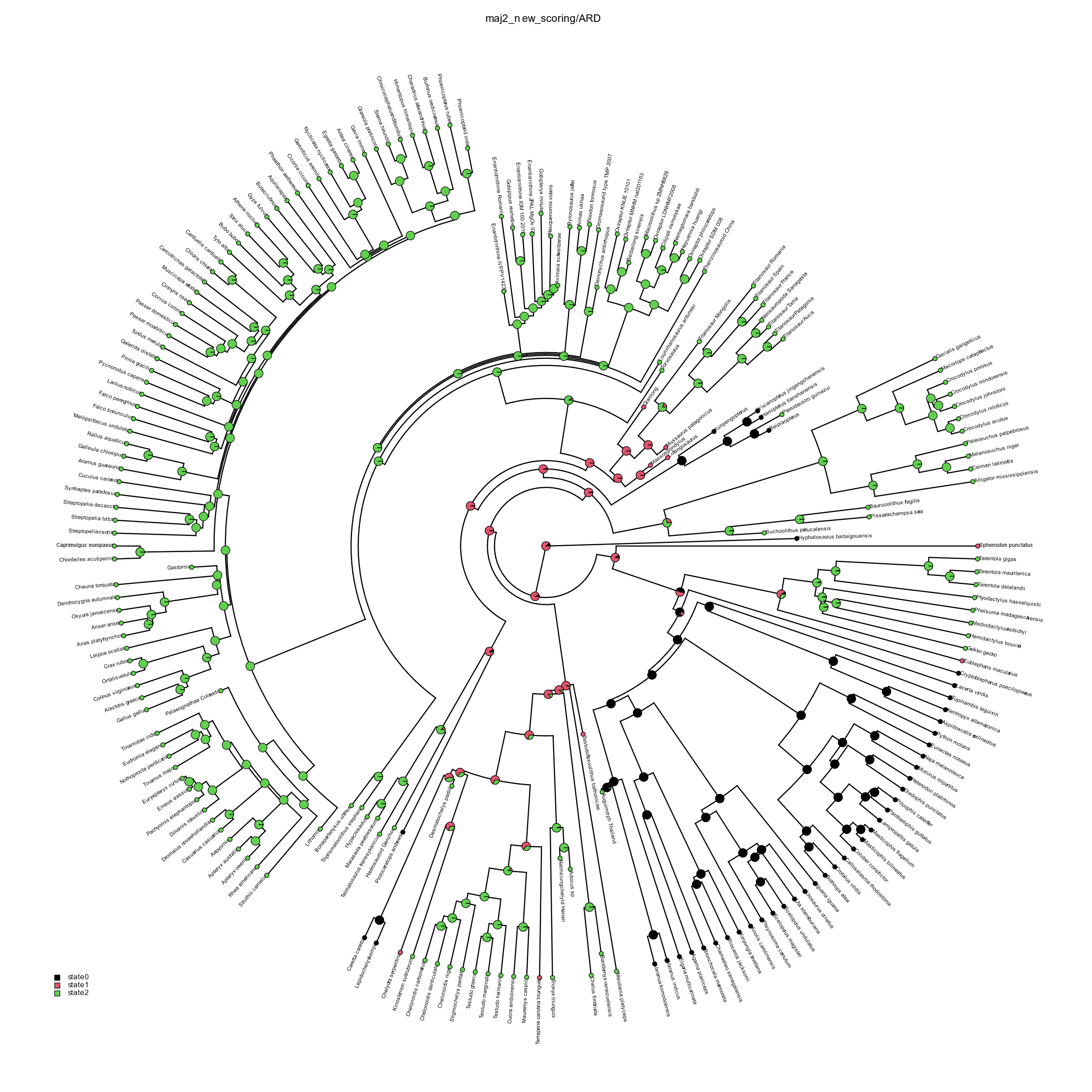
**

### **Supplementary Figure** 29. Eggshell type ASR under hierarchal Bayes framework with new scoring and ARD model (2 rate classes; using majority rule consensus tree of run2 in the first dating analysis).

State 0: soft; State 1: leathery; State 2: hard.

**
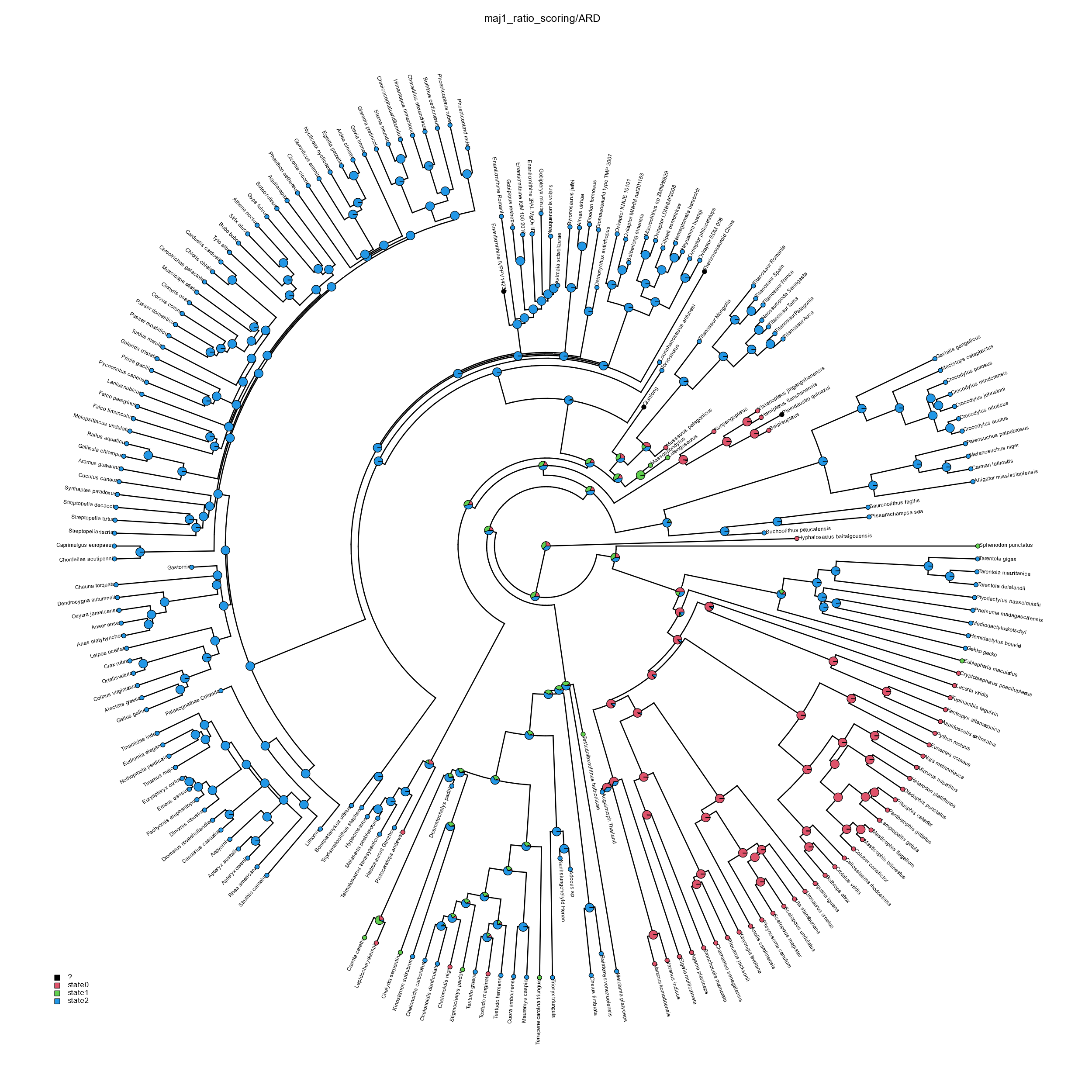
**

### **Supplementary Figure** 30. Eggshell type ASR under hierarchal Bayes framework with ratio scoring and ARD model (2 rate classes; using majority rule consensus tree of run1 in the first dating analysis).

State 0: soft; State 1: leathery; State 2: hard.

**
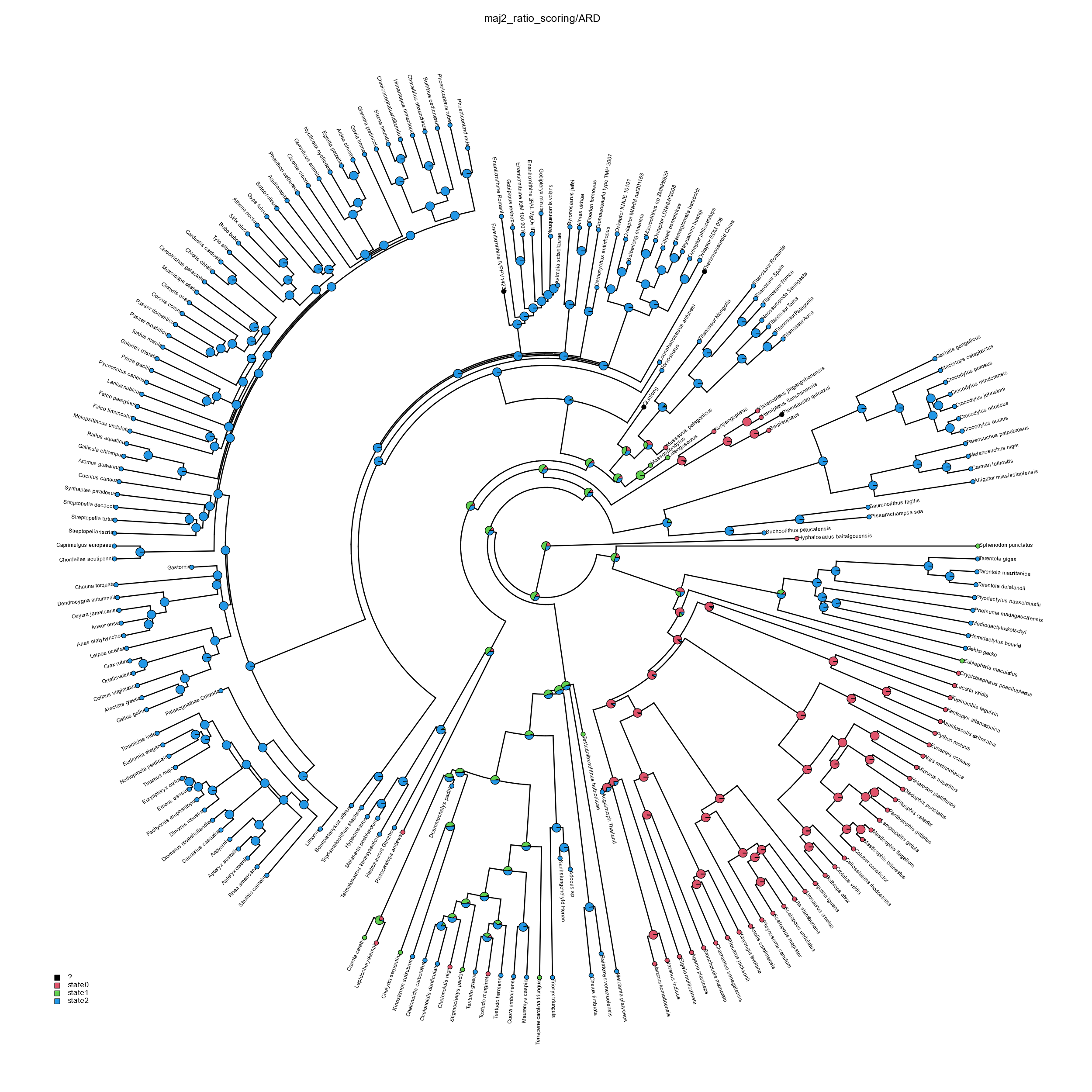
**

### **Supplementary Figure** 31. Eggshell type ASR under hierarchal Bayes framework with ratio scoring and ARD model (2 rate classes; using majority rule consensus tree of run2 in the first dating analysis).

State 0: soft; State 1: leathery; State 2: hard.

### **Supplementary Figure** 32. Phylogenetic linear regression between log_10_ transformed egg volume and body mass, and between log_10_ transformed eggshell thickness and egg volume.

Grey dashed lines represent regression lines with the 22 time-scaled trees.

## Supplementary Tables

### Supplementary Table 1. Main measurements of the adult and embryo of *Qianlong*

| Specimen number | Length | measurements (cm) |
| --- | --- | --- |
| GZPM VN001 (Holotype) | Skull length (from the anterior end to the distal end of the mandible) | 35.6 |
|  | Right scapula | 45.9 |
|  | Right coracoid | 16.9 |
|  | Left scapula | 49.9 |
|  | Left coracoid | 16.8 |
|  | Left humerus | 37.0 |
|  | Left radius | 20.5 |
|  | Left metacarpal 1 | 5.82 |
|  | Left metacarpal 2 | 8.55 |
|  | Left metacarpal 3 | 8.05 |
|  | Left metacarpal 4 | 7.10 |
|  | Right ischium | 49.5 |
|  | Left ischium | 50.8 |
|  | Left femur | 74.8 |
|  | Left tibia | 59.0 |
|  | Left metatarsal 1 | 15.1 |
|  | Left metatarsal 2 | 21.6 |
|  | Left metatarsal 3 | 24.0 |
|  | Left metatarsal 4 | 22.5 |
|  | Left metatarsal 5 | 11.6 |
|  | Right metatarsal 1 | 15.0 |
|  | Right metatarsal 2 | 21.0 |
|  | Right metatarsal 3 | 24.5 |
|  | Right metatarsal 4 | 22.0 |
|  | Right metatarsal 5 | 13.0 |
| GZPM VN002 | Left ilium | 43.9 |
|  | Pubis | 45.7 |
|  | Ischium | 40.1 |
|  | Left femur | 62.2 |
| GZPM VN004-2 (embryo) | mandible | 3.20 |
|  | maxilla | 1.62 |
|  | Scapula | 2.92 |
|  | Left ilium | >1.7 |
|  | Right ilium | >1.9 |
|  | Left ischium | 2.08 |
|  | Right pubis | 1.80 |
|  | Left pubis | 1.86 |
|  | Right ischium | 1.90 |
|  | Left femur | 2.96 |
|  | Right femur | 2.90 |
|  | Left tibia | 2.80 |
|  | Right tibia | 2.70 |
|  | Right fibula | 2.70 |
|  | Left fibula | 2.62 |
| GZPM VN006-1 (embryo) | Left scapula | 2.60 |
|  | Left humerus | 2.48 |
|  | Left ilium | 1.79 |
|  | Left femur | >2.7, 2.9^e^ |
|  | Left tibia | 2.60 |
|  | Left fibula | 2.60 |
| GZPM VN006-2 (embryo) | Left scapula | >2.54 |
|  | Left humerus | >2.11, 2.4^e^ |
|  | Left femur | >2.63, 2.9^e^ |
|  | Right femur | >2.8, 2.9 ^e^ |
|  | Left tibia | >2.4, 2.6 ^e^ |
|  | Right tibia | >2.5, 2.6 ^e^ |
|  | Left fibula | >2.4, 2.6 ^e^ |
|  | Right fibula | >2.5, 2.6 ^e^ |
| GZPM VN006-I1 (embryo) | Left humerus | 2.30 |
| GZPM VN006-I2 (embryo) | Left femur | 2.90 |
| GZPM VN006-I3 (embryo) | Right femur | 2.96 |

Notes: “e” denotes estimated values. The distal ends of the humeri and femura were slightly damaged due to spongy condyles. Their values can be estimated when compared to other complete humeri and femora.

### Supplementary Table 2. Results of δ^13^C_org_, δ^13^C (VPDB), δ^18^O (VPDB) analyses in Pingba Section and fossil beds.

| Samples | Strata height (m) | δ^13^C_org_ | δ^13^C (VPDB, ‰) | δ^18^O (VPDB, ‰) |
| --- | --- | --- | --- | --- |
| Section 1 | | | | |
| s-1 | 0.00 | -22.98 | -9.49 | -6.44 |
| s-2 | 0.36 | -22.02 | -8.36 | -5.45 |
| s-3 | 0.54 | -22.01 | — | — |
| s-4 | 0.85 | -22.01 | -9.01 | -3.79 |
| s-5 | 1.27 | -22.73 | -9.25 | -5.46 |
| s-6 | 1.43 | -21.57 | -8.77 | -6.07 |
| s-7 | 1.58 | -22.39 | -9.65 | -6.48 |
| s-8 | 1.70 | -23.02 | -8.42 | -5.13 |
| s-9 | 1.76 | -22.23 | -8.70 | -6.10 |
| s-10-11 | 1.78 | -23.00 | -7.58 | -5.84 |
| s-12 | 2.06 | -23.02 | -9.04 | -6.98 |
| s-13 | 2.18 | -21.41 | -11.73 | -7.30 |
| s-14 | 2.30 | -23.04 | -9.54 | -6.66 |
| s-15 | 2.58 | -21.58 | -10.66 | -6.70 |
| s-16 | 2.75 | -21.49 | -10.61 | -7.02 |
| s-17 | 2.95 | -22.17 | -9.50 | -6.60 |
| s-18 | 3.10 | -21.72 | -9.95 | -7.13 |
| s-19 | 3.27 | -23.06 | — | — |
| s-20 | 3.55 | -21.36 | -7.85 | -6.11 |
| Section 2 | | | | |
| S-21 | 0.00 | -21.46 | -9.10 | -6.14 |
| S-22 | 0.21 | -21.65 | -10.74 | -6.99 |
| S-23 | 0.38 | -21.30 | -9.67 | -5.97 |
| S-24 | 0.55 | -21.80 | -9.63 | -6.86 |
| S-25 | 0.75 | -21.79 | -10.54 | -7.00 |
| S-26 | 0.96 | -23.08 | -10.19 | -6.36 |
| S-27 | 1.13 | -21.60 | -9.48 | -6.05 |
| S-28 | 1.20 | -21.37 | -9.37 | -5.81 |
| S-29 | 1.37 | -21.30 | -8.39 | -5.56 |
| S-30 | 1.47 | -21.26 | -8.87 | -5.28 |
| Fossil beds | | | | |
| VN002-S1 |  | -22.07 | -9.01 | -5.49 |
| VN002-S2 |  | -21.33 | -8.04 | -5.33 |
| VN002-S3 |  | -21.46 | -9.36 | -5.45 |
| VN001-S2 |  | -21.50 | -9.43 | -6.18 |
| VN001-S1 |  | -21.36 | -9.26 | -6.26 |
| VN007-S1 |  | -21.99 | -10.11 | -6.59 |
| VN004-S1 |  | -21.35 | -9.91 | -6.42 |
| VN005-S1 |  | -22.18 | -8.48 | -6.33 |
| VN002-S4 |  | — | -8.93 | -5.63 |

### Supplementary Table 3. Character coding of *Qianlong shouhu.*

| **1-10** | **11-20** | **21-30** | **31-40** | **41-50** |
| --- | --- | --- | --- | --- |
| 10?1?002?? | 00211?111? | ????1111?? | 1??000???? | ?????????? |
| **51-60** | **61-70** | **71-80** | **81-90** | **91-100** |
| ?????????? | ?????????? | ?????????? | ?????????? | ???1?1?01? |
| **101-110** | **111-120** | **121-130** | **131-140** | **141-150** |
| 0?????0?00 | ?10?211?0? | ?????????? | ?????????? | ?1?01?00?0 |
| **151-160** | **161-170** | **171-180** | **181-190** | **191-200** |
| 011?00?100 | 0000000100 | 001??????1 | 011?001??1 | ????0?0?00 |
| **201-210** | **211-220** | **221-230** | **231-240** | **241-250** |
| 0011012000 | 111??????? | ?001113101 | 1?120?0000 | 020?001001 |
| **251-260** | **261-270** | **271-280** | **281-290** | **291-300** |
| 310[01]0??001 | 1?1100?011 | 1001?111?0 | 01011110?0 | 0101101010 |
| **301-310** | **311-320** | **321-330** | **331-340** | **341-350** |
| ????0????? | ?????????? | ?????????0 | 1011?0012? | 011100001? |
| **351-360** | **361-370** | **371-380** | **381-390** | **391-400** |
| 003???0?1? | 1???0???01 | ?10000?101 | 111?1100?0 | ?1???????? |
| **401-410** | **411-420** |  |  |  |
| ?????????? | ??????1?? |  |  |  |

### Supplementary Table 4. Collection of limb bone measurements in basal sauropodomorphs and sauropods.

| **Taxa** | **Group** | **Specimen number** | **Stages** | **Humeral length (cm)** | **femur length (cm)** | **Ratio (H/F)** | **References** |
| --- | --- | --- | --- | --- | --- | --- | --- |
| *Qianlong shouhu* | Sauropodomorph | GZPM VN001 | adult | 37 | 74.8 | 0.49 | this study |
| *Qianlong shouhu* | Sauropodomorph | GZPM VN006-1 | embryo | 2.40 | 2.90 | 0.83 | this study |
| *Qianlong shouhu* | Sauropodomorph | GZPM VN006-2 | embryo | 2.48 | 2.90 | 0.85 | this study |
| *Massospondylus carinatus* | Sauropodomorph | BP/1/5347A | embryo | 0.95 | 1.15 | 0.83 | Reisz *et al.* [10] |
| *Massospondylus carinatus* | Sauropodomorph | BP/1/5253 | ? | 7.9 | 12.75 | 0.62 | Reisz *et al.* [10] |
| *Massospondylus carinatus* | Sauropodomorph | TM 130 | ? | 8 | 13.7 | 0.58 | Reisz *et al.* [10] |
| *Massospondylus carinatus* | Sauropodomorph | SAM 388 | ? | 13.6 | 23.1 | 0.59 | Reisz *et al.* [10] |
| *Massospondylus carinatus* | Sauropodomorph | SAM 391 | ? | 15.5 | 24.8 | 0.63 | Reisz *et al.* [10] |
| *Massospondylus carinatus* | Sauropodomorph | BP/1/4779 | ? | 17.5 | 32 | 0.55 | Reisz *et al.* [10] |
| *Massospondylus carinatus* | Sauropodomorph | BP/1/4998 | ? | 17.5 | 34 | 0.51 | Reisz *et al.* [10] |
| *Massospondylus carinatus* | Sauropodomorph | SAM 5135 | ? | 22 | 35 | 0.63 | Reisz *et al.* [10] |
| *Massospondylus carinatus* | Sauropodomorph | BP/1/5241 | ? | 23 | 36 | 0.64 | Reisz *et al.* [10] |
| *Massospondylus carinatus* | Sauropodomorph | BP/1/4934 | adult | 27.5 | 55 | 0.5 | Reisz *et al.* [10] |
| *Mussaurus patagonicus* | Sauropodomorph | PVL 4068 | embryo | 2.7 | 3 | 0.9 | Bonaparte and Vince [131], Otero *et al.* [132] |
| *Mussaurus patagonicus* | Sauropodomorph | MACN 4111 | embryo | 2.35 | 2.7 | 0.87 | Otero and Pol [123] |
| *Mussaurus patagonicus* | Sauropodomorph | MPM 1813 | juvenile | 9.5 | 11.2 | 0.85 | Otero and Pol [123] |
| *Mussaurus patagonicus* | Sauropodomorph | MLP 68-II-27-1 | Adult | 46 | 77 | 0.60 | Otero and Pol [123] |
| *Brachiosaurus altithorax* | Titanosauriforms | SMA 0009 | juvenile | 18.16 | 22.48 | 0.81 | Schwarz *et al.* [133], Carballido *et al.* [134] |
| *Brachiosaurus altithorax* | Titanosauriforms | NA | adult | 200.04 | 200.03 | 1.00 | Riggs [135] |
| *Apatosaurus louisa* | Diplodocidae | CM 3018 | ? | 115 | 178.5 | 0.64 | Gilmore [136] |
| *Apatosaurus louisa* | Diplodocidae | CM 563 | ? | 110 | 171 | 0.64 | Gilmore [136] |
| *Rapetosaurus krausei* | Titanosauria | FMNH PR 2209 | juvenile | 52.5 | 65.7 | 0.80 | Rogers [137] |
| *Rapetosaurus krausei* | Titanosauria | UA 9998 | perinatal | 15.87 | 19.2 | 0.83 | Rogers [137] |
| *Camarasaurus grandis* | Camarasauridae | GMNH-PV 101 | ? | 113 | 148.5 | 0.76 | McIntosh *et al.* [138] |
| *Camarasaurus grandis* | Camarasauridae | YPM 1901 | ? | 89 | 118 | 0.75 | McIntosh *et al.* [138] |
| *Camarasaurus grandis* | Camarasauridae | YPM 1905 | ? | 81.6 | 112.8 | 0.73 | McIntosh *et al.* [138] |
| *Camarasaurus lentus* | Camarasauridae | YPM 1910 | Juvenile | 54.5 | 75 | 0.73 | McIntosh *et al.* [138] |
| *Camarasaurus lentus* | Camarasauridae | CM 11338 | juvenile | 43.5 | 57 | 0.76 | McIntosh *et al.* [138] |
| *Camarasaurus lentus* | Camarasauridae | WDC A (BS-156) | ? | 103.1 | 140.8 | 0.73 | Ikejiri [139] |
| *Camarasaurus lentus* | Camarasauridae | WDC B | ? | 114 | 145.3 | 0.78 | Ikejiri [139] |
| *Camarasaurus lentus* | Camarasauridae | USNM 13786 | ? | 80.8 | 89.7 | 0.90 | Ikejiri [139] |
| *Camarasaurus lentus* | Camarasauridae | CM 11393 | ? | 119 | 156.8 | 0.76 | Ikejiri [139] |

### Supplementary Table 5. List of variables and equations used for this study and analysis results.

| Variable | Definition | Equation | Measurement | Unit |
| --- | --- | --- | --- | --- |
| A | Average individual pore area | — | 85.08 | μm^2^ |
| D | Pore density | — | 10.43 | mm^-2^ |
| L*d | Egg diameter | — | 115*94 | mm |
| Ls | Eggshell thickness(=pore length) | — | 0.230 | mm |
| V | Egg volume | 0.524·Ld^2^ | 532457 | mm^3^ |
| M | Egg mass | 5.48·10^-4^·Ld^2^ | 556.84 | g |
| As | Surface area of eggshell | 4.951·V^0.666^ | 29895.99 | mm^2^ |
| Ap | Total pore area | A·As·D | 26.53 | mm^2^ |
| Ap·Ls^-1^ | Eggshell porosity | — | 115.35 | mm |

### Supplementary Table 6. Collections of eggshell thickness in 22 extant birds.

| Avian | Eggshell Thickness (μm) | | | References |
| --- | --- | --- | --- | --- |
|  | crystalline layer | membrane | total |  |
| *Grus antigone antigone* | 650 | 110 | 760 | Hirsch [79] |
| *Phoenicopterus r. ruber* | 560 | 70 | 630 | Hirsch [79] |
| *Phasianus colchicus* | 240 | 40 | 280 | Kirici *et al.* [140] |
| *Dromaius americana* | 1000 | 70 | 1070 | Dauphin *et al.* [141] |
| *Dromaius novaehollandiae* | 970 | 140 | 1110 | This study |
| *Struthio camelus* | 1760 | 180 | 1940 | This study |
| *Melopsittacus undulatus* | 90 | 60 | 150 | This study |
| *Nymphicus hollandicus* | 120 | 70 | 190 | This study |
| *Columba livia* | 170 | 50 | 220 | This study |
| *Meleagris gallopavo* | 330 | 70 | 400 | This study |
| *Numida meleagris* | 540 | 70 | 610 | This study |
| *Anser anser* | 480 | 40 | 520 | This study |
| *Anser cygnoides* | 520 | 40 | 560 | This study |
| *Francolinus pintadeanus* | 210 | 60 | 270 | This study |
| *Phasianus colchicus* | 300 | 110 | 410 | This study |
| *Gallus gallus* | 320 | 40 | 360 | This study |
| *Poephila guttata* | 70 | 20 | 90 | This study |
| *Anas platyrhynchos* | 350 | 40 | 390 | This study |
| *Synoicus chinensis* | 120 | 40 | 160 | This study |
| *Pavo cristatus* | 460 | 90 | 550 | This study |
| *Cairna* sp. | 400 | 70 | 470 | This study |
| *Coturnix* sp. | 170 | 20 | 190 | This study |

### Supplementary Table 7. Measurements of average eggshell fragments length in some extant and extinct taxa.

| Higher Taxa | Taxa | Specimen number | Number of measurements | Average length (μm) | Egg volume (mm^3^) | Eggshell type |
| --- | --- | --- | --- | --- | --- | --- |
| Testudines | *Pseudemys nelsoni* | — | 228 | 630 | 4024 | leathery |
| Testudines | *Chrysemys picta* | — | 50 | 400 | 9538 | leathery |
| Testudines | *Chelydra serpentina* | — | 209 | 1000 | 10889 | leathery |
| Testudines | *Mauremys reevesii* | — | 33 | 1820 | 8319 | leathery |
| Testudines | *Kinosternon subrubrum* | — | 32 | — | 4800 | Rigid |
| Testudines | *Trionyx sinensis* | — | 47 | 5830 | 2185 | Rigid |
| Testudines | *Chelodina oblonga* | — | 10 | 8730 | 11104 | Rigid |
| Testudines | *Chinemys reevesii* | — | 55 | 4120 | 10884 | Rigid |
| Dinosauria | *Elongatoolithus magnus* | CUGW EH023 | 239 | 16800 | 604474 | Rigid |
| Dinosauria | *Shixingoolithus erbeni* | CUGW EH024 | 151 | 26220 | 991078 | Rigid |
| Dinosauria | *Neixiangoolithus yani* | CUGW EH039 | 352 | 16010 | 86431 | Rigid |
| Dinosauria | *Dendroolithus* sp*.* | CUGW EH050 | 124 | 9580 | 2144661 | Rigid |
| Dinosauria | *Placoolithus tumiaolingensis* | CUGW EH073 | 41 | 19800 | 1541213 | Rigid |
| Dinosauria | *Qianlong shouhu* | — | 245 | 1360 | 532457 | Rigid |
| Aviae | *Struthio camelus* | — | 53 | 22730 | 957794 | Rigid |
| Aviae | *Dromaius novaehollandiae* | — | 37 | 29170 | 474749 | Rigid |
| Aviae | *Coturnix coturnix* | — | 67 | 7130 | 10379 | Rigid |
| Aviae | *Gallus gallus domesticus* | — | 78 | 11330 | 44459 | Rigid |
| Aviae | *Anas platyrhynchos domesticus* | — | 93 | 11180 | 56158 | Rigid |
| Aviae | *Melopsittacus undulatus* | — | 18 | 4580 | 1988 | Rigid |
| Aviae | *Anser cygnoides* | — | 24 | 14720 | 161042 | Rigid |
| Aviae | Columbia livia | — | 75 | 5710 | 15987 | Rigid |
| Aviae | *Poephila guttata* | — | 85 | 1810 | 1061 | Rigid |
| Aviae | *Anser anser* | — | 61 | 13070 | 122970 | Rigid |
| Aviae | *Cairina* | — | 29 | 13190 | 82213 | Rigid |
| Aviae | *Erythrura gouldiae* | — | 52 | 1750 | 1291 | Rigid |
| Aviae | *Synoicus* | — | 17 | 5610 | 5845 | Rigid |
| Aviae | *Francolinus pintadeanus* | — | 17 | 10190 | 16584 | Rigid |
| Aviae | *Paroedura pictus* | — | 18 | 3270 | 681 | Rigid |

### Supplementary Table 8. Collection of leathery egg data of extant reptiles for analyzing eggshell type.

| Classification | Species | Calcareous layer thickness (µm) | Total eggshell thickness | Egg mass (g) | References |
| --- | --- | --- | --- | --- | --- |
| Pelomedusidae | *Pelusios castaneus* | 75 | 146 | 11.8 | Kusuda *et al.* [45], Bour *et al.* [142] |
| Chelonians | *Chelonia mydas* | 130 | _ | 51.65 | Youngsabanant and Nuamsukon [143], Elgar and Heaphy [144] |
| Chelonians | *Lepidochelys olivacea* | 70 | 125 | 35.94 | Elgar and Heaphy [144], Sahoo *et al.* [145] |
| Chelonians | *Chelydra serpentina* | 161 | 268 | 11.44 | Kusuda *et al.* [45], [146] |
| Chelonians | *Graptemys flavimaculata* | 97 | 171 | 15 | Kusuda *et al.* [45], Selman and Jones [147] |
| Chelonians | *Terrapene carolina* | 130 | 235 | 10.57 | Schleich and Kästle [78], Elgar and Heaphy [144] |
| Chelonians | *Geoemyda japonica* | 96 | 175 | 16 | Kusuda *et al.* [45], Yasukawa and Ota [148] |
| Chelonians | *Mauremys japonica* | 105 | _ | 13.27 | Kusuda *et al.* [45], Okada *et al.* [149] |
| Chelonians | *Caretta caretta* | 84 | 154 | 34.4 | Kusuda *et al.* [45], Elgar and Heaphy [144] |
| Emydidae | *Trachemys scripta elegans* | 108 | 146 | 10.80 | Kusuda *et al.* [45], Elgar and Heaphy [144] |
| Geoemydidae | *Mauremys sinensis* | 91 | 232 | 8.90 | This study |
| Squamates | *Eublepharis macularius* | 36 | 71 | 3 | Legendre *et al.* [7], Choi *et al.* [127] |

### Supplementary Table 9. Dataset used for reproduction evolution analyses.

**ET(N)**, Eggshell type (new scoring); 0, soft; 1, leathery; 2, hard. **EN(R),** Eggshell type (ratio scoring); 0, 0-50%; 1, 50%-66.7%; 2, >66.7%. **ET(U)**, Eggshell unit; 0, absent; 1, present and spike-like; 2, present and column shaped. **ET(F)**, Eggshell type (Fracture): 0, absent; 1, present, very small pieces; 2. large pieces. **EI,** elongation index. **RC**, Ratio of calcareous layer to total eggshell thickness. **RU**, Eggshell unit (ratio of height to width). **CL**, thickness of calcareous layer (µm, average), **BM**, body mass (kg). **EV,** egg volume (mm^3^). **CS**, Clutch size or number of per clutch. **CV**, Clutch volume (mm^3^)

| **Taxon** | **Age (Ma)** | **ET (N)** | **EN (R)** | **ET (U)** | **ET (F)** | **EI** | **RC** | **RU** | **CL** | **BM** | **EV** | **CS** | **CV** | **References** |
| --- | --- | --- | --- | --- | --- | --- | --- | --- | --- | --- | --- | --- | --- | --- |
| *Hyphalosaurus baitaigouensis* | 129.7-122.3 | 0 | 0 | 0 | 0 | 1.34 | NA | NA | 10 | NA | 2148 | NA | NA | Hou *et al.* [44] |
| *Suchoolithus portucalensis* | 152-145 | 2 | 2 | 2 | 2 | 1.62 | NA | 1 | 160 | NA | 14877 | 13 | 193406 | Russo *et al.* [36] |
| *Pissarrachampsa sera* | 83.6-66 | 2 | 2 | 2 | 2 | 1.71 | NA | 1.35 | 150 | NA | 66552 | 4 | 266209 | Marsola *et al.* [37] |
| *Bauruoolithus fragilis* | 83.6-66 | 2 | 2 | 2 | 2 | 1.81 | 0.76 | 0.86 | 200 | NA | 44142 | 4 | 176567 | Oliveira *et al.* [150] |
| *Alligator mississippiensis* | 0 | 2 | 2 | 2 | 2 | 1.70 | 0.74 | 1.2 | 530 | 47.8 | 69171 | 40 | 2766850 | Thorbjarnarson [46], Marzola *et al.* [61], Ferguson [77], Brazaitis and Watanabe [151] |
| *Caiman latirostris* | 0 | 2 | 2 | 2 | 2 | 1.48 | 0.77 | 1.3 | 540 | 14.6 | 63807 | 45 | 2871330 | Thorbjarnarson [46], Marzola *et al.* [61], Schleich and Kästle [78], Brazaitis and Watanabe [151] |
| *Crocodylus acutus* | 0 | 2 | 2 | 2 | 2 | 1.60 | 0.75 | NA | 450 | 76.7 | 95205 | 45 | 4284226 | Thorbjarnarson [46], Marzola *et al.* [61], Hirsch [79], Brazaitis and Watanabe [151] |
| *Crocodylus johnstoni* | 0 | 2 | 2 | 2 | 2 | 1.49 | NA | 1 | 400 | 19.5 | 59152 | 13 | 768974 | Thorbjarnarson [46], Marzola *et al.* [61], Hirsch and Kohring [80], Brazaitis and Watanabe [151] |
| *Crocodylus mindorensis* | 0 | 2 | 2 | 2 | 2 | 1.86 | NA | 1.1 | 430 | 36.9 | 50522 | 15 | 757833 | Thorbjarnarson [46], Marzola *et al.* [61], Ferguson [62], Brazaitis and Watanabe [151] |
| *Crocodylus niloticus* | 0 | 2 | 2 | 2 | 2 | 1.61 | 0.57 | NA | 530 | 94.2 | 93083 | 53 | 4933374 | Thorbjarnarson [46], Marzola *et al.* [61], Brazaitis and Watanabe [151] |
| *Crocodylus porosus* | 0 | 2 | 2 | 2 | 2 | 1.58 | 0.64 | NA | 553 | 78.7 | 109150 | 50 | 5457489 | Thorbjarnarson [46], Marzola *et al.* [61], Hirsch and Kohring [80], Brazaitis and Watanabe [151] |
| *Gavialis gangeticus* | 0 | 2 | 2 | 2 | 2 | 1.46 | NA | NA | 445 | 147 | 134748 | 40 | 5389906 | Thorbjarnarson [46], Marzola *et al.* [61], Brazaitis and Watanabe [151], Blas and Patnaik [152] |
| *Melanosuchus niger* | 0 | 2 | 2 | 2 | 2 | 1.70 | 0.94 | 1.3 | 800 | 82 | 56194 | 48 | 2697288 | Thorbjarnarson [46], Ferguson [62], Schleich and Kästle [78], Brazaitis and Watanabe [151] |
| *Paleosuchus_palpebrosus* | 0 | 2 | 2 | 2 | 2 | 1.65 | NA | 1.1 | 410 | 5.9 | 56593 | 15 | 848896 | Thorbjarnarson [46], Marzola *et al.* [61], Brazaitis and Watanabe [151] |
| *Mecistops cataphractus* | 0 | 2 | 2 | 2 | 2 | 1.60 | NA | NA | 450 | 50.5 | 117602 | 19 | 2234445 | Thorbjarnarson [46], Marzola *et al.* [61], Hirsch and Kohring [80], Brazaitis and Watanabe [151] |
| **Pterosaurs** | | | | | | | | | | | | | | |
| *Beipiaopterus* | 129.4-122.46 | 0 | 0 | 0 | 0 | 1.75 | 0.00 | NA | 0 | 0.8 | 43044 | NA | NA | Ji *et al.* [28], Witton [49], Lü [51] |
| *Kunpengopterus* | 167.7-150.8 | 0 | 0 | 0 | 0 | 1.40 | 0.00 | NA | 0 | 0.22 | 5712 | NA | NA | Lü *et al.* [31], Wang *et al.* [32] |
| *Hamipterus tianshanensis* | 145-100.5 | 0 | 0 | 0 | 0 | 2.02 | 0.00 | NA | 0 | 5.37 | 21174 | NA | NA | Wang *et al.* [33], Witton [49], Wang *et al.* [64] |
| *Yixianopterus jingangshanensis* | 129.4-122.46 | 0 | 0 | 0 | 0 | 1.29 | 0.00 | NA | NA | 3 | 45437 | NA | NA | Wang and Zhou [29], Unwin and Deeming [30], Witton [49], Jiang *et al.* [50] |
| *Pterodaustro guinazui* | 125-100.5 | 2 | NA | 2 | 2 | 2.73 | NA | NA | 50 | 0.87 | 14810 | NA | NA | Chiappe *et al.* [26], Grellet-Tinner *et al.* [27], Witton [49], Codorniú *et al.* [153] |
| **Dinosaurs** | | | | | | | | | | | | | | |
| *Lufengosaurus* | 201.3-190.8 | 1 | 1 | 2 | 1 | NA | 0.53 | 0.82 | 75 | 2259 | NA | NA | NA | Stein *et al.* [8], Benson *et al.* [52], Reisz *et al.* [109] |
| *Mussaurus patagonicus* | 192.78-192.74 | 1 | 0 | NA | 2 | 1.21 | NA | NA | 0 | 1504.76 | 123392 | 30 | 3701760 | Pol et al. Pol *et al.* [1], Benson *et al.* [52], Durand [75], Bonaparte and Vince [131] |
| *Massospondylus* | 227-190.8 | 1 | 1 | NA | 2 | 1.00 | 0.56 | NA | 90 | 487.74 | 113097 | 34 | 3845298 | Stein *et al.* [8], Reisz *et al.* [10], Reisz *et al.* [87] |
| *Qianlong shouhu* | 190.8-174.1 | 1 | NA | 2 | 1 | 1.22 | NA | 3 | 161 | 1057 | 532457 | 16 | 8519312 | This study |
| Titanosaur_Auca | 83.6-79.5 | 2 | 2 | 2 | 2 | 1.15 | 0.73 | 1.76 | 955 | NA | 914747 | 40 | 36589872 | Moore and Varricchio [5], Chiappe *et al.* [15], Chiappe *et al.* [66] |
| Titanosaur_France | 83.6-66 | 2 | 2 | 2 | 2 | 1.00 | NA | 1.75 | 1700 | NA | 4849048 | 15 | 72735724 | Sander *et al.* [154], Fernández and Khosla [155] |
| Titanosaur_Spain | 83.6-66 | 2 | 2 | 2 | 2 | 1.31 | NA | 3.05 | 2535 | NA | 1759292 | 28 | 49260176 | Moore and Varricchio [5], Vila *et al.* [156] |
| Titanosaur_Tama | 100.5-66 | 2 | 2 | 2 | 2 | 1.00 | NA | NA | 1495 | NA | 2573460 | 21 | 54042660 | Hechenleitner *et al.* [68] |
| Neosauropoda_Sanagasta | 100.5-66 | 2 | 2 | 2 | 2 | 1.00 | NA | NA | 3847 | NA | 4849048 | 35 | 169716680 | Hechenleitner *et al.* [68], Grellet-Tinner and Fiorelli [157] |
| Titanosaur_Mongolia | 145-100.5 | 2 | 2 | 2 | 2 | 1.00 | NA | 3.5 | 1200 | NA | 369121 | NA | NA | Grellet-Tinner *et al.* [69] |
| Titanosaur_Patagonia | 100.5-66 | 2 | 2 | 2 | 2 | NA | NA | 2.9 | 1800 | NA | NA | NA | NA | Kundrát *et al.* [158] |
| Titanosaur_Romania | 72.1-66 | 2 | 2 | 2 | 2 | 1.00 | 0.90 | NA | 1750 | NA | 904779 | 5 | 4523895 | Grellet-Tinner *et al.* [70] |
| *Hypacrosaurus* | 83.6-66 | 2 | 2 | 2 | 2 | 1.00 | NA | NA | 1150 | 3689.15 | 4188790 | 22 | 92153380 | Benson *et al.* [52], Horner [71] |
| *Maiasaura peeblesorum* | 83.6-72.1 | 2 | 2 | 2 | 2 | 1.00 | NA | NA | 1125 | 3656.37 | 904779 | 16 | 14476464 | Benson *et al.* [52], Horner [71] |
| *Telmatosaurus transsylvanicus* | 72.1-66 | 2 | 2 | 2 | 2 | 1.00 | 0.94 | 2.45 | 2300 | NA | 1767146 | 13 | 22972898 | Grigorescu [72], Grigorescu [159], Grigorescu [160] |
| Hadrosauroid_Ganzhou | 89.8-66 | 2 | 2 | 2 | 2 | 1.25 | NA | 2.5 | 370 | NA | 673511 | NA | NA | Xing *et al.* [161] |
| *Protoceratops andrewsi* | 83.6-72.1 | 0 | 0 | 0 | 0 | 2.05 | 0.00 | NA | 0 | 82.69 | 232027 | 12 | 2784324 | Norell *et al.* [6], Benson *et al.* [52] |
| *Torvosaurus* | 163.5-145 | 2 | 2 | 2 | 2 | NA | NA | 5.6 | 1200 | 2433 | NA | NA | NA | Benson *et al.* [52], Araújo *et al.* [162] |
| *Lourinhanosaurus antunesi* | 155.7-145 | 2 | 2 | 2 | 2 | 1.38 | NA | 1.77 | 920 | 238.03 | 601908 | 34 | 20464872 | Benson *et al.* [52], Ribeiro *et al.* [74], Mateus *et al.* [163] |
| Therizinosauroid_China | 85-75 | 2 | NA | NA | NA | 1.29 | NA | NA | NA | NA | 231084 | NA | NA | Benson *et al.* [52], Kundrát *et al.* [73] |
| *Bonapartenykus ultimus* | 83.6-66 | 2 | 2 | 2 | 2 | NA | 0.81 | NA | 1000 | 155.77 | NA | NA | NA | Benson *et al.* [52], Agnolin *et al.* [164] |
| *Triprismatoolithus stephensi* | 83.6-72.1 | 2 | 2 | 2 | 2 | 2.50 | NA | NA | 688 | NA | 34425 | NA | NA | Agnolin *et al.* [164] |
| Oviraptor_SDM_008 | 83.6-66 | 2 | 2 | 2 | 2 | 2.75 | NA | 6.7 | 831 | NA | 336121 | NA | NA | Shao *et al.* [165] |
| *Heyuannia huangi* | 83.6-66 | 2 | 2 | 2 | 2 | 2.27 | NA | 10 | 1600 | 43.48 | 515132 | NA | NA | Benson *et al.* [52], Cheng *et al.* [166] |
| *Citipati osmolskae* | 83.6-72.1 | 2 | 2 | 2 | 2 | 2.71 | NA | NA | 1005 | 123.99 | 411909 | 22 | 9061998 | Norell *et al.* [14], Benson *et al.* [52], Norell *et al.* [167] |
| *Oviraptor philoceratops* | 83.6-70.6 | 2 | 2 | 2 | 2 | 2.00 | NA | 4.4 | 725 | 39 | 220320 | 30 | 6609600 | Moore and Varricchio [5], Norell *et al.* [13], Osborn *et al.* [168] |
| *Nemegtomaia barsboldi* | 72.1-70.6 | 2 | 2 | 2 | 2 | 2.73 | NA | NA | 1100 | 40 | 231413 | 18 | 4165434 | Moore and Varricchio [5], Fanti *et al.* [169] |
| Oviraptor_LDNHMF2008 | 83.6-66 | 2 | 2 | 2 | 2 | 2.53 | NA | 10 | 1080 | 70.61 | 792221 | 24 | 19013304 | Bi *et al.* [54] |
| *Macroolithus*_sp(ZMNH8829) | 83.6-66 | 2 | 2 | 2 | 2 | 2.11 | NA | 6.7 | 1134 | 69.7 | 784890 | NA | NA | Jin *et al.* [55], Lü *et al.* [170] |
| *Beibeilong sinensis* | 100.5-89.8 | 2 | 2 | 2 | 2 | 2.83 | NA | 8 | 2130 | NA | 4876875 | NA | NA | Pu *et al.* [171] |
| Oviraptor_KNUE_10101 | 83.6-72.1 | 2 | 2 | 2 | 2 | 3.39 | NA | 5 | 2700 | NA | 2630453 | NA | NA | Kim *et al.* [172] |
| Oviraptor_MNHM_nat201153 | 83-77 | 2 | 2 | 2 | 2 | 2.64 | NA | 4.7 | 3870 | NA | 5095425 | 19 | 96813075 | Huh *et al.* [173] |
| *Deinonychus antirrhopus* | 122.46-112.03 | 2 | 2 | 2 | 2 | NA | NA | 3 | 440 | 96.7 | NA | NA | NA | Grellet-Tinner and Makovicky [12], Benson *et al.* [52] |
| Dromaeosaurid_type (TMP_2007) | 77.52 | 2 | 2 | 2 | 2 | 2.08 | NA | 3.8 | 775 | NA | 229500 | 12 | 2754000 | Zelenitsky and Therrien [174], Vila *et al.* [175] |
| *Troodon formosus* | 83.6-66 | 2 | 2 | 2 | 2 | 2.06 | NA | 4.8 | 850 | 47.38 | 315936 | 24 | 7582464 | Varricchio *et al.* [11], Benson *et al.* [52], Varricchio *et al.* [65] |
| *Byronosaurus jaffei* | 83.6-72.1 | 2 | 2 | 2 | 2 | NA | NA | NA | 450 | 22.5 | NA | NA | NA | Moore and Varricchio [5], Varricchio and Barta [23], Grellet-Tinner [176] |
| *Almas ukhaa* | 83.6-72.1 | 2 | 2 | 2 | 2 | NA | NA | 8 | 400 | 0.54 | NA | NA | NA | Pei *et al.* [177] |
| **Fossil birds** | | | | | | | | | | | | | | |
| *Avimaia schweitzerae* | 125-113 | 2 | 2 | 2 | 2 | NA | NA | NA | 300 | 0.156 | NA | NA | NA | Bailleul *et al.* [19], Alexander [56] |
| Enantiornithine(ZPAL_MgOv_II7) | 100.5-66 | 2 | 2 | 2 | 2 | 2.19 | NA | 2.8 | 250 | 1.16 | 36557 | 4 | 146228 | Alexander [56], David and Daniel [57] |
| Enantiornithine(IGM_100_2010) | 100.5-66 | 2 | 2 | 2 | 2 | 2.13 | NA | NA | 184 | NA | 12047 | NA | NA | Balanoff *et al.* [178], Mikhailov [179] |
| *Gobipipus reshetovi* | 83.6-72.1 | 2 | 2 | 2 | 2 | 1.19 | NA | NA | 150 | NA | 9380 | NA | NA | Kurochkin *et al.* [18] |
| *Neuquenornis volans* | 86.3-83.6 | 2 | 2 | 2 | 2 | 1.60 | NA | NA | 180 | 0.645 | 16970 | 8 | 135760 | Fernández *et al.* [16], Alexander [56], Chiappe and Calvo [58] |
| *Gobipteryx minuta* | 72.1-66 | 2 | 2 | 2 | 2 | NA | NA | 2.5 | 250 | 0.4 | NA | NA | NA | Elzanowski [17], Mikhailov [180], Elzanowski [181] |
| Enantiornithine_IVPPV14238 | 121 | 2 | NA | NA | NA | 1.75 | NA | NA | NA | NA | 7140 | NA | NA | Zhou and Zhang [21] |
| Enantiornithine_Romania | 72.1-66 | 2 | 2 | 2 | 2 | 1.60 | 0.85 | 2.8 | 300 | NA | 12750 | NA | NA | Dyke *et al.* [22] |
| Phoenicopterid_indet | 18.3 | 2 | 2 | 2 | 2 | 1.50 | NA | 4.3 | 470 | NA | 20655 | 5 | 103275 | Grellet-Tinner *et al.* [24] |
| Palaeognathae_Colorado | 55.4-50.3 | 2 | 2 | 2 | 2 | 1.33 | NA | 2.8 | 1350 | NA | 381831 | NA | NA | Hirsch *et al.* [182] |
| *Lithornis* | 56.0-41.2 | 2 | 2 | 2 | 2 | 1.44 | NA | 1.45 | 460 | 0.9 | 67129 | NA | NA | Leonard *et al.* [183], Grellet-Tinner and Dyke [184] |
| Tinamidae_indet | 0.126-0.01 | 2 | 2 | 2 | 2 | 1.54 | NA | 1.86 | 910 | NA | 78957 | NA | NA | Batista *et al.* [185] |
| *Aepyornis* | 0.5-0.2 | 2 | 2 | 2 | 2 | 1.41 | NA | 8.6 | 3590 | 158.75 | 8060774 | NA | NA | Balanoff and Rowe [25], Huynen *et al.* [59], Silyn-Roberts and Sharp [186] |
| *Gastornis* | 59.2-47.8 | 2 | 2 | 2 | 2 | 1.48 | NA | NA | 2000 | 156.4 | 1307232 | NA | NA | Angst *et al.* [187] |
| *Euryapteryx curtus* | 0.0042 | 2 | 2 | 2 | 2 | 1.25 | NA | 4.5 | 900 | 70.5 | 580629 | NA | NA | Alexander [56], Gill [60], Gill [188] |
| *Dinornis robustus* | 0.075-0.050 | 2 | 2 | 2 | 2 | 1.35 | NA | NA | 1230 | 128 | 3878122 | NA | NA | Huynen *et al.* [59], Gill [60], Gill [188] |
| *Pachyornis elephantopus* | 0.075-0.050 | 2 | 2 | 2 | 2 | 1.43 | NA | NA | 1675 | 137 | 2877351 | NA | NA | Alexander [56], Gill [60], Gill [188] |
| **Extant birds** | | | | | | | | | | | | | | |
| *Struthio camelus* | 0 | 2 | 2 | 2 | 2 | 1.21 | 0.91 | 8.6 | 1760 | 111 | 1382833 | 11 | 15211163 | Dunning Jr [48], Schönwetter [189], Hauber [190] |
| *Rhea americana* | 0 | 2 | 2 | 2 | 2 | 1.47 | NA | 6 | 1090 | 23 | 475267 | 26 | 12356942 | Dunning Jr [48], Wang and Zhang [84], Schönwetter [189], Hauber [190] |
| *Apteryx australis* | 0 | 2 | 2 | 2 | 2 | 1.65 | 0.84 | 4 | 384 | 2.33 | 400266 | 2 | 800532 | Dunning Jr [48], Hauber [190], Vieco-Galvez *et al.* [191], McLennan [192] |
| *Apteryx owenii* | 0 | 2 | 2 | 2 | 2 | 1.57 | NA | 4.5 | 440 | 1.25 | 400266 | 1.5 | 600399 | Vieco-Galvez *et al.* [191], Sales [193] |
| *Dromaius novaehollandiae* | 0 | 2 | 2 | 2 | 2 | 1.67 | 0.87 | 8.5 | 1110 | 34.2 | 619650 | 16 | 9914400 | Dunning Jr [48], Wang and Zhang [84], Hauber [190] |
| *Casuarius casuarius* | 0 | 2 | 2 | 2 | 2 | 1.46 | 0.95 | 3.8 | 1180 | 44 | 639782 | 4 | 2559128 | Dunning Jr [48], Wang and Zhang [84], Hauber [190] |
| *Emeus crassus* | 0 | 2 | 2 | 2 | 2 | 1.41 | NA | NA | 1010 | NA | 1998570 | NA | NA | Grellet-Tinner [194] |
| *Eudromia elegans* | 0 | 2 | 2 | 2 | 2 | 1.36 | 0.84 | 2 | 200 | 0.704 | 41113 | 6 | 246676 | Dunning Jr [48], Hauber [190], Grellet-Tinner [194] |
| *Nothoprocta perdicaria* | 0 | 2 | 2 | 2 | 2 | 1.44 | NA | 1.9 | 170 | 0.458 | 39642 | 6 | 212000 | Dunning Jr [48], Schönwetter [189], Arias *et al.* [195], Jetz *et al.* [196] |
| *Tinamus major* | 0 | 2 | 2 | 2 | 2 | 1.21 | NA | 2.4 | 219 | 1.03 | 68152 | 4 | 272608 | Dunning Jr [48], Igic *et al.* [83], Hauber [190] |
| *Leipoa ocellata* | 0 | 2 | 2 | 2 | 2 | 1.56 | NA | NA | 233 | 1.917 | 162068 | 16 | 2593088 | Dunning Jr [48], Schönwetter [189], Booth [197], Brickhill [198] |
| *Crax rubra* | 0 | 2 | 2 | 2 | 2 | 1.41 | NA | 4.4 | 680 | 4.13 | 200071 | 2 | 400142 | Dunning Jr [48], Schönwetter [189], Héctor and Roberto [199], Martínez-Morales *et al.* [200] |
| *Colinus virginianus* | 0 | 2 | 2 | 2 | 2 | 1.26 | NA | NA | 301 | 0.178 | 8872 | 14 | 124208 | Dunning Jr [48], Schönwetter [189], Hauber [190], Piccirillo and Orlando [201] |
| *Chauna torquata* | 0 | 2 | 2 | 2 | 2 | 1.48 | NA | 5 | 371 | 4.4 | 147545 | 4 | 590180 | Moore and Varricchio [5], Tyler [81], Schönwetter [189], Fox *et al.* [202] |
| *Dendrocygna autumnalis* | 0 | 2 | 2 | 2 | 2 | 1.33 | NA | NA | 305 | 0.756 | 40337 | 14 | 564718 | Dunning Jr [48], Tyler [81], Hauber [190] |
| *Oxyura jamaicensis* | 0 | 2 | 2 | 2 | 2 | 1.35 | NA | NA | 298 | 0.609 | 66908 | 10 | 669080 | Dunning Jr [48], Tyler [81], Hauber [190] |
| *Anser anser* | 0 | 2 | 2 | 2 | 2 | 1.48 | 0.92 | 5.2 | 480 | 3.31 | 147545 | 5 | 737725 | Dunning Jr [48], Schönwetter [189], Bingol *et al.* [203], Hirschenhauser *et al.* [204] |
| *Ortalis vetula* | 0 | 2 | 2 | 2 | 2 | 1.40 | NA | NA | 410 | 0.563 | 53079 | 3 | 159237 | Dunning Jr [48], Hauber [190], Zelenitsky *et al.* [205] |
| *Anas platyrhynchos* | 0 | 2 | 2 | 2 | 2 | 1.38 | 0.90 | 3.7 | 286 | 1.14 | 49171 | 11 | 540881 | Tyler [81], Schönwetter [189], Hauber [190] |
| *Caprimulgus europaeus* | 0 | 2 | 2 | 2 | 2 | 1.42 | NA | NA | 108 | 0.067 | 7917 | 2 | 15834 | Dunning Jr [48], Maurer *et al.* [85], Schönwetter [189], Berry [206] |
| *Chordeiles acutipennis* | 0 | 2 | 2 | 2 | 2 | 1.40 | NA | NA | 126 | 0.0485 | 5692 | 2 | 11384 | Dunning Jr [48], Schönwetter [189], Grant [207], Cestari [208] |
| *Gavia immer* | 0 | 2 | 2 | 2 | 2 | 1.58 | NA | NA | 473 | 4.98 | 134219 | 2 | 268438 | Dunning Jr [48], Tyler [82], Hauber [190] |
| *Cuculus canorus* | 0 | 2 | 2 | 2 | 2 | 1.32 | NA | 2 | 84 | 0.112 | 3096 | 2 | 6192 | Igic *et al.* [83], Schönwetter [189], Hauber [190], Payevsky [209] |
| *Rallus aquaticus* | 0 | 2 | 2 | 2 | 2 | 1.38 | NA | NA | 153 | 0.112 | 12346 | 7 | 86422 | Dunning Jr [48], Maurer *et al.* [85], David *et al.* [210] |
| *Syrrhaptes paradoxus* | 0 | 2 | 2 | 2 | 2 | 1.44 | NA | 3.9 | 186 | 0.257 | 18863 | 3 | 56589 | Dunning Jr [48], Schönwetter [189], Hauber [190], Mikhailov [211] |
| *Aramus guarauna* | 0 | 2 | 2 | 2 | 2 | 1.36 | NA | 3 | 236 | 1.08 | 59242 | 6 | 355452 | Dunning Jr [48], Hauber [190], Mikhailov [211] |
| *Phoenicopterus ruber* | 0 | 2 | 2 | 2 | 2 | 1.65 | 0.89 | NA | 560 | 3.07 | 139619 | 1 | 139619 | Dunning Jr [48], Hirsch [79], Schönwetter [189], Hauber [190] |
| *Phaethon aethereus* | 0 | 2 | 2 | 2 | 2 | 1.50 | NA | NA | 220 | 0.75 | 48960 | 1 | 48960 | Dunning Jr [48], Tyler [82], Hauber [190] |
| *Burhinus oedicnemus* | 0 | 2 | 2 | 2 | 2 | 1.31 | NA | NA | 237 | 0.459 | 37406 | 3 | 112218 | Dunning Jr [48], Ar *et al.* [86], Schönwetter [189], Hauber [190] |
| *Charadrius alexandrinus* | 0 | 2 | 2 | 2 | 2 | 1.35 | NA | NA | 140 | 0.0423 | 8363 | 4 | 33452 | Dunning Jr [48], Maurer *et al.* [85], Hauber [190] |
| *Alectoris graeca* | 0 | 2 | 2 | 2 | 2 | 1.18 | 0.98 | NA | 213 | 0.615 | 23582 | 11 | 259406 | Stein *et al.* [8], Hauber [190], Kirikçı *et al.* [212] |
| *Aquila rapax* | 0 | 2 | 2 | 2 | 2 | 1.30 | NA | NA | 427 | 2.25 | 107401 | 1.5 | 161102 | Stein *et al.* [8], Schönwetter [189], Tyler [213], Hustler and Howells [214] |
| *Ardea cinerea* | 0 | 2 | 2 | 2 | 2 | 1.42 | NA | NA | 215 | 1.443 | 57522 | 3.5 | 201328 | Stein *et al.* [8], Hauber [190] |
| *Athene noctua* | 0 | 2 | 2 | 2 | 2 | 1.19 | NA | NA | 166 | 0.169 | 14552 | 3.95 | 57479 | Stein *et al.* [8], Schönwetter [189], Exo [215] |
| *Bubo bubo* | 0 | 2 | 2 | 2 | 2 | 1.21 | NA | NA | 302 | 2.686 | 54736 | 2.78 | 152165 | Andreychev *et al.* [216] Stein *et al.* [8], Schönwetter [189], Tyler [213] |
| *Buteo rufinus* | 0 | 2 | 2 | 2 | 2 | 1.28 | NA | NA | 330 | 1.1745 | 67595 | 2.7 | 182508 | Stein *et al.* [8], Schönwetter [189], Iezekiel *et al.* [217] |
| *Carduelis carduelis* | 0 | 2 | 2 | 2 | 2 | 1.31 | NA | NA | 62 | 0.016 | 1465 | 5 | 7326 | Stein *et al.* [8], Hauber [190] |
| *Cercotrichas galactotes* | 0 | 2 | 2 | 2 | 2 | 1.37 | NA | NA | 71 | 0.0203 | 2712 | 3.43 | 9301 | Stein *et al.* [8], Adamou *et al.* [218], Adamou *et al.* [219] |
| *Chloris chloris* | 0 | 2 | 2 | 2 | 2 | 1.40 | NA | NA | 60 | 0.026 | 2074 | 4 | 8297 | Stein *et al.* [8], Dunning Jr [48], Hauber [190] |
| *Chroicocephalus ridibundus* | 0 | 2 | 2 | 2 | 2 | 1.42 | NA | NA | 206 | 0.284 | 35865 | 4 | 143461 | Stein *et al.* [8], Hauber [190] |
| *Ciconia ciconia* | 0 | 2 | 2 | 2 | 2 | 1.38 | NA | NA | 447 | 3.448 | 99291 | 3.5 | 347518 | Stein *et al.* [8], Hauber [190] |
| *Cinnyris osea* | 0 | 2 | 2 | 2 | 2 | 1.38 | NA | NA | 46 | 0.0072 | 985 | 2 | 1970 | Stein *et al.* [8], Schönwetter [189], Goldstein and Yom-Tov [220] |
| *Corvus corone* | 0 | 2 | 2 | 2 | 2 | 1.52 | NA | 2.1 | 157 | 0.57 | 18880 | 4.34 | 81940 | Stein *et al.* [8], Dolenec [221] |
| *Egretta garzetta* | 0 | 2 | 2 | 2 | 2 | 1.39 | NA | NA | 194 | 0.312 | 29082 | 3.5 | 101787 | Stein *et al.* [8], Prashant *et al.* [222] |
| *Falco peregrinus* | 0 | 2 | 2 | 2 | 2 | 1.26 | NA | 3.5 | 293 | 0.783 | 45629 | 4 | 182516 | Tyler [213], Burnham *et al.* [223] |
| *Falco tinnunculus* | 0 | 2 | 2 | 2 | 2 | 1.24 | NA | 3.5 | 199 | 0.184 | 24733 | 4.2 | 103880 | Tyler [213] Stein *et al.* [8], Jetz *et al.* [196] Schönwetter [189] |
| *Galerida cristata* | 0 | 2 | 2 | 2 | 2 | 1.29 | NA | NA | 85 | 0.0428 | 3243 | 4.5 | 14592 | Stein *et al.* [8], Hauber [190] |
| *Gallinula chloropus* | 0 | 2 | 2 | 2 | 2 | 1.41 | NA | NA | 204 | 0.3435 | 20534 | 7.25 | 148874 | Stein *et al.* [8], [189], Samraoui *et al.* [224] |
| *Gallus gallus* | 0 | 2 | 2 | 2 | 2 | 1.43 | 0.89 | 3.4 | 320 | 0.904 | 23019 | 7.86 | 180928 | Stein *et al.* [8], Apuno *et al.* [225] |
| *Geronticus eremita* | 0 | 2 | 2 | 2 | 2 | 1.43 | NA | NA | 351 | 1.202 | 63169 | 2.8 | 176873 | Stein *et al.* [8], Schönwetter [189], Jetz *et al.* [196] |
| *Glareola pratincola* | 0 | 2 | 2 | 2 | 2 | 1.33 | NA | 1 | 134 | 0.0849 | 9400 | 2 | 18801 | Schönwetter [189], Stein *et al.* [8], Bensaci *et al.* [226] |
| *Gyps fulvus* | 0 | 2 | 2 | 2 | 2 | 1.28 | NA | NA | 601 | 7.436 | 233953 | 1 | 233953 | Stein *et al.* [8], Hauber [190] |
| *Himantopus himantopus* | 0 | 2 | 2 | 2 | 2 | 1.39 | NA | NA | 165 | 0.161 | 20842 | 3.65 | 76072 | Stein *et al.* [8], Mamedova and Chaplygina [227] |
| *Lanius nubicus* | 0 | 2 | 2 | 2 | 2 | 1.25 | NA | NA | 85 | 0.0197 | 2611 | 4.6 | 12012 | Stein *et al.* [8], Ar *et al.* [86], Harris and Franklin [228] |
| *Melopsittacus undulatus* | 0 | 2 | 2 | 2 | 2 | 1.20 | 0.69 | 1.8 | 98 | 0.029 | 2066 | 5 | 10328 | Stein *et al.* [8], Hauber [190] |
| *Muscicapa striata* | 0 | 2 | 2 | 2 | 2 | 1.29 | NA | NA | 68 | 0.0159 | 1799 | 5 | 8996 | Stein *et al.* [8], Hauber [190] |
| *Nycticorax nycticorax* | 0 | 2 | 2 | 2 | 2 | 1.41 | NA | NA | 182 | 0.81 | 36306 | 4.5 | 163376 | Stein *et al.* [8], Hauber [190] |
| *Passer domesticus* | 0 | 2 | 2 | 2 | 2 | 1.38 | NA | 1.2 | 91 | 0.0277 | 2872 | 4.5 | 12925 | Stein *et al.* [8], Hauber [190] |
| *Passer moabiticus* | 0 | 2 | 2 | 2 | 2 | 1.37 | NA | 1.2 | 78 | 0.0165 | 1919 | 4.1 | 7869 | Stein *et al.* [8], Yom-Tov and Ar [229], Jamadi and Darvishi [230] |
| *Prinia gracilis* | 0 | 2 | 2 | 2 | 2 | 1.42 | NA | NA | 61 | 0.0071 | 1248 | 4 | 4994 | Stein *et al.* [8], Hauber [190] |
| *Pycnonotus capensis* | 0 | 2 | 2 | 2 | 2 | NA | NA | NA | 74 | 0.0386 | 2839 | 2.4 | 6812 | Stein *et al.* [8], Jetz *et al.* [196] |
| *Sterna hirundo* | 0 | 2 | 2 | 2 | 2 | 1.40 | 0.74 | NA | 137 | 0.1295 | 19278 | 2.5 | 48195 | Stein *et al.* [8], Hauber [190], Sander [231] |
| *Streptopelia decaocto* | 0 | 2 | 2 | 2 | 2 | 1.32 | NA | NA | 117 | 0.149 | 8731 | 1.89 | 16502 | Stein *et al.* [8], Schönwetter [189], Robertson [232] |
| *Streptopelia risoria* | 0 | 2 | 2 | 2 | 2 | 1.28 | 0.65 | 1 | 111 | 0.17 | 7473 | 1.5 | 11210 | Stein *et al.* [8], Cate and Hilbers [233], Hubbard [234], Valitutto *et al.* [235] |
| *Streptopelia turtur* | 0 | 2 | 2 | 2 | 2 | 1.35 | NA | NA | 122 | 0.132 | 7728 | 1.91 | 14761 | Stein *et al.* [8], Schönwetter [189], Alam *et al.* [236] |
| *Strix aluco* | 0 | 2 | 2 | 2 | 2 | 1.22 | NA | NA | 238 | 0.475 | 36469 | 3.2 | 116702 | Stein *et al.* [8], Schönwetter [189], Jetz *et al.* [196] |
| *Turdus merula* | 0 | 2 | 2 | 2 | 2 | 1.42 | 0.73 | 3 | 107 | 0.1032 | 6702 | 2.96 | 19839 | Stein *et al.* [8], Vogrin [237], Zeraoula *et al.* [238] |
| *Tyto alba* | 0 | 2 | 2 | 2 | 2 | 1.33 | NA | NA | 275 | 0.35 | 24437 | 6 | 146623 | Stein *et al.* [8], Dunning Jr [48], Hauber [190], Salim *et al.* [239] |
| **Lepidosaurs** | | | | | | | | | | | | | | |
| Auguimorph_Thailand | 129-121 | 2 | 2 | 2 | 2 | 1.64 | 0.89 | NA | 331 | NA | 1044 | NA | NA | Fernandez *et al.* [43] |
| *Varanus komodoensis* | 0 | 0 | 0 | 0 | 0 | 1.56 | NA | NA | NA | 37.14 | 126985 | 17.5 | 2222242 | Legendre *et al.* [76], Sunter [240] |
| *Varanus indicus* | 0 | 0 | 0 | 0 | 0 | 1.33 | 0.07 | NA | 40 | 1.287 | 68533 | 7 | 479729 | Legendre *et al.* [76] |
| *Agama planiceps* | 0 | 0 | 0 | 0 | 0 | 1.44 | 0.46 | NA | 43 | 0.0493 | 496 | 6 | 2978 | Legendre *et al.* [76], Heideman [241] |
| *Anolis carolinensis* | 0 | 0 | 0 | 0 | 0 | 1.75 | 0.29 | NA | 50 | 0.00225 | 182 | 1 | 182 | Legendre *et al.* [76], Hamlett [242] |
| *Aspidoscelis sexlineatus* | 0 | 0 | 0 | 0 | 0 | 1.73 | 0.07 | NA | 9 | 0.007191 | 739 | 3.7 | 2735 | Legendre *et al.* [76], Meiri *et al.* [243] |
| *Bothrops atrox* | 0 | 0 | 0 | 0 | 0 | 1.23 | 0.13 | NA | 6 | 1.8034 | 10623 | 11 | 116852 | Legendre *et al.* [76], Silva *et al.* [244] |
| *Bronchocela marmorata* | 0 | 0 | 0 | 0 | 0 | 3.88 | 0.05 | NA | 9 | 0.0668 | 2570 | 2 | 5141 | Legendre *et al.* [76], Meiri *et al.* [243] |
| *Calloselasma rhodostoma* | 0 | 0 | 0 | 0 | 0 | 1.33 | 0.11 | NA | 6 | 0.264 | 8567 | 21.5 | 184182 | Legendre *et al.* [76], Hill Iii *et al.* [245] |
| *Chamaeleo senegalensis* | 0 | 0 | 0 | 0 | 0 | 1.78 | 0.02 | NA | 9 | 0.0311 | 627 | 52.5 | 32893 | Legendre *et al.* [76], Meiri *et al.* [243] |
| *Coluber constrictor* | 0 | 0 | 0 | 0 | 0 | 1.40 | 0.09 | NA | 6 | 0.121 | 4651 | 15 | 69763 | Legendre *et al.* [76], Rosen [246] |
| *Crotalus viridis* | 0 | 0 | 0 | 0 | 0 | 2.13 | 0.15 | NA | 3 | 0.21171 | 2185 | 5.5 | 12018 | Legendre *et al.* [76], Diller and Wallace [247] |
| *Cryptoblepharus poecilopleurus* | 0 | 0 | 0 | 0 | 0 | 1.39 | 0.10 | NA | 7 | 0.0026 | 1739 | 1.1 | 1913 | Legendre *et al.* [76], Goldberg and Kraus [248] |
| *Diadophis punctatus* | 0 | 0 | 0 | 0 | 0 | 3.71 | 0.24 | NA | 11 | 0.019 | 765 | 3.55 | 2717 | Legendre *et al.* [76], Clark *et al.* [249] |
| *Elgaria multicarinata* | 0 | 0 | 0 | 0 | 0 | 1.58 | 0.17 | NA | 9 | 0.0317 | 874 | 10.9 | 9525 | Legendre *et al.* [76], Meiri *et al.* [243] |
| *Eublepharis macularius* | 0 | 1 | 1 | 1 | 0 | 1.86 | 0.51 | NA | 36 | 0.05965 | 2873 | 2 | 5747 | Schleich and Kästle [78], Choi *et al.* [127], Rhen *et al.* [250], LaDage *et al.* [251] |
| *Eunectes notaeus* | 0 | 0 | 0 | 0 | 0 | 2.43 | 0.07 | NA | 41 | 5.32279 | 61254 | 12 | 735049 | Legendre *et al.* [76], O’Shea and Halliday [252] |
| *Gekko gecko* | 0 | 2 | 2 | 2 | 2 | 1.00 | 0.89 | NA | 280 | 0.06315 | 5229 | 2 | 10459 | Legendre *et al.* [76], Meiri *et al.* [243] |
| *Hemidactylus bouvieri* | 0 | 2 | 2 | 2 | 2 | 1.00 | NA | NA | 55 | 0.0028 | 313 | 1.5 | 470 | Legendre *et al.* [76] |
| *Heterodon platirhinos* | 0 | 0 | 0 | 0 | 0 | 2.14 | 0.16 | NA | 13 | 0.28 | 6118 | 28.3 | 173148 | Legendre *et al.* [76], Peet-Paré and Blouin-Demers [253] |
| *Iguana iguana* | 0 | 0 | 0 | 0 | 0 | 1.38 | 0.15 | NA | 18 | 1.53 | 16221 | 29.7 | 481761 | Legendre *et al.* [76], Alvarado *et al.* [254] |
| *Kentropyx altamazonica* | 0 | 0 | 0 | 0 | 0 | 1.55 | 0.06 | NA | 16 | 0.0235 | 1078 | 5.45 | 5876 | Legendre *et al.* [76], Werneck *et al.* [255] |
| *Kinyongia tavetana* | 0 | 0 | 0 | 0 | 0 | 1.86 | 0.06 | NA | 3 | 0.0213 | 309 | 11 | 3399 | Legendre *et al.* [76], Meiri *et al.* [243] |
| *Lacerta viridis* | 0 | 0 | 0 | 0 | 0 | 1.64 | 0.13 | NA | 6 | 0.039 | 1044 | 12 | 12530 | Legendre *et al.* [76], Braña *et al.* [256] |
| *Lampropeltis getula* | 0 | 0 | 0 | 0 | 0 | 2.09 | 0.12 | NA | 12 | 0.188 | 13785 | 13.5 | 186099 | Legendre *et al.* [76], O’Shea and Halliday [252] |
| *Masticophis bilineatus* | 0 | 0 | 0 | 0 | 0 | 2.34 | 0.20 | NA | 45 | 0.4509 | 8278 | 6.8 | 56289 | Legendre *et al.* [76], Goldberg [257] |
| *Masticophis flagellum* | 0 | 0 | 0 | 0 | 0 | 1.67 | 0.15 | NA | 35 | 0.173 | 11068 | 7.2 | 79693 | Legendre *et al.* [76], Goldberg [258] |
| *Mediodactylus kotschyi* | 0 | 2 | 2 | 2 | 2 | 1.19 | NA | NA | 39 | 0.002 | 210 | 2 | 420 | Legendre *et al.* [76], Mollov [259] |
| *Micrurus mipartitus* | 0 | 0 | 0 | 0 | 0 | 1.67 | 0.06 | NA | 5 | 0.3387 | 2114 | 18 | 38058 | Legendre *et al.* [76], Solórzano and Cerdas [260] |
| *Naja melanoleuca* | 0 | 0 | 0 | 0 | 0 | 2.09 | 0.10 | NA | 21 | 2.7561 | 23412 | 15 | 351182 | Legendre *et al.* [76], Haagner and Carpenter [261] |
| *Pantherophis guttatus* | 0 | 0 | 0 | 0 | 0 | 1.93 | 0.11 | NA | 10 | 0.695 | 5751 | 12.6 | 72459 | Legendre *et al.* [76], Bird *et al.* [262] |
| *Phelsuma madagascariensis* | 0 | 2 | 2 | 2 | 2 | 1.74 | NA | NA | 130 | 0.02068 | 2067 | 1.8 | 3720 | Legendre *et al.* [76], Demeter [263] |
| *Phrynosoma cornutum* | 0 | 0 | 0 | 0 | 0 | 1.56 | 0.17 | NA | 6 | 0.038 | 540 | 26.49 | 14313 | Legendre *et al.* [76], Ballinger [264] |
| *Pituophis catenifer* | 0 | 0 | 0 | 0 | 0 | 1.84 | 0.10 | NA | 22 | 0.41368 | 24085 | 7.2 | 173414 | Legendre *et al.* [76], Iverson *et al.* [265] |
| *Ptyodactylus hasselquistii* | 0 | 2 | 2 | 2 | 2 | 1.00 | NA | NA | 150 | 0.00934375 | 1011 | 2 | 2022 | Legendre *et al.* [76], Werner [266] |
| *Python molurus* | 0 | 0 | 0 | 0 | 0 | 1.66 | 0.24 | NA | 55 | 12.37 | 155027 | 35 | 5425933 | Legendre *et al.* [76], O’Shea and Halliday [252] |
| *Sceloporus magister* | 0 | 0 | 0 | 0 | 0 | 1.50 | 0.09 | NA | 6 | 0.04358 | 711 | 12.3 | 8747 | Legendre *et al.* [76], Vitt and Ohmart [267] |
| *Sceloporus undulatus* | 0 | 0 | 0 | 0 | 0 | 1.74 | 0.12 | NA | 5 | 0.01125 | 327 | 5.55 | 1812 | Legendre *et al.* [76], Ballinger *et al.* [268] |
| *Sphenodon punctatus* | 0 | 1 | 1 | 1 | NA | 1.30 | 0.52 | NA | 30 | 0.608230769 | 11792 | 7.6 | 89616 | Packard *et al.* [126], Cree *et al.* [269], Nelson *et al.* [270] |
| *Tarentola delalandii* | 0 | 2 | 2 | 2 | 2 | 1.22 | NA | NA | 34 | 0.00603 | 556 | 1.5 | 834 | Legendre *et al.* [76], Fuentes-Fernandez *et al.* [271], Marques *et al.* [272] |
| *Tarentola gigas* | 0 | 2 | 2 | 2 | 2 | 1.00 | 0.83 | NA | 33 | 0.093 | 3438 | 2 | 6877 | Legendre *et al.* [76], Marques *et al.* [272] |
| *Tarentola mauritanica* | 0 | 2 | 2 | 2 | 2 | 1.25 | 0.86 | NA | 41 | 0.0073 | 628 | 1.5 | 942 | Legendre *et al.* [76], Marques *et al.* [272] |
| *Trioceros jacksonii* | 0 | 0 | 0 | 0 | 0 | 1.69 | NA | NA | 16 | 0.03746 | 541 | 16.95 | 9174 | Legendre *et al.* [76], Meiri *et al.* [243] |
| *Tupinambis teguixin* | 0 | 0 | 0 | 0 | 0 | 1.74 | 0.16 | NA | 56 | 2.212 | 18250 | 14 | 255505 | Legendre *et al.* [76], Herrera and Robinson [273] |
| *Urosaurus ornatus* | 0 | 0 | 0 | 0 | 0 | 1.51 | 0.41 | NA | 22 | 0.00335 | 196 | 5.38 | 1057 | Legendre *et al.* [76], Martin [274] |
| *Uta stansburiana* | 0 | 0 | 0 | 0 | 0 | 1.70 | 0.22 | NA | 14 | 0.003 | 281 | 3.5 | 982 | Legendre *et al.* [76], Hoddenbach and Turner [275] |
| **turtles** | | | | | | | | | | | | | | |
| *Caretta caretta* | 0 | 0 | 1 | 0 | 0 | 1.00 | 0.55 | 2 | 80 | 200 | 50965 | 132 | 6727381 | Schleich and Kästle [78], Elgar and Heaphy [144], Zhou [276] |
| *Chelonoidis carbonarius* | 0 | 2 | 2 | 2 | 2 | 1.12 | 0.86 | 1.23 | 430 | 5.9 | 27368 | 6.5 | 177891 | Schleich and Kästle [78], Williams *et al.* [277] |
| *Chelonoidis denticulatus* | 0 | 2 | 2 | 2 | 2 | 1.00 | 0.94 | 1.1 | 535 | 6.4 | 87114 | 5 | 435569 | Schleich and Kästle [78], de Miranda [278] |
| *Chelonoidis niger* | 0 | 2 | 0 | 2 | 2 | 1.09 | 0.46 | NA | 155 | 250 | 95106 | 12 | 1141272 | Schleich and Kästle [78], Zhou and Zhou [279] |
| *Chelus fimbriata* | 0 | 2 | 2 | 2 | 2 | 1.35 | NA | NA | 280 | 13.6 | 22097 | 10 | 220974 | Schleich and Kästle [78], Pritchard [280] |
| *Chelydra serpentina* | 0 | 1 | 1 | 2 | 1 | 1.01 | 0.58 | 1.27 | 110 | 6 | 10583 | 47 | 497394 | Stein *et al.* [8], Iverson *et al.* [146] |
| *Cuora amboinensis* | 0 | 2 | 2 | 2 | 2 | 1.34 | 0.73 | 2 | 290 | 1.2 | 23073 | 3 | 69218 | Schleich and Kästle [78], Andersen *et al.* [281] |
| *Kinosternon subrubrum* | 0 | 2 | 2 | 2 | 2 | 1.88 | 0.65 | 1.1 | 270 | 0.1146 | 4024 | 3.5 | 14085 | Schleich and Kästle [78], Congdon *et al.* [282] |
| *Lepidochelys kempii* | 0 | 0 | 0 | 0 | 0 | 1.00 | 0.33 | 0.5 | 40 | 42.5 | 31059 | 104 | 3230173 | Stein *et al.* [8], Márquez [283], Pritchard and Mortimer [284] |
| *Mauremys caspica* | 0 | 2 | 2 | 2 | 2 | 1.66 | 0.75 | 1.4 | 150 | 0.338 | 13591 | 10.5 | 142708 | Schleich and Kästle [78], Pages *et al.* [285] |
| *Stigmochelys pardalis* | 0 | 2 | 1 | 2 | 2 | 1.00 | 0.64 | 1 | 210 | 15 | 5964 | 23 | 137175 | Schleich and Kästle [78], Holt *et al.* [286] |
| *Terrapene carolina_triunguis* | 0 | 1 | 1 | 2 | 1 | 1.70 | 0.55 | 1.1 | 130 | 0.44 | 7413 | 5.5 | 40772 | Schleich and Kästle [78], Costanzo and Claussen [287] |
| *Testudo graeca* | 0 | 2 | 2 | 2 | 2 | 1.39 | 0.77 | 1.8 | 165 | 1.225 | 16022 | 5 | 80109 | Schleich and Kästle [78], Lambert [288] |
| *Testudo hermanni* | 0 | 2 | 2 | 2 | 2 | 1.28 | 0.74 | 1.5 | 200 | 1.0875 | 12511 | 8 | 100090 | Schleich and Kästle [78], Willemsen and Hailey [289] |
| *Testudo marginata* | 0 | 2 | 0 | 2 | 2 | 1.10 | 0.29 | 1.1 | 90 | 4.6 | 15563 | 6.5 | 101158 | Schleich and Kästle [78], Gaio *et al.* [290] |
| *Trionyx triunguis* | 0 | 2 | 2 | 2 | 2 | 1.00 | NA | 0.5 | 175 | 90 | 17157 | 55 | 943651 | Schleich and Kästle [78], Burghardt *et al.* [291] |
| *Testudoflexoolithus bathonicae* | 168.3-166.1 | 1 | 1 | 0 | 1 | 1.85 | 0.65 | 1 | 225 | NA | 17003 | NA | NA | Lawver and Jackson [41], Lawver and Jackson [292] |
| *Meiolania platyceps* | 0.774-0.0117 | 2 | 2 | 2 | 2 | 1.00 | NA | 1.2 | 800 | NA | 81991 | NA | NA | Lawver and Jackson [41] |
| Nanhsiungchelyid_Henan | 93.9-72.1 | 2 | 2 | 2 | 2 | 1.00 | NA | 8.2 | 1670 | NA | 69456 | 36 | 2500412 | Ke *et al.* [38], Xu *et al.* [293] |
| *Adocus_*sp | 75 | 2 | 2 | 2 | 2 | 1.06 | 0.80 | 3 | 770 | NA | 40194 | 26 | 1045054 | Zelenitsky *et al.* [294] |
| *Desmatochelys padillai* | 129.4-113.0 | 2 | 2 | 2 | 2 | 1.00 | 0.88 | 1.2 | 803 | NA | 27612 | 48 | 1325359 | Cadena *et al.* [39] |
| *Bairdemys venezuelensis* | 23.03-5.33 | 2 | 2 | 2 | 2 | 1.30 | NA | 2 | 648 | NA | 56022 | NA | NA | Winkler and SÁnchez-Villagra [295] |

### Supplementary Table 10. Sample size for each major clade in this study.

| Reptile clades | Number (extant) | Number (fossils) | Number (total) |
| --- | --- | --- | --- |
| Non-avialan Dinosauria | 0 | 37 | 37 |
| Aves (birds) | 68 | 17 | 85 |
| Pterosauria | 0 | 5 | 5 |
| Crocodylomorpha | 11 | 3 | 14 |
| Testudines | 16 | 6 | 22 |
| Lepidosauria | 45 | 1 | 46 |
| Choristodera | 0 | 1 | 1 |
| Total | 140 | 70 | 210 |

### Supplementary Table 11. Differences of eggshell type Scoring between Legendre et al. (*48*) and this study

| Taxa | new scoring | ratio scoring | References |
| --- | --- | --- | --- |
| *Massospondylus* | leathery | soft | Legendre et al. [7] |
|  | leathery | **leathery** | This study |
| *Lufengosaurus* | leathery | soft | Legendre et al. [7] |
|  | leathery | **leathery** | This study |
| *Mussaurus* | soft | soft | Legendre et al. [7] |
|  | **leathery** | soft | This study |
| *Qianlong* | **leathery** | **?** | This study |
| *Pterodaustro_guinazui* | hard | hard | Legendre et al. [7] |
|  | hard | **?** | This study |
| *Testudoflexoolithus bathonicae* | hard | hard | Legendre et al. [7] |
|  | **leathery** | **leathery** | This study |
| *Testudo graeca* | Hard | Soft | Legendre et al. [7] |
|  | Hard | **Hard** | This study |

### Supplementary Table 12. Comparison of EDS results of eggshell for *Qianlong* and *Gallus gallus domesticus*

| Taxon | *Qianlong shouhu* | | *Gallus gallus domesticus* | |
| --- | --- | --- | --- | --- |
| Elements | Weight percentage | Atom percentage | Weight percentage | Atom percentage |
| C | 12.23 | 19.84 | 15.26 | 23.82 |
| O | 50.73 | 61.78 | 51.89 | 60.81 |
| Mg | 1.16 | 0.93 | 0 | 0 |
| Ca | 35.88 | 17.45 | 32.85 | 15.37 |
| Total | 100.00 |  | 100.00 |  |

Standard samples

C CaCO_3_ 1-Jun-1999 12:00 AM

O SiO_2_ 1-Jun-1999 12:00 AM

Mg MgO 1-Jun-1999 12:00 AM

Ca Wollastonite 1-Jun-1999 12:00 AM

### Supplementary Table 13. Scanning parameters of four embryos of *Qianlong* in this study.

| Specimen number | Voltage (KV) | current (μA) | Resolution (µm) | Filter  (mm) | Proj | Exp  (ms) | Acc | Output images | Working place |
| --- | --- | --- | --- | --- | --- | --- | --- | --- | --- |
| GZPM VN004-1 | 200 | 100 | 22.49 | — | — | — | — | 3063 | YITSC |
| GZPM VN004-2 | 200 | 100 | 22.49 | — | — | — | — | 3098 | YITSC |
| GZPM VN006-1 | 200 | 150 | 35.044 | Cu:5 | 2500 | 1000 | 3 | 2742 | IVPP |
| GZPM VN006-2 | 180 | 140 | 34.573 | Cu:5 | 2500 | 1000 | 2 | 1842 | IVPP |

**Abbreviations**: **Acc**, number of images accumulated per projection; **Exp** (ms), total exposure per projection in milli-seconds; **Proj**, number of projections per complete acquisition; **IVPP**, Institute of Vertebrate Paleontology and Paleoanthropology; **YITSC**, Yinghua Inspection and Testing Shanghai Company.

### Supplementary Table 14. References used to associate egg fossils with known adult taxa in this study.

| **Genus or species** | **Higher Taxon** | **Specimen number** | **Preservation** | | **References** |
| --- | --- | --- | --- | --- | --- |
|  |  |  | **With adult skeletons** | **Embryos** |  |
| *Pterodaustro guinazui* | Pterosaur | MHIN-UNSL-GEO-V246; MIC-V246 | No | Within egg | Chiappe *et al.* [26]; Codorniú *et al.* [153] |
| *Beipiaopterus* | Pterosaur | JZMP-03-03-2 | No | Within egg | Ji *et al.* [28] |
| *Yixianopterus jingangshanensis* | Pterosaur | IVPP V13758 | No | Within egg | Wang and Zhou [29]; Unwin and Deeming [30] |
| *Kunpengopterus* | Pterosaur | ZMNH M8802/IVPP V18403 | Atop eggs (laying egg) | No | Lü *et al.* [31]; Wang *et al.* [32] |
| *Hamipterus tianshanensis* | Pterosaur | IVPP V 18941 to 18943 | Nearby eggs | Within egg | Wang *et al.* [33], [64] |
| *Lufengosaurus* | Sauropodomorpha | C2019 2A 233 | No | Nearby eggshell | Reisz *et al.* [109] |
| *Mussaurus patagonicus* | Sauropodomorpha | MPM-PV 1887 ; MPM-PV1879 | Nearby eggs | Within eggs | Bonaparte and Vince [131]; Pol *et al.* [1] |
| *Massospondylus* | Sauropodomorpha | BP/1/5347A; BP/1/6229; BP/1/5347 | No | Within eggs | Reisz *et al.* [10]; Reisz *et al.* [87] |
| *Qianlong* | Sauropodomorpha | GZPM VN004-008 | Nearby eggs | Within eggs | this study |
| *Megaloolithus patagonicus* | Titanosauria (Sauropoda) | MCF-PVPH-258 | No | No | Chiappe *et al.* [66] |
| *Megaloolithus* sp. | Titanosauria (Sauropoda) | MCF-PVPH-255 | No | No, identified by eggshell | Jackson *et al.* [67] |
| *Megaloolithus siruguei* | Titanosauria (Sauropoda) | Pinyes 18 -E02 | No | No, identified by eggshell | Vila *et al.* [156] |
| Titanosaur (Tama_Argentina) | Titanosauria (Sauropoda) | CRILAR-Pv 530/1 | No | No, identified by eggshell | Hechenleitner *et al.* [68] |
| Neosauropoda (Sanagasta_Argentina) | Titanosauria (Sauropoda) | NA | No | No, identified by eggshell | Grellet-Tinner and Fiorelli [157] |
| Lithostrotia | Lithostrotia (Sauropoda) | NSM60104403-20554450 | No | Within eggs | Grellet-Tinner *et al.* [69] |
| Titanosaur (Patagonia_Argentina) | Sauropoda | MCF-PVPH-874 | No | Within eggs | Kundrát *et al.* [158] |
| Titanosaur (Romania) | Titanosauria (Sauropoda) | TO O–01 | No | No | Grellet-Tinner *et al.* [70] |
| *Hypacrosaurus stebingeri* | Lambeosaurine (Hadrosauridae) | TMP 87.79.149; MOR 707 | No | Within eggs | Erickson *et al.* [296] |
| *Maiasaura peeblesorum* | Hadrosaurinae (Hadrosauridae) | MOR 244 (embryo) | No | Nearby eggs | Horner [71] |
| *Telmatosaurus transsylvanicus* | Hadrosauridae | __ | No | Within eggs | Grigorescu [160]; Grigorescu [72] |
| *Protoceratops andrewsi* | Ceraptosia | IGM 100/1021 (egg) | No | Within eggs | Norell *et al.* [6] |
| *Torvosaurus* | Megalosauridae (Theropoda) | ML1188 | No | Within eggs | Araújo *et al.* [162] |
| *Lourinhanosaurus antunesi* | Theropoda | ML565 | No | Within eggs | Mateus *et al.* [163]; Ribeiro *et al.* [74] |
| Therizinosauroidea | Therizinosauroidea (Theropoda) | CAGS-01-IG-13 | No | Within eggs | Benson *et al.* [52] |
| *Bonapartenykus ultimus* | Alvarezsauridae (Dinosauria, Theropoda) | MPCA, 1290 | Nearby eggs | No | Agnolin *et al.* [164] |
| *Triprismatoolithus stephensi* | Alvarezsauridae (Dinosauria, Theropoda) | ES 101 | No | No, identified by eggshell | Agnolin *et al.* [164] |
| ? | Oviraptorosauria (Theropoda) | SDM-008 | No | Within eggs | Shao *et al.* [165] |
| *Heyuannia huangi* | Oviraptorosauria (Theropoda) | NMNS-0015726-F02-embryo-01; CM-41 | No | Within eggs | Cheng *et al.* [166] |
| *Macroolithus* | Oviraptorosauria (Theropoda) | PFMM-0014003019 | No | No | Yang *et al.* [297] |
| *Citipati osmolskae* | Oviraptorosauria (Theropoda) | IGM 100/979; IGM 100/1004 | Atop egg clutch | No | Norell *et al.* [167]; Norell *et al.* [14] |
| *Oviraptor_philoceratops* | Oviraptorosauria (Theropoda) | IGM 100/971 (embryo) | No | Within eggs | Norell *et al.* [13] |
|  |  | AMNH 6517 (adult) and AMNH 6508 (Clutch) | Nearby eggs | No | Norell *et al.* [14] |
| *Nemegtomaia barsboldi* | Oviraptorosauria (Theropoda) | MPC-D 107/15 | Atop egg clutch | No | Fanti *et al.* [169] |
| ? | Oviraptorosauria (Theropoda) | LDNHMF2008 | Atop egg clutch | Within eggs | Bi *et al.* [54] |
| ? | Oviraptorosauria (Theropoda) | ZMNH M8829 | Atop egg clutch | No | Jin *et al.* [55] |
| *Beibeilong sinensis* (Oviraptorosauria) | Oviraptorosauria (Theropoda) | HGM 41HIII1219 | No | Within eggs | Pu *et al.* [171] |
| ? | Oviraptorosauria (Theropoda) | KNUE 10101 | No | No, identified by eggshell | Kim *et al.* [172] |
| ? | Oviraptorosauria (Theropoda) | MNHM-nat201153 | No | No, identified by eggshell | Huh *et al.* [173] |
| *Deinonychus antirrhopus* | Dromaeosauridae (Theropoda) | AMNH 3015 | Nearby eggs | No | Grellet-Tinner and Makovicky [12] |
| ? | caenagnathid or dromaeosaurid | TMP 2007.4.1 | No | No | Zelenitsky and Therrien [174] |
| *Troodon formosus* | Troodontidae (Theropoda) | MOR 246-11 | No | Within eggs | Varricchio *et al.* [298] |
| *Byronosaurus_jaffei* | Troodontidae (Theropoda) | IGM 100/972; IGM 100/974 | No | Hatchling skull with eggshell | Grellet-Tinner [176]; Varricchio and Barta [23] |
| *Almas_ukhaa* | Troodontidae (Theropoda) | IGM100/1323 | Nearby eggs | No | Pei *et al.* [177] |
| *Avimaia schweitzerae* | Enantiornithes (Avialae) | IVPP V25371(bone) | Atop eggs (unlaid egg) | No | Bailleul *et al.* [19] |
| ? | Enantiornithes (Avialae) | ZPAL MgOv-II/7a–e(Holo) | Atop eggs | No | Varricchio *et al.* [20] |
| ? | Enantiornithes (Avialae) | IGM 100/2010 | No | Within egg | Balanoff *et al.* [178]; Mikhailov [179]; |
| *Gobipipus reshetovi* | Enantiornithes (Avialae) | PIN, no. 4492/3; PIN, no. 4492/4 | No | Nearby eggshell | Kurochkin *et al.* [18] |
| *Neuquenornis volans* | Enantiornithes (Avialae) | MUCP V 37 etc. | No | Within egg | Fernández *et al.* [16] |
| *Neuquenornis volans* | Enantiornithes (Avialae) | MUCP V350-355; MUCP v-284 (embryo) | No | Within egg | Fernández *et al.* [16] |
| *Gobipteryx minuta* | Enantiornithes (Avialae) | ZPAL MgR-I/33- 34; ZPAL MgR-I/88-91 | No | Nearby eggshell | Elzanowski [17] |
| ? | Enantiornithes (Avialae) | IVPP V14238 | No | Within egg | Zhou and Zhang [21] |
| Enantiornithine (Romania) | Enantiornithes (Avialae) | ? | Near eggshell | No | Dyke *et al.* [22] |
| Phoenicopterid indet. | (Avialae) | block Vs-1 | No | Within egg | Grellet-Tinner *et al.* [24] |
| Palaeognathae (Colorado) | Ratite (Avialae) | UCM 42144/HEC 107 (holo) | No | No, identified by eggshell | Hirsch *et al.* [182] |
| *Lithornis sp.* | Palaeognathae | USNM 336570; PU 16961; MNING 2005-1 | Eggshell preserved with adult skeleton | No | Grellet-Tinner and Dyke [184] |
| Tinamidae_indet | Palaeognathae (Tinamidae) | MIM-153 | No | No, based on egg morphology | Batista *et al.* [185] |
| *Aepyornis* | Palaeognathae (Ratitae) | NG 00010595 | No | within egg | Silyn-Roberts and Sharp [186]; Balanoff and Rowe [25] |
| *Gastornis* | Gastornithidae (Avialae) | NA | No | No, identified by egg morphology | Angst *et al.* [187] |
| *Euryapteryx curtus* | Moa | LB4005 | No | No, identified by egg morphology | Gill [60]; Gill [188] |
| *Dinornis robustus (prob)* | Moa | NMNZ ME12748 | No | No, identified by egg morphology | Gill [60]; Gill [188] |
| *Pachyornis elephantopus* | Moa | PLR355 | No | No, identified by egg morphology | Gill [60]; Gill [188] |

## References

1. Pol D, Mancuso AC, Smith RM *et al.* Earliest evidence of herd-living and age segregation amongst dinosaurs. *Sci Rep*. 2021; **11**(1): 20023. doi: 10.1038/s41598-021-99176-1

2. Goloboff PA, Catalano SA. TNT version 1.5, including a full implementation of phylogenetic morphometrics. *Cladistics*. 2016; **32**(3): 221-238. doi: 10.1111/cla.12160

3. Paquette J, Reeder RJ. Single-crystal X-ray structure refinements of two biogenic magnesian calcite crystals. *Am Mineral*. 1990; **75**: 1151-1158.

4. Tanaka K, Zelenitsky DK, Therrien F. Eggshell porosity provides insight on evolution of nesting in dinosaurs. *PLOS ONE*. 2015; **10**(11): e0142829. doi: 10.1371/journal.pone.0142829

5. Moore JR, Varricchio DJ. The evolution of diapsid reproductive strategy with inferences about extinct taxa. *PLOS ONE*. 2016; **11**(7): e0158496. doi: 10.1371/journal.pone.0158496

6. Norell MA, Wiemann J, Fabbri M *et al.* The first dinosaur egg was soft. *Nature*. 2020; **583**(7816): 406-410. doi: 10.1038/s41586-020-2412-8

7. Legendre LJ, Choi S, Clarke JA. The diverse terminology of reptile eggshell microstructure and its effect on phylogenetic comparative analyses. *J Anat*. 2022; **241**(3): 641-666. doi: 10.1111/joa.13723

8. Stein K, Prondvai E, Huang T *et al.* Structure and evolutionary implications of the earliest (Sinemurian, Early Jurassic) dinosaur eggs and eggshells. *Sci Rep*. 2019; **9**(1): 4424. doi: 10.1038/s41598-019-40604-8

9. Bonaparte JF, Vince M. El hallazgo del primer nido de dinosaurios triásicos (Saurischia, Prosauropoda), Triásico Superior de Patagonia, Argentina. *Ameghiniana*. 1979; **16**: 173-182.

10. Reisz RR, Scott D, Sues H-D *et al.* Embryos of an Early Jurassic prosauropod dinosaur and their evolutionary significance. *Science*. 2005; **309**(5735): 761-764. doi: 10.1126/science.1114942

11. Varricchio DJ, Jackson F, Borkowski JJ *et al.* Nest and egg clutches of the dinosaur *Troodon formosus* and the evolution of avian reproductive traits. *Nature*. 1997; **385**(6613): 247-250. doi: 10.1038/385247a0

12. Grellet-Tinner G, Makovicky P. A possible egg of the dromaeosaur *Deinonychus antirrhopus*: phylogenetic and biological implications. *Can J Earth Sci*. 2006; **43**(6): 705-719. doi: 10.1139/e06-033

13. Norell MA, Clark JM, Demberelyin D *et al.* A theropod dinosaur embryo and the affinities of the flaming cliffs dinosaur eggs. *Science*. 1994; **266**(5186): 779-782. doi: 10.1126/science.266.5186.779

14. Norell MA, Balanoff AM, Barta DE *et al.* A second specimen of *Citipati* o*smolskae* associated with a nest of eggs from Ukhaa Tolgod, Omnogov Aimag, Mongolia. *Am Mus Novit*. 2018; **2018**(3899): 1-44, 44.

15. Chiappe LM, Coria RA, Dingus L *et al.* Sauropod dinosaur embryos from the Late Cretaceous of Patagonia. *Nature*. 1998; **396**(6708): 258-261. doi: 10.1038/24370

16. Fernández MS, García RA, Fiorelli L *et al.* A large accumulation of avian eggs from the Late Cretaceous of Patagonia (Argentina) reveals a novel nesting strategy in Mesozoic birds. *PLOS ONE*. 2013; **8**(4): e61030. doi: 10.1371/journal.pone.0061030

17. Elzanowski A. Embryonic bird skeletons from the Late Cretaceous of Mongolia. *Palaeontol Pol*. 1981; **42**: 147-179.

18. Kurochkin EN, Chatterjee S, Mikhailov KE. An embryonic enantiornithine bird and associated eggs from the Cretaceous of Mongolia. *Paleontol J*. 2013; **47**(11): 1252-1269. doi: 10.1134/S0031030113110087

19. Bailleul AM, O’Connor J, Zhang S *et al.* An Early Cretaceous enantiornithine (Aves) preserving an unlaid egg and probable medullary bone. *Nat Commun*. 2019; **10**(1): 1275. doi: 10.1038/s41467-019-09259-x

20. Varricchio DJ, Balanoff AM, Norell MA. Reidentification of avian embryonic remains from the Cretaceous of Mongolia. *PLOS ONE*. 2015; **10**(6): e0128458. doi: 10.1371/journal.pone.0128458

21. Zhou Z-H, Zhang F-C. A precocial avian embryo from the Lower Cretaceous of China. *Science*. 2004; **306**(5696): 653-653. doi: 10.1126/science.1100000

22. Dyke G, Vremir M, Kaiser G *et al.* A drowned Mesozoic bird breeding colony from the Late Cretaceous of Transylvania. *Naturwissenschaften*. 2012; **99**(6): 435-442. doi: 10.1007/s00114-012-0917-1

23. Varricchio DJ, Barta DE. Revisiting Sabath's “Larger Avian Eggs” from the Gobi Cretaceous. *Acta Palaeontol Pol*. 2015; **60** (1): 11-25.

24. Grellet-Tinner G, Murelaga X, Larrasoaña JC *et al.* The first occurrence in the fossil record of an aquatic avian twig-nest with Phoenicopteriformes eggs: evolutionary implications. *PLOS ONE*. 2012; **7**(10): e46972. doi: 10.1371/journal.pone.0046972

25. Balanoff AM, Rowe T. Osteological description of an embryonic skeleton of the extinct elephant bird, *Aepyornis* (Palaeognathae: Ratitae). *J Vert Paleontol*. 2007; **27**(sup4): 1-53. doi: 10.1671/0272-4634(2007)27[1:ODOAES]2.0.CO;2

26. Chiappe LM, Codorniú L, Grellet-Tinner G *et al.* Argentinian unhatched pterosaur fossil. *Nature*. 2004; **432**(7017): 571-572. doi: 10.1038/432571a

27. Grellet-Tinner G, Thompson MB, Fiorelli LE *et al.* The first pterosaur 3-D egg: Implications for *Pterodaustro guinazui* nesting strategies, an Albian filter feeder pterosaur from central Argentina. *Geosci Front*. 2014; **5**(6): 759-765. doi: 10.1016/j.gsf.2014.05.002

28. Ji Q, Ji S-A, Cheng Y-N *et al.* Pterosaur egg with a leathery shell. *Nature*. 2004; **432**(7017): 572-572. doi: 10.1038/432572a

29. Wang X-L, Zhou Z-H. Pterosaur embryo from the Early Cretaceous. *Nature*. 2004; **429**(6992): 621-621. doi: 10.1038/429621a

30. Unwin DM, Deeming DC. Pterosaur eggshell structure and its implications for pterosaur reproductive biology. *Zitteliana*. 2008; **B28**: 199-207.

31. Lü J-C, Unwin DM, Deeming DC *et al.* An egg-adult association, gender, and reproduction in pterosaurs. *Science*. 2011; **331**(6015): 321. doi: 10.1126/science.1197323

32. Wang X-L, Kellner AWA, Cheng X *et al.* Eggshell and histology provide insight on the life history of a pterosaur with two functional ovaries. *An Acad Bras Cienc*. 2015; **87**: 1599-1609.

33. Wang X-L, Kellner Alexander WA, Jiang S-X *et al.* Sexually dimorphic tridimensionally preserved pterosaurs and their eggs from China. *Curr Biol*. 2014; **24**(12): 1323-1330. doi: 10.1016/j.cub.2014.04.054

34. Wang Y-M, You H-L, Wang T. A new basal sauropodiform dinosaur from the Lower Jurassic of Yunnan Province, China. *Sci Rep*. 2017; **7**: 41881. doi: 10.1038/srep41881

35. Li Y, Zhu X-F, Wang Q *et al.* Apatite in *Hamipterus tianshanensis* eggshell: advances in understanding the structure of pterosaur eggs by Raman spectroscopy. *Herit Sci*. 2022; **10**(1): 84. doi: 10.1186/s40494-022-00720-7

36. Russo J, Mateus O, Marzola M *et al.* Two new ootaxa from the late Jurassic: The oldest record of crocodylomorph eggs, from the Lourinhã Formation, Portugal. *PLOS ONE*. 2017; **12**(3): e0171919. doi: 10.1371/journal.pone.0171919

37. Marsola JCdA, Batezelli A, Montefeltro FC *et al.* Palaeoenvironmental characterization of a crocodilian nesting site from the Late Cretaceous of Brazil and the evolution of crocodyliform nesting strategies. *Palaeogeogr, Palaeoclimatol, Palaeoecol*. 2016; **457**: 221-232. doi: 10.1016/j.palaeo.2016.06.020

38. Ke Y-Z, Wu R, Zelenitsky DK *et al.* A large and unusually thick-shelled turtle egg with embryonic remains from the Upper Cretaceous of China. *Proc R Soc B: Biol Sci*. 2021; **288**(1957): 20211239. doi: 10.1098/rspb.2021.1239

39. Cadena E-A, Parra-Ruge ML, Parra-Ruge JdD *et al.* A gravid fossil turtle from the Early Cretaceous reveals a different egg development strategy to that of extant marine turtles. *Palaeontology*. 2019; **62**(4): 533-545. doi: 10.1111/pala.12413

40. Hirsch KF. Parataxonomic classification of fossil chelonian and gecko eggs. *J Vert Paleontol*. 1996; **16**(4): 752-762. doi: 10.1080/02724634.1996.10011363

41. Lawver DR, Jackson FD. A fossil egg clutch from the stem turtle *Meiolania platyceps*: implications for the evolution of turtle reproductive biology. *J Vert Paleontol*. 2016; **36**(6): e1223685. doi: 10.1080/02724634.2016.1223685

42. Zhou C-F, Rabi M. A sinemydid turtle from the Jehol Biota provides insights into the basal divergence of crown turtles. *Sci Rep*. 2015; **5**(1): 16299. doi: 10.1038/srep16299

43. Fernandez V, Buffetaut E, Suteethorn V *et al.* Evidence of egg diversity in Squamate evolution from Cretaceous Anguimorph embryos. *PLOS ONE*. 2015; **10**(7): e0128610. doi: 10.1371/journal.pone.0128610

44. Hou L-H, Li P-P, Ksepka DT *et al.* Implications of flexible-shelled eggs in a Cretaceous choristoderan reptile. *Proc R Soc B: Biol Sci*. 2010; **277**(1685): 1235-1239. doi: doi:10.1098/rspb.2009.2035

45. Kusuda S, Yasukawa Y, Shibata H *et al.* Diversity in the Matrix Structure of Eggshells in the Testudines (Reptilia). *Zool Sci*. 2013; **30**(5): 366-374, 369. doi: 10.2108/zsj.30.366

46. Thorbjarnarson JB. Reproductive characteristics of the order Crocodylia. *Herpetologica*. 1996; **52**(1): 8-24.

47. Birchard GF, Ruta M, Deeming DC. Evolution of parental incubation behaviour in dinosaurs cannot be inferred from clutch mass in birds. *Biol Lett*. 2013; **9**(4): 20130036. doi: 10.1098/rsbl.2013.0036

48. Dunning Jr JB. *CRC handbook of avian body masses*. Boca Raton: CRC press, 2007.

49. Witton M. A new approach to determining pterosaur body mass and its implications for pterosaur flight. *Zitteliana*. 2008; **B28**: 143-158.

50. Jiang S-X, Zhang X-J, Cheng X *et al.* A new pteranodontoid pterosaur forelimb from the upper Yixian Formation, with a revision of *Yixianopterus jingangshanensis*. *Vert PalAsiat*. 2020; **59**(2): 81-94. doi: 10.19615/j.cnki.1000-3118.201124

51. Lü J-C. A new pterosaur: *Beipiaopterus chenianus* gen. et sp. nov. (Reptilia: Pterosauria) from western Liaoning Province of China. *Mem Fukui Prefect*. 2003; **2**: 153-160.

52. Benson RBJ, Hunt G, Carrano MT *et al.* Cope's rule and the adaptive landscape of dinosaur body size evolution. *Palaeontology*. 2018; **61**(1): 13-48. doi: 10.1111/pala.12329

53. Campione NE, Evans DC, Brown CM *et al.* Body mass estimation in non-avian bipeds using a theoretical conversion to quadruped stylopodial proportions. *Methods Ecol Evol*. 2014; **5**(9): 913-923. doi: 10.1111/2041-210X.12226

54. Bi S-D, Amiot R, Peyre de Fabrègues C *et al.* An oviraptorid preserved atop an embryo-bearing egg clutch sheds light on the reproductive biology of non-avialan theropod dinosaurs. *Sci Bull*. 2021; **66**(9): 947-954. doi: 10.1016/j.scib.2020.12.018

55. Jin X-S, Varricchio DJ, Poust AW *et al.* An oviraptorosaur adult-egg association from the Cretaceous of Jiangxi Province, China. *J Vert Paleontol*. 2019; **39**(6): e1739060. doi: 10.1080/02724634.2019.1739060

56. Alexander RM. Allometry of the leg bones of moas (Dinornithes) and other birds. *J Zool*. 1983; **200**: 215-231.

57. David JV, Daniel EB. Revisiting Sabath's “Larger Avian Eggs” from the Gobi Cretaceous. *Acta Palaeontol Pol*. 2014; **60**(1): 11-25. doi: 10.4202/app.00085.2014

58. Chiappe LM, Calvo JO. *Neuquenornis volans*, a new Late Cretaceous bird (Enantiornithes: Avisauridae) from Patagonia, Argentina. *J Vert Paleontol*. 1994; **14**(2): 230-246. doi: 10.1080/02724634.1994.10011554

59. Huynen L, Gill BJ, Millar CD *et al.* Ancient DNA reveals extreme egg morphology and nesting behavior in New Zealand’s extinct moa. *Proc Natl Acad Sci*. 2010; **107**(37): 16201. doi: 10.1073/pnas.0914096107

60. Gill BJ. A catalogue of moa eggs (Aves: Dinornithiformes). *Record Auckl Mus*. 2006; **43**: 55-80.

61. Marzola M, Russo J, Mateus O. Identification and comparison of modern and fossil crocodilian eggs and eggshell structures. *Hist Biol*. 2015; **27**(1): 115-133. doi: 10.1080/08912963.2013.871009

62. Ferguson MW. The reproductive biology and embryology of the crocodilians. In: Gans C, Billet F, Maderson PFA (eds.). *Biology of the Reptilia*. New York: John Wiley and Sons; 1985. 451-460.

63. Hoyt DF. Practical Methods of Estimating Volume and Fresh Weight of Bird Eggs. *The Auk*. 1979; **96**(1): 73-77. doi: 10.1093/auk/96.1.73

64. Wang X-L, Kellner AWA, Jiang S-X *et al.* Egg accumulation with 3D embryos provides insight into the life history of a pterosaur. *Science*. 2017; **358**(6367): 1197. doi: 10.1126/science.aan2329

65. Varricchio DJ, Jackson FD, Jackson RA *et al.* Porosity and water vapor conductance of two *Troodon formosus* eggs: an assessment of incubation strategy in a maniraptoran dinosaur. *Paleobiology*. 2013; **39**(2): 278-296. doi: 10.1666/11042

66. Chiappe LM, Coria RA, Jackson F *et al.* The Late Cretaceous nesting site of Auca Mahuevo (Patagonia, Argentina): eggs, nests, and embryos of titanosaurian sauropods. *Palaeovertebrata*. 2003; **32**(2-4): 97-108.

67. Jackson FD, Garrido A, Schmitt JG *et al.* Abnormal, multilayered titanosaur (Dinosauria: Sauropoda) eggs from in situ clutches at the Auca Mahuevo locality, Neuquen Province, Argentina. *J Vert Paleontol*. 2004; **24**(4): 913-922. doi: 10.1671/0272-4634(2004)024[0913:AMTDSE]2.0.CO;2

68. Hechenleitner EM, Fiorelli LE, Grellet-Tinner G *et al.* A new Upper Cretaceous titanosaur nesting site from La Rioja (NW Argentina), with implications for titanosaur nesting strategies. *Palaeontology*. 2016; **59**(3): 433-446. doi: 10.1111/pala.12234

69. Grellet-Tinner G, Sim CM, Kim DH *et al.* Description of the first lithostrotian titanosaur embryo in ovo with Neutron characterization and implications for lithostrotian Aptian migration and dispersion. *Gondwana Res*. 2011; **20**(2): 621-629. doi: 10.1016/j.gr.2011.02.007

70. Grellet-Tinner G, Codrea V, Folie A *et al.* First evidence of reproductive adaptation to “island effect” of a dwarf Cretaceous romanian titanosaur, with embryonic integument in ovo. *PLOS ONE*. 2012; **7**(3): e32051. doi: 10.1371/journal.pone.0032051

71. Horner JR. Egg clutches and embryos of two hadrosaurian dinosaurs. *J Vert Paleontol*. 1999; **19**(4): 607-611. doi: 10.1080/02724634.1999.10011174

72. Grigorescu D. The ‘Tuştea puzzle’ revisited: Late Cretaceous (Maastrichtian) *Megaloolithus* eggs associated with *Telmatosaurus* hatchlings in the Haţeg Basin. *Hist Biol*. 2016; **29**(5): 627-640. doi: 10.1080/08912963.2016.1227327

73. Kundrát M, Cruickshank ARI, Manning TW *et al.* Embryos of therizinosauroid theropods from the Upper Cretaceous of China: diagnosis and analysis of ossification patterns. *Acta Zool*. 2008; **89**(3): 231-251. doi: 10.1111/j.1463-6395.2007.00311.x

74. Ribeiro V, Mateus O, Holwerda F *et al.* Two new theropod egg sites from the Late Jurassic Lourinhã Formation, Portugal. *Hist Biol*. 2013; **26**(2): 206-217. doi: 10.1080/08912963.2013.807254

75. Durand JF. The oldest juvenile dinosaurs from Africa. *J African Earth Sci*. 2001; **33**(3): 597-603. doi: 10.1016/S0899-5362(01)00079-3

76. Legendre LJ, Rubilar-Rogers D, Musser GM *et al.* A giant soft-shelled egg from the Late Cretaceous of Antarctica. *Nature*. 2020; **583**(7816): 411-414. doi: 10.1038/s41586-020-2377-7

77. Ferguson MWJ. The structure and composition of the eggshell and embryonic membranes of *Alligator mississippiensis*. *Trans Zool Soc London*. 1982; **36**(2): 99-152. doi: 10.1111/j.1096-3642.1982.tb00064.x

78. Schleich H-H, Kästle W. *Reptile egg-shells SEM atlas*. Stuggart—New York: Gustav-Fischer Verlag, 1988.

79. Hirsch KF. Contemporary and Fossil Chelonian Eggshells. *Copeia*. 1983; **1983**(2): 382-397. doi: 10.2307/1444381

80. Hirsch KF, Kohring R. Crocodilian eggs from the middle Eocene Bridger Formation, Wyoming. *J Vert Paleontol*. 1992; **12**(1): 59-65. doi: 10.1080/02724634.1992.10011431

81. Tyler C. A study of the egg shells of the Anatidae. *Proc Zool Soc Lond*. 1964; **142**(4): 547-583. doi: 10.1111/j.1469-7998.1964.tb04629.x

82. Tyler C. A study of the egg shells of the Gaviiformes, Procellariiformes, Podicipitiformes and Pelecaniformes. *J Zool*. 1969; **158**(4): 395-412. doi: 10.1111/j.1469-7998.1969.tb02157.x

83. Igic B, Hauber ME, Galbraith JA *et al.* Comparison of micrometer- and scanning electron microscope-based measurements of avian eggshell thickness. *J Field Ornithol*. 2010; **81**(4): 402-410. doi: 10.1111/j.1557-9263.2010.00296.x

84. Wang S, Zhang S-k. Comparative study on the eggshell microstructures of four extant species of ratite and their significance. *Sichuan J Zool*. 2008; **27**(04): 493-496.

85. Maurer G, Portugal SJ, Cassey P. A comparison of indices and measured values of eggshell thickness of different shell regions using museum eggs of 230 European bird species. *Ibis*. 2012; **154**(4): 714-724. doi: 10.1111/j.1474-919X.2012.01244.x

86. Ar A, Rahn H, Paganelli CV. The avian egg: Mass and strength. *The Condor*. 1979; **81**(4): 331-337. doi: 10.2307/1366955

87. Reisz RR, Evans DC, Roberts EM *et al.* Oldest known dinosaurian nesting site and reproductive biology of the Early Jurassic sauropodomorph *Massospondylus*. *Proc Natl Acad Sci*. 2012; **109**(7): 2428. doi: 10.1073/pnas.1109385109

88. Prum RO, Berv JS, Dornburg A *et al.* A comprehensive phylogeny of birds (Aves) using targeted next-generation DNA sequencing. *Nature*. 2015; **526**(7574): 569-573. doi: 10.1038/nature15697

89. Feng S, Stiller J, Deng Y *et al.* Dense sampling of bird diversity increases power of comparative genomics. *Nature*. 2020; **587**(7833): 252-257. doi: 10.1038/s41586-020-2873-9

90. Lee MSY, Yates AM. Tip-dating and homoplasy: reconciling the shallow molecular divergences of modern gharials with their long fossil record. *Proc R Soc B: Biol Sci*. 2018; **285**(1881): 20181071. doi: 10.1098/rspb.2018.1071

91. Pereira AG, Sterli J, Moreira FRR *et al.* Multilocus phylogeny and statistical biogeography clarify the evolutionary history of major lineages of turtles. *Mol Phylogen Evol*. 2017; **113**: 59-66. doi: 10.1016/j.ympev.2017.05.008

92. McDonald AT, Wolfe DG, Freedman Fowler EA *et al.* A new brachylophosaurin (Dinosauria: Hadrosauridae) from the Upper Cretaceous Menefee Formation of New Mexico. *PeerJ*. 2021; **9**: e11084. doi: 10.7717/peerj.11084

93. Chapelle KEJ, Barrett PM, Botha J *et al.* *Ngwevu intloko*: a new early sauropodomorph dinosaur from the Lower Jurassic Elliot Formation of South Africa and comments on cranial ontogeny in *Massospondylus carinatus*. *PeerJ*. 2019; **7**: e7240. doi: 10.7717/peerj.7240

94. Mannion PD, Upchurch P, Schwarz D *et al.* Taxonomic affinities of the putative titanosaurs from the Late Jurassic Tendaguru Formation of Tanzania: phylogenetic and biogeographic implications for eusauropod dinosaur evolution. *Zool J Linn Soc*. 2019; **185**(3): 784-909. doi: 10.1093/zoolinnean/zly068

95. Foth C, Rauhut OWM. Re-evaluation of the Haarlem *Archaeopteryx* and the radiation of maniraptoran theropod dinosaurs. *BMC Evol Biol*. 2017; **17**(1): 236. doi: 10.1186/s12862-017-1076-y

96. Longrich NR, Martill DM, Andres B. Late Maastrichtian pterosaurs from North Africa and mass extinction of Pterosauria at the Cretaceous-Paleogene boundary. *PLoS Biol*. 2018; **16**(3): e2001663. doi: 10.1371/journal.pbio.2001663

97. Godoy PL, Benson RBJ, Bronzati M *et al.* The multi-peak adaptive landscape of crocodylomorph body size evolution. *BMC Evol Biol*. 2019; **19**(1): 167. doi: 10.1186/s12862-019-1466-4

98. Button DJ, Zanno LE. Repeated Evolution of Divergent Modes of Herbivory in Non-avian Dinosaurs. *Curr Biol*. 2020; **30**(1): 158-168.e154. doi: 10.1016/j.cub.2019.10.050

99. Nesbitt SJ. The early evolution of archosaurs: relationships and the origin of major clades. *Bull Am Mus Nat Hist*. 2011; **2011**(352): 1-292. doi: 10.1206/352.1

100. Ezcurra MD, Scheyer TM, Butler RJ. The origin and early evolution of Sauria: reassessing the Permian saurian fossil record and the timing of the crocodile-lizard divergence. *PLOS ONE*. 2014; **9**(2): e89165. doi: 10.1371/journal.pone.0089165

101. Dong W-P. *Stratigraphy (Lithostratigraphic) of Guizhou Province*. Wuhan: China University of Geosciences, 1997.

102. Xing L-D, Lockley MG, Tang D *et al.* Early Jurassic basal sauropodomorpha dominated tracks from Guizhou, China: Morphology, ethology, and paleoenvironment. *Geosci Front*. 2019; **10**(1): 229-240. doi: 10.1016/j.gsf.2018.06.001

103. Li L-Q, Wang Y-D, Kürschner WM *et al.* Palaeovegetation and palaeoclimate changes across the Triassic–Jurassic transition in the Sichuan Basin, China. *Palaeogeogr, Palaeoclimatol, Palaeoecol*. 2020; **556**: 109891. doi: 10.1016/j.palaeo.2020.109891

104. Dong Z-M. Chinese dinosaur faunas and their stratigraphic position. *J Stratigr*. 1980; **4**(4): 256-263.

105. Franceschi M, Jin X, Shi Z-Q *et al.* High-resolution record of multiple organic carbon-isotope excursions in lacustrine deposits of Upper Sinemurian through Pliensbachian (Early Jurassic) from the Sichuan Basin, China. *Geol Soc Am Bull*. 2022. doi: 10.1130/b36235.1

106. Meng F-S, Li X-B, Chen H-M. Fossil plants from Dongyuemiao Member of the Ziliujing Formation and Lower-Middle Jurassic boundary in Sichuan Basin, China. *Acta Geol Sin*. 2003; **42**(4): 525-536.

107. Xu W, Ruhl M, Jenkyns Hugh C *et al.* Carbon sequestration in an expanded lake system during the Toarcian oceanic anoxic event. *Nat Geosci*. 2017; **10**(2): 129-134. doi: 10.1038/ngeo2871

108. Mack GH, James WC, Monger HC. Classification of paleosols. *Geol Soc Am Bull*. 1993; **105**(2): 129-136. doi: 10.1130/0016-7606(1993)105<0129:Cop>2.3.Co;2

109. Reisz RR, Huang TD, Roberts EM *et al.* Embryology of Early Jurassic dinosaur from China with evidence of preserved organic remains. *Nature*. 2013; **496**(7444): 210-214. doi: 10.1038/nature11978

110. Dai X-D, Du Y-S, Ziegler M *et al.* Middle Triassic to Late Jurassic climate change on the northern margin of the South China Plate: Insights from chemical weathering indices and clay mineralogy. *Palaeogeogr, Palaeoclimatol, Palaeoecol*. 2022; **585**: 110744. doi: 10.1016/j.palaeo.2021.110744

111. Galton PM, Upchurch P. Prosauropoda. In: Weishampel DB, Dodson P, Osmólska H (eds.). *The Dinosauria (Second edition)*. Berkeley Los Angeles Landon: University of California Press; 2004. 232-258.

112. Chapelle KEJ, Choiniere JN. A revised cranial description of *Massospondylus carinatus* Owen (Dinosauria: Sauropodomorpha) based on computed tomographic scans and a review of cranial characters for basal Sauropodomorpha. *Peerj*. 2018; **6**. doi: 10.7717/Peerj.4224

113. Prieto-Marquez A, Norell MA. Redescription of a nearly complete skull of *Plateosaurus* (Dinosauria: Sauropodomorpha) from the Late Triassic of Trossingen (Germany). *Am Mus Novit*. 2011(3727): 1-58.

114. Zhang Q-N, Wang T, Yang Z-W *et al.* Redescription of the cranium of *Jingshanosaurus xinwaensis* (Dinosauria: Sauropodomorpha) from the Lower Jurassic Lufeng Formation of Yunnan Province, China. *Anat Rec*. 2020; **303**(4): 759-771. doi: 10.1002/ar.24113

115. Barrett PM, Upchurch P, Zhou X-D *et al.* The skull of *Yunnanosaurus huangi* young, 1942 (Dinosauria: Prosauropoda) from the Lower Lufeng Formation (Lower Jurassic) of Yunnan, China. *Zool J Linn Soc*. 2007; **150**(2): 319-341. doi: 10.1111/j.1096-3642.2007.00290.x

116. Barrett PM, Upchurch P, Wang X-L. Cranial osteology o*f Lufengosaurus huenei* Young (Dinosauria: Prosauropoda) from the Lower Jurassic of Yunnan, People's Republic of China. *J Vert Paleontol*. 2005; **25**(4): 806-822. doi: 10.1671/0272-4634(2005)025[0806:Coolhy]2.0.Co;2

117. Pol D, Powell JE. Skull anatomy of *Mussaurus patagonicus* (Dinosauria: Sauropodomorpha) from the Late Triassic of Patagonia. *Hist Biol*. 2007; **19**(1): 125-144.

118. Zhang Y-H, Yang Z-L. *A new complete osteology of Prosauropoda in the Lufeng Basin, Yunnan, China: Jingshanosaurus*. Kunming: Yunnan Publishing House of Science and Technology, 1995.

119. Sekiya T. A new prosauropod dinosaur from Lower Jurassic in Lufeng of Yunnan. *Global Geology*. 2010; **29**(1): 6-15.

120. Yates AM, Bonnan MF, Neveling J *et al.* A new transitional sauropodomorph dinosaur from the Early Jurassic of South Africa and the evolution of sauropod feeding and quadrupedalism. *Proc R Soc B: Biol Sci*. 2010; **277**(1682): 787-794. doi: 10.1098/rspb.2009.1440

121. Young C-C. *Yunnanosaurus huangi* Young (gen. et sp. nov.), a new Prosauropoda from the red beds at Lufeng, Yunnan. *Bull Geol Soc China*. 1942; **22**(1-2): 63-104. doi: 10.1111/j.1755-6724.1942.mp221-2005.x

122. Lü J-C, Yoshitsugu K, Li T-G *et al.* A new basal sauropod dinosaur from the Lufeng Basin, Yunnan Province, southwestern China. *Acta Geol Sin*. 2010; **84**(6): 1336-1342. doi: 10.1111/j.1755-6724.2010.00332.x

123. Otero A, Pol D. Postcranial anatomy and phylogenetic relationships of *Mussaurus patagonicus* (Dinosauria, Sauropodomorpha). *J Vert Paleontol*. 2013; **33**(5): 1138-1168. doi: 10.1080/02724634.2013.769444

124. Sekiya T, Jin XS, Zheng WJ *et al.* A new juvenile specimen of *Yunnanosaurus robustus* (Dinosauria: Sauropodomorpha) from Early to Middle Jurassic of Chuxiong Autonomous Prefecture, Yunnan Province, China. *Hist Biol*. 2014; **26**(2): 252-277. doi: 10.1080/08912963.2013.821702

125. Young CC. *Gyposaurus sinensis* Young (sp. nov.) a new Prosauropoda from the Upper Triassic Beds at Lufeng, Yunnan. *Bull Geol Soc China*. 1941; **21**(2‐4): 205-252.

126. Packard MJ, Packard GC, Boardman TJ. Structure of eggshells and water relations of reptilian eggs. *Herpetologica*. 1982; **38**(1): 136-155.

127. Choi S, Han S, Kim N-H *et al.* A comparative study of eggshells of Gekkota with morphological, chemical compositional and crystallographic approaches and its evolutionary implications. *PLOS ONE*. 2018; **13**(6): e0199496. doi: 10.1371/journal.pone.0199496

128. Wang Q, Wang X-L, Zhao Z-K *et al.* A new oogenus of Elongatoolithidae from the Upper Cretaceous Chichengshan Formation of Tiantai Basin, Zhejiang Province. *Vert PalAsiat*. 2010; **48**(2): 111-118. doi: 10.19615/j.cnki.1000-3118.2010.02.003

129. Choi S, Lee Y-N. Possible Late Cretaceous dromaeosaurid eggshells from South Korea: A new insight into dromaeosaurid oology. *Cretac Res*. 2019; **103**: 104-167. doi: 10.1016/j.cretres.2019.06.013

130. Zhang S-K, Yang T-R, Li Z-Q *et al.* New dinosaur egg material from Yunxian, Hubei Province, China resolves the classification of dendroolithid eggs. *Acta Palaeontol Pol*. 2018; **63**(4): 671-678. doi: 10.4202/app.00523.2018

131. Bonaparte JF, Vince M. El hallazgo del primer nido de dinosaurios Triásicos (Saurischia, Prosauropoda), Triásico superior de Patagonia, Argentina. *Ameghiniana*. 1979; **16**(1-2): 173-182.

132. Otero A, Cuff AR, Allen V *et al.* Ontogenetic changes in the body plan of the sauropodomorph dinosaur *Mussaurus patagonicus* reveal shifts of locomotor stance during growth. *Sci Rep*. 2019; **9**(1): 7614. doi: 10.1038/s41598-019-44037-1

133. Schwarz D, Ikejiri T, Breithaupt BH *et al.* A nearly complete skeleton of an early juvenile diplodocid (Dinosauria: Sauropoda) from the Lower Morrison Formation (Late Jurassic) of north central Wyoming and its implications for early ontogeny and pneumaticity in sauropods. *Hist Biol*. 2007; **19**(3): 225-253. doi: 10.1080/08912960601118651

134. Carballido JL, Marpmann JS, Schwarz-Wings D *et al.* New information on a juvenile sauropod specimen from the Morrison Formation and the reassessment of its systematic position. *Palaeontology*. 2012; **55**(3): 567-582. doi: 10.1111/j.1475-4983.2012.01139.x

135. Riggs ES. ART. XXX.—*Brachiosaurus altithorax*, the largest known Dinosaur. *Am J Sci*. 1903; **15**(88): 299-306.

136. Gilmore CW. *Osteology of Apatosaurus, with species reference to specimens in the Carnegie Museum*: Carnegie Institute, 1936.

137. Rogers KC. The postcranial osteology of *Rapetosaurus krausei* (Sauropoda: Titanosauria) from the Late Cretaceous of Madagascar. *J Vert Paleontol*. 2009; **29**(4): 1046-1086. doi: 10.1671/039.029.0432

138. McIntosh JS, Miller WE, Stadtman KL *et al.* The osteology of *Camarasaurus lewisi* (Jensen, 1988). *Brigham Young Univ Geol Stud*. 1996; **41**: 73-95.

139. Ikejiri T. Anatomy of *Camarasaurus lentus* (Dinosauria: Sauropoda) from the Morrison Formation (Late Jurassic), Thermopolis, central Wyoming, with determination and interpretation of ontogenetic, sexual dimorphic, and individual variation in the genus *Master*. Fort Hays State University, 2004.

140. Kirici K, Çetin O, Gunlu A *et al.* Effect of hen weight on egg production and some egg quality characteristics in pheasants (*Phasianus colchicus*). *Asian-australas J Anim Sci*. 2004; **17**(5): 684-687.

141. Dauphin Y, Cuif J-P, Salomé M *et al.* Microstructure and chemical composition of giant avian eggshells. *Anal Bioanal Chem*. 2006; **386**(6): 1761. doi: 10.1007/s00216-006-0784-8

142. Bour R, Luiselli L, Petrozzi F *et al.* *Pelusios castaneus* (Schweigger 1812)—West African mud turtle, swamp terrapin. In: Rhodin AGJ, Pritchard PCH, van Dijk PP, et al. (eds.). *Conservation biology of freshwater turtles and tortoises: a compilation project of the IUCN/SSC Tortoise and Freshwater Turtle Specialist Group Chelonian Research Monograph*2016. 095.001-095.011.

143. Youngsabanant M, Nuamsukon S. Morphology and elemental components of sea turtle eggshells using scanning electron microscopy. *Sci Eng Health Stud*. 2020; **14**(1): 73-82. doi: 10.14456/sehs.2020.7

144. Elgar MA, Heaphy LJ. Covariation between clutch size, egg weight and egg shape: comparative evidence for chelonians. *J Zool*. 1989; **219**(1): 137-152. doi: 10.1111/j.1469-7998.1989.tb02572.x

145. Sahoo G, Mohapatra BK, Sahoo RK *et al.* Ultrastructure and Characteristics of Eggshells of the Olive Ridley Turtle (*Lepidochelys olivacea*) from Gahirmatha, India. *Cells Tissues Organs*. 1996; **156**(4): 261-267. doi: 10.1159/000147854

146. Iverson JB, Heather H, Abby S *et al.* Local and geographic variation in the reproductive biology of the snapping turtle (*Chelydra serpentina*). *Herpetologica*. 1997; **53**(1): 96-117.

147. Selman W, Jones R. *Graptemys flavimaculata* Cagle 1954—yellow-blotched sawback, yellow-blotched map turtle. In: Rhodin AGJ, Pritchard PCH, van Dijk PP, et al. (eds.). *Conservation Biology of Freshwater Turtles and Tortoises: A Compilation Project of the IUCN/SSC Tortoise and Freshwater Turtle Specialist Group Chelenian Research Mongraphs*2011. 052.001-052.011.

148. Yasukawa Y, Ota H. *Geoemyda japonica* Fan 1931–Ryukyu black-breasted leaf turtle, Okinawa black-breasted leaf turtle. In: Rhodin AGJ, Pritchard PCH, van Dijk PP, et al. (eds.). *Conservation Biology of Freshwater Turtles and Tortoises: A Compilation Project of the IUCN/SSC Tortoise and Freshwater Turtle Specialist Group Chelonian Research Monographs*2008. 002.001-002.006.

149. Okada Y, Yabe T, Oda S-I. Embryonic Development of the Japanese Pond Turtle, *Mauremys japonica* (Testudines: Geoemydidae). *Curr Herpetol*. 2011; **30**(2): 89-102. doi: 10.5358/hsj.30.89

150. Oliveira CEM, Santucci RM, Andrade MB *et al.* Crocodylomorph eggs and eggshells from the Adamantina Formation (Bauru Group), Upper Cretaceous of Brazil. *Palaeontology*. 2011; **54**(2): 309-321. doi: 10.1111/j.1475-4983.2010.01028.x

151. Brazaitis P, Watanabe ME. Crocodilian behaviour: a window to dinosaur behaviour? *Hist Biol*. 2011; **23**(1): 73-90. doi: 10.1080/08912963.2011.560723

152. Blas XPI, Patnaik R. A complete crocodylian egg from the Upper Miocene (Chinji Beds) of Pakistan and its palaeobiographical implications. *PalArch's J Vertebr Palaeontol*. 2009; **6**(1): 01-08.

153. Codorniú L, Chiappe L, Rivarola D. Neonate morphology and development in pterosaurs: evidence from a ctenochasmatid embryo from the Early Cretaceous of Argentina. *Geol Soc Spec Publ*. 2018; **455**(1): 83-94. doi: 10.1144/SP455.17

154. Sander PM, Peitz C, Jackson FD *et al.* Upper Cretaceous titanosaur nesting sites and their implications for sauropod dinosaur reproductive biology. *Palaeontogr Abt A*. 2008; **284**: 69-107. doi: 10.1127/pala/284/2008/69

155. Fernández MS, Khosla A. Parataxonomic review of the Upper Cretaceous dinosaur eggshells belonging to the oofamily Megaloolithidae from India and Argentina. *Hist Biol*. 2015; **27**(2): 158-180. doi: 10.1080/08912963.2013.871718

156. Vila B, Jackson FD, Fortuny J *et al.* 3-D modelling of megaloolithid clutches: insights about nest construction and dinosaur behaviour. *PLOS ONE*. 2010; **5**(5): e10362. doi: 10.1371/journal.pone.0010362

157. Grellet-Tinner G, Fiorelli LE. A new Argentinean nesting site showing neosauropod dinosaur reproduction in a Cretaceous hydrothermal environment. *Nat Commun*. 2010; **1**(1): 32. doi: 10.1038/ncomms1031

158. Kundrát M, Coria RA, Manning TW *et al.* Specialized craniofacial anatomy of a titanosaurian embryo from Argentina. *Curr Biol*. 2020; **30**(21): 4263-4269. doi: 10.1016/j.cub.2020.07.091

159. Grigorescu D. Dinosaurs of Romania. *CR Palevol*. 2003; **2**(1): 97-101. doi: 10.1016/S1631-0683(03)00008-3

160. Grigorescu D. The “Tustea Puzzle”: Hadrosaurid (Dinosauria, Ornithopoda) hatchlings associated with Megaloolithidae eggs In the Maastrichtian of the Hateg Basin (Romania). *Ameghiniana*. 2010; **47**(1): 89-97. doi: 10.5710/AMGH.v47i1.9

161. Xing L-D, Niu K-C, Yang T-R *et al.* Hadrosauroid eggs and embryos from the Upper Cretaceous (Maastrichtian) of Jiangxi Province, China. *BMC Evol Biol*. 2022; **22**(1): 60. doi: 10.1186/s12862-022-02012-x

162. Araújo R, Castanhinha R, Martins RMS *et al.* Filling the gaps of dinosaur eggshell phylogeny: Late Jurassic Theropod clutch with embryos from Portugal. *Sci Rep*. 2013; **3**(1): 1924. doi: 10.1038/srep01924

163. Mateus I, Mateus H, Antunes M *et al.* Upper Jurassic theropod dinosaur embryos from Lourinhã (Portugal). *Mems RAcadSciLisb*. 1998; **37**: 101-109.

164. Agnolin FL, Powell JE, Novas FE *et al.* New alvarezsaurid (Dinosauria, Theropoda) from uppermost Cretaceous of north-western Patagonia with associated eggs. *Cretac Res*. 2012; **35**: 33-56. doi: 10.1016/j.cretres.2011.11.014

165. Shao Z, Fan S, Jia S *et al.* Intact theropod dinosaur eggs with embryonic remains from the Late Cretaceous of southern China. *Geol Bull China*. 2014; **33**: 941-948.

166. Cheng Y-N, Ji Q, Wu X-C *et al.* Oviraptorosaurian eggs (Dinosauria) with embryonic skeletons discovered for the first time in China. *Acta Geol Sin*. 2008; **82**(6): 1089-1094. doi: 10.1111/j.1755-6724.2008.tb00708.x

167. Norell MA, Clark JM, Chiappe LM *et al.* A nesting dinosaur. *Nature*. 1995; **378**(6559): 774-776. doi: 10.1038/378774a0

168. Osborn HF, Kaisen PC, Olsen G. Three new theropoda, protoceratops zone, central Mongolia. *Am Mus Novit*. 1924(144): 1-12.

169. Fanti F, Currie PJ, Badamgarav D. New Specimens of *Nemegtomaia* from the Baruungoyot and Nemegt Formations (Late Cretaceous) of Mongolia. *PLOS ONE*. 2012; **7**(2): e31330. doi: 10.1371/journal.pone.0031330

170. Lü J, Chen R, Brusatte SL *et al.* A Late Cretaceous diversification of Asian oviraptorid dinosaurs: evidence from a new species preserved in an unusual posture. *Sci Rep*. 2016; **6**(1): 35780. doi: 10.1038/srep35780

171. Pu H, Zelenitsky DK, Lü J *et al.* Perinate and eggs of a giant caenagnathid dinosaur from the Late Cretaceous of central China. *Nat Commun*. 2017; **8**(1): 14952. doi: 10.1038/ncomms14952

172. Kim J, Yang S, Choi H *et al.* Dinosaur eggs from the Cretaceous Goseong Formation of Tongyeong City, Southern Coast of Korea. *J Geol Soc Korea*. 2011; **27**: 13-26.

173. Huh M, Kim BS, Woo Y *et al.* First record of a complete giant theropod egg clutch from Upper Cretaceous deposits, South Korea. *Hist Biol*. 2014; **26**(2): 218-228. doi: 10.1080/08912963.2014.894998

174. Zelenitsky DK, Therrien F. Unique maniraptoran egg clutch from the Upper Cretaceous Two Medicine Formation of Montana reveals theropod nesting behaviour. *Palaeontology*. 2008; **51**(6): 1253-1259. doi: 10.1111/j.1475-4983.2008.00815.x

175. Vila B, Sellés AG, Beetschen J-C. The controversial Les Labadous eggshells: A new and peculiar dromaeosaurid (Dinosauria: Theropoda) ootype from the Upper Cretaceous of Europe. *Cretac Res*. 2017; **72**: 117-123. doi: 10.1016/j.cretres.2016.12.010

176. Grellet-Tinner G. *A phylogenetic analysis of oological characters: A case study of saurischian dinosaur relationships and avian evolution*: University of Southern California, 2005.

177. Pei R, Norell MA, Barta DE *et al.* Osteology of a new Late Cretaceous troodontid specimen from Ukhaa Tolgod, Ömnögovi Aimag, Mongolia. *Am Mus Novit*. 2017; **2017**(3889): 1-47, 47.

178. Balanoff AM, Norell MA, Grellet-Tinner G *et al.* Digital preparation of a probable neoceratopsian preserved within an egg, with comments on microstructural anatomy of ornithischian eggshells. *Naturwissenschaften*. 2008; **95**(6): 493-500.

179. Mikhailov KE. Eggshell structure, parataxonomy and phylogenetic analysis: some notes on articles published from 2002 to 2011. *Hist Biol*. 2014; **26**(2): 144-154. doi: 10.1080/08912963.2013.829824

180. Mikhailov KE. Classification of fossil eggshells of amniotic vertebrates. *Acta Palaeontol Pol*. 1991; **36**(2): 193-238.

181. Elzanowski A. Palaeognathous bird from the Cretaceous of Central Asia. *Nature*. 1976; **264**(5581): 51-53. doi: 10.1038/264051a0

182. Hirsch KF, Kihm AJ, Zelenitsky DK. New eggshell of ratite morphotype with predation marks from the Eocene of Colorado. *J Vert Paleontol*. 1997; **17**(2): 360-369. doi: 10.1080/02724634.1997.10010980

183. Leonard L, Dyke GJ, Van Tuinen M. A new specimen of the fossil Palaeognath *Lithornis* from the Lower Eocene of Denmark. *Am Mus Novit*. 2005; **2005**(3491): 1-11.

184. Grellet-Tinner G, Dyke G. The eggshell of the Eocene bird *Lithornis*. *Acta Palaeontol Pol*. 2005; **50**: 831-835.

185. Batista A, Jones WW, Rinderknecht A. The first complete fossil avian egg from the Quaternary of South America. *J South Am Earth Sci*. 2021; **109**: 103244. doi: 10.1016/j.jsames.2021.103244

186. Silyn-Roberts H, Sharp RM. Preferred orientation of calcite in the Aepyornis eggshell. *J Zool*. 1986; **208**(3): 475-478. doi: 10.1111/j.1469-7998.1986.tb01909.x

187. Angst D, Buffetaut E, LÉCuyer C *et al.* Fossil avian eggs from the Palaeogene of southern France: new size estimates and a possible taxonomic identification of the egg-layer. *Geol Mag*. 2014; **152**(1): 70-79. doi: 10.1017/S0016756814000077

188. Gill BJ. Eggshell characteristics of moa eggs (Aves: Dinornithiformes). *J R Soc N Z*. 2007; **37**(4): 139-150. doi: 10.1080/03014220709510542

189. Schönwetter M. *Handbuch der Oologie*. Berlin: Akademie Verlag, 1960–1992.

190. Hauber ME. *The book of eggs: a life-size guide to the eggs of six hundred of the world's bird species*: University of Chicago Press, 2014.

191. Vieco-Galvez D, Castro I, Morel PCH *et al.* The eggshell structure in *Apteryx;* form, function, and adaptation. *Ecol Evol*. 2021; **11**(7): 3184-3202. doi: 10.1002/ece3.7266

192. McLennan JA. Breeding of north island brown kiwi, *Apteryx australis mantelli*, in hawke's bay, New Zealand. *N Z J Ecol*. 1988; **11**: 89-97.

193. Sales J. The endangered kiwi: a review. *Folia Biol (Praha)*. 2005; **54**(1/2): 1.

194. Grellet-Tinner G. Phylogenetic interpretation of eggs and eggshells: implications for phylogeny of Palaeognathae. *Alcheringa*. 2006; **30**(1): 141-182. doi: 10.1080/03115510608619350

195. Arias JL, Matthei A, Valenzuela C. Exploratory and descriptive study on nutritional characteristics and quality of eggs from Chilean partridge (*Nothoprocta perdicaria*). *Anim Sci J*. 2018; **89**(1): 186-192. doi: 10.1111/asj.12900

196. Jetz W, Sekercioglu CH, Böhning-Gaese K. The worldwide variation in avian clutch size across species and space. *PLoS Biol*. 2008; **6**(12): e303. doi: 10.1371/journal.pbio.0060303

197. Booth DT. Ecological physiology of Malleefowl *(Leipoa ocellata*) *Doctor of Philosophy*. Ecological physiology of Malleefowl (Leipoa ocellata), 1985.

198. Brickhill J. Breeding success of Malleefowl *Leipoa ocellata* in Central New South Wales *Emu*. 1987; **87**(1): 42-45. doi: 10.1071/MU9870042

199. Héctor A, Roberto ARU. Biología Reproductiva del Hocofaisan *Crax rubra rubra* Linnaeus 1758, (Craciformes: Cracidae) en México, Análisis Químico y Estudio Morfológico de la Cáscara de Huevo. *Zoocriaderos*. 2002; **4**(2): 1-33.

200. Martínez-Morales MA, Cruz PC, Cuarón AD. Predicted population trends for Cozumel Curassows (*Crax rubra griscomi*): empirical evidence and predictive models in the face of climate change. *J Field Ornithol*. 2009; **80**(4): 317-327. doi: 10.1111/j.1557-9263.2009.00237.x

201. Piccirillo VJ, Orlando DA. Reproductive capacities of control bobwhite quail (*Colinus virginianus*) during one-generation reproduction studies. *J Environ Pathol Toxicol Oncol*. 1985; **6**(1): 79-84.

202. Fox L, Moreno A, Bradley G. Mortality of four captive-born crested screamer chicks (*Chauna torquata*). *Open Vet J*. 2019; **9**(2): 120-125.

203. Bingol SA, Deprem T, Karadag Sari E *et al.* Comparison between Goose (*Anser anser*) and Chicken (*Gallus gallus domesticus*) Eggshells during embryonic development by scanning electron microscopy. *Kafkas Univ Vet Fak Derg*. 2016; **22**(6): 937-943. doi: 10.9775/kvfd.2016.15668

204. Hirschenhauser K, Mostl E, Kotrschal K. Within-pair testosterone covariation and reproductive output in Greylag Geese *Anser anser*. *Ibis*. 1999; **141**(4): 577-586. doi: 10.1111/j.1474-919X.1999.tb07365.x

205. Zelenitsky DK, Hills LV, Currie PJ. Parataxonomic classification of ornithoid eggshell fragments from the Oldman Formation (Judith River Group; Upper Cretaceous), southern Alberta. *Can J Earth Sci*. 1996; **33**(12): 1655-1667. doi: 10.1139/e96-126

206. Berry R. Nightjar habitats and breeding in East Anglia. *Br Birds*. 1979; **72**(11): 207-218.

207. Grant GS. Avian incubation: egg temperature, nest humidity, and behavioral thermoregulation in a hot environment. *Ornithol Monogr*. 1982(30): iii-75. doi: 10.2307/40166669

208. Cestari C. Nesting of the Lesser Nighthawk (*Chordeiles acutipennis*) in eastern Amazonia. *Ararajuba*. 2010; **18**(18): 133-135.

209. Payevsky V. A comparison of body size in Cuckoos *Cuculus canorus* from the British Isles and the eastern Baltic area. *Ringing Migr*. 1998; **19**. doi: 10.1080/03078698.1998.9674164

210. David A, Stermin A, Sevianu E. Clutch size and egg repeatability in three elusive bird species: Little Bittern *(Ixobrychus minutus*), Little Crake *(Zapornia parva*) and Water Rail (*Rallus aquaticus*) from north-west Romanian populations. *Stud Univ Babes Bolyai Biol*. 2018; **63**: 81-88. doi: 10.24193/subbbiol.2018.1.07

211. Mikhailov K. Avian eggshells: An atlas of scanning electron micrographs. *Bull Br Orn Club*. 1997; **3**: 1-88.

212. Kirikçı K, Günlü A, Çetin O *et al.* Effect of hen weight on egg production and some egg quality characteristics in the partridge (*Alectoris graeca*). *Poult Sci*. 2007; **86**(7): 1380-1383. doi: 10.1093/ps/86.7.1380

213. Tyler C. A study of the egg shells of the Falconiformes. *J Zool*. 1966; **150**(4): 413-425. doi: 10.1111/j.1469-7998.1966.tb03015.x

214. Hustler K, Howells WW. Habitat preference, breeding success and the effect of primary productivity on Tawny Eagles *Aquila rapax* in the tropics. *Ibis*. 1989; **131**(1): 33-40. doi: 10.1111/j.1474-919X.1989.tb02741.x

215. Exo K-M. Population ecology of Little Owls *Athene noctua* in Central Europe: a review. In: Galbraith CA, Taylor IR, Percival S (eds.). *The ecology and conservation of European owls*. Peterborough: Joint Nature Conservation Committee (UK Nature Conservation, No. 5); 1992. 64-75.

216. Andreychev AV, Lapshin AS, Kuznetsov VA. Breeding success of the Eurasian eagle owl (*Bubo bubo*) and rodent population dynamics. *Biol Bull*. 2016; **43**(8): 851-861. doi: 10.1134/S1062359016080045

217. Iezekiel S, Yosef R, Bakaloudis DE *et al.* Breeding ecology of the Long-legged Buzzard (*Buteo rufinus*) in an increasing population on Cyprus. *J Arid Environ*. 2016; **135**: 12-16. doi: 10.1016/j.jaridenv.2016.08.007

218. Adamou A-E, Tabib R, Kouidri M *et al.* Inter-annual variation in clutch size and laying date of rufous bush chats *Cercotrichas galactotes* inhabiting an Algerian oasis. *J Arid Environ*. 2017; **141**: 40-44. doi: 10.1016/j.jaridenv.2017.01.013

219. Adamou A-E, Tabib R, Kouidri M *et al.* Egg size and shape variation in rufous bush chats *Cercotrichas galactotes* breeding in date palm plantations: hatching success increases with egg elongation. *Avian Biol Res*. 2018; **11**(2): 100-107. doi: 10.3184/175815618x1520281116334

220. Goldstein H, Yom-Tov Y. Breeding biology of the orange-tufted sunbird in Israel. *Ardea*. 1988; **76**: 169-174.

221. Dolenec Z. Nest density, clutch size and egg dimensions of the hooded crow (*Corvus corone cornix*). *Nat Croat*. 2006; **15**: 231-235.

222. Prashant JJ, Rao VV, Nagulu V. Nesting, egg size, incubation and factors affecting clutch size in little egret *Egretta Garzetta* at Nellore Andhra Pradesh. *Pavo*. 1994; **32**: 67-72.

223. Burnham W, Sandfort C, Belthoff JR. Peregrine falcon eggs: Egg size, hatchling sex, and clutch sex ratios. *The Condor*. 2003; **105**(2): 327-335. doi: 10.1093/condor/105.2.327

224. Samraoui F, Alfarhan AH, Samraoui B. Status and breeding ecology of the Common Moorhen *Gallinula chloropus* in Algeria. *Ostrich*. 2013; **84**(2): 137-144. doi: 10.2989/00306525.2013.823130

225. Apuno A, Mbap S, Ibrahim T. Characterization of local chickens (*Gallus gallus domesticus*) in shelleng and song local government areas of Adamawa State, Nigeria. *Agr Biol J N Am*. 2011; **2**(1): 6-14.

226. Bensaci E, Boutera N, Cherief A *et al.* Breeding ecology studies of Collared Pratincoles *Glareola pratincola i*n the Central Hauts Plateaux of Algeria. *Wader Study Group Bull*. 2014; **121**(1): 43-48.

227. Mamedova Y, Chaplygina A. Breeding of black-winged stilt *Himantopus himantopus* in muddy sites of a wastewater treatment plant. *Biosyst Divers*. 2021; **29**(3): 286-293. doi: 10.15421/012136

228. Harris T, Franklin K. *Shrikes and Bush-shrikes: Including wood-shrikes, helmet-shrikes, shrike flycatchers, philentomas, batises and wattle-eyes*: A&C Black, 2000.

229. Yom-Tov Y, Ar A. On the breeding ecology of the dead sea sparrow, *Passer moabiticus*. *Isr J Zool*. 1980; **29**(4): 171-187. doi: 10.1080/00212210.1980.10688497

230. Jamadi M, Darvishi K. Breeding Study of the dead sea sparrow *Passer moabiticus* in the mond protected area, Bushehr, Persian Gulf. *Podoces*. 2008; **3**(1/2): 97-131.

231. Sander PM. The norian *Plateosaurus* bonebeds of central Europe and their taphonomy. *Palaeogeogr, Palaeoclimatol, Palaeoecol*. 1992; **93**(3): 255-299. doi: 10.1016/0031-0182(92)90100-J

232. Robertson HA. Breeding of Collared Doves *Streptopelia decaocto* in rural Oxfordshire, England. *Bird Study*. 1990; **37**(2): 73-83. doi: 10.1080/00063659009477043

233. Cate CT, Hilbers J. Effects of brood size on inter-clutch intervals, offspring development and male-female interactions in the ring dove *Streptopelia risoria*. *Anim Behav*. 1991; **41**(1): 27-36. doi: 10.1016/S0003-3472(05)80500-7

234. Hubbard NE. *The eggshell structures of ringed turtle-doves (Streptopelia risoria)*: The University of Texas at Arlington, 2005.

235. Valitutto MT, Newton AL, Wetzlich S *et al.* Pharmacokinetics and clinical safety of a sustained-release formulation of ceftiofur crystalline free acid in ringneck doves (*Streptopelia risoria*) after a single intramuscular injection. *J Zoo Wildl Med*. 2021; **52**(1): 81-89. doi: 10.1638/2019-0122

236. Alam MS, Khan MAR, Ismail S *et al.* Breeding biology of the European Turtle Dove *Streptopelia turtur arenicola* in Dubai, United Arab Emirates. *Sandgrouse*. 2019; **41**: 186-194.

237. Vogrin M. Egg size of the Blackbird (*Turdus merula*) in Slovenia. *Butll Grup Català Anellam*. 1997: 37-41.

238. Zeraoula A, Bensouilah T, Brahmia H *et al.* Breeding biology of the European Blackbird *Turdus merula* in orange orchards. *J King Saud Univ Sci*. 2016; **28**(4): 300-307. doi: 10.1016/j.jksus.2015.10.005

239. Salim H, Noor HM, Hamid NH *et al.* The effects of rodenticide residues deposited in eggs of *Tyto alba* to eggshell thickness. *Sains Malays*. 2015; **44**(4): 559-564.

240. Sunter G. Management and reproduction of the Komodo dragon *Varanus komodoensis* Ouwens 1912 at ZSL London Zoo. *Int Zoo Yearb*. 2008; **42**(1): 172-182. doi: 10.1111/j.1748-1090.2007.00029.x

241. Heideman NJL. Reproduction in *Agama aculeata aculeata* and *Agama planiceps planiceps* females from Windhoek, Namibia. *Amphib - Reptil*. 1994; **15**(4): 351-361. doi: 10.1163/156853894X00399

242. Hamlett GW. Notes on breeding and reproduction in the lizard *Anolis carolinensis*. *Copeia*. 1952; **1952**(3): 183-185. doi: 10.2307/1439705

243. Meiri S, Avila L, Bauer AM *et al.* The global diversity and distribution of lizard clutch sizes. *Global Ecol Biogeogr*. 2020; **29**(9): 1515-1530. doi: 10.1111/geb.13124

244. Silva KMP, Silva KB, Sueiro LR *et al.* Reproductive Biology of *Bothrops atrox* (Serpentes, Viperidae, Crotalinae) from the Brazilian Amazon. *Herpetologica*. 2019; **75**(3): 198-207. doi: 10.1655/d-18-00023

245. Hill Iii JG, Chanhome L, Artchawakom T *et al.* Nest attendance by a female Malayan pit viper (*Calloselasma rhodostoma)* in northeast Thailand. *Trop Nat Hist*. 2006; **6**(2): 57-66.

246. Rosen PC. Comparative ecology and life history of the racer (*Coluber constrictor*) in Michigan. *Copeia*. 1991; **1991**(4): 897-909. doi: 10.2307/1446085

247. Diller LV, Wallace RL. Reproductive biology of the northern pacific rattlesnake (*Crotalus viridis oreganus*) in northern idaho. *Herpetologica*. 1984; **40**(2): 182-193.

248. Goldberg SR, Kraus F. Notes on reproduction of *Cryptoblepharus poecilopleurus*  (Squamata: Scincidae) from the Northern Mariana Islands, Western Pacific. *Curr Herpetol*. 2011; **30**(2): 159-161. doi: 10.5358/hsj.30.159

249. Clark DR, Bunck CM, Hall RJ. Female Reproductive Dynamics in a Maryland Population of Ringneck Snakes (*Diadophis punctatus*). *J Herpetol*. 1997; **31**(4): 476-483. doi: 10.2307/1565598

250. Rhen T, Crews D, Fivizzani A *et al.* Reproductive tradeoffs and yolk steroids in female leopard geckos, *Eublepharis macularius*. *J Evol Biol*. 2006; **19**(6): 1819-1829. doi: 10.1111/j.1420-9101.2006.01180.x

251. LaDage LD, Gutzke WHN, Simmons II RA *et al.* Multiple mating increases fecundity, fertility and relative clutch mass in the female leopard gecko (*Eublepharis macularius*). *Ethology*. 2008; **114**(5): 512-520. doi: 10.1111/j.1439-0310.2008.01495.x

252. O’Shea M, Halliday T. *Smithsonian Handbooks: Reptiles and Amphibians*: New York: Dorling and Kindersley, 2002.

253. Peet-Paré CA, Blouin-Demers G. Female eastern hog-nosed snakes (*Heterodon platirhinos*) choose nest sites that produce offspring with phenotypes likely to improve fitness. *Can J Zool*. 2012; **90**(10): 1215-1220. doi: 10.1139/z2012-091

254. Alvarado J, Ibarra L, Suazo I *et al.* Reproductive characteristics of a green iguana (*Iguana iguana*) population of the west coast of Mexico. *Southwest Nat*. 1995; **40**(2): 234-237.

255. Werneck FDP, Giugliano LG, Collevatti RG *et al.* Phylogeny, biogeography and evolution of clutch size in South American lizards of the genus *Kentropyx (*Squamata: Teiidae). *Mol Ecol*. 2009; **18**(2): 262-278. doi: 10.1111/j.1365-294X.2008.03999.x

256. Braña F, Bea A, Arrayago MJ. Egg retention in lacertid lizards: Relationships with reproductive ecology and the evolution of viviparity. *Herpetologica*. 1991; **47**(2): 218-226.

257. Goldberg SR. Reproduction in the Sonoran Whipsnake, *Masticophis bilineatus* (Serpentes: Colubridae). *Southwest Nat*. 1998; **43**(3): 412-415.

258. Goldberg SR. Reproduction in the coachwhip, *Masticophis flagellum* (Serpentes: Colubridae), from Arizona. *Tex J Sci*. 2002; **54**(2): 143-150.

259. Mollov I. Comparison of the eggs size between two subspecies of the Kotschy’s Gecko *Mediodactylus kotschyi* (Steindachner, 1870)(Reptilia: Gekkonidae) in Bulgaria. *ZooNotes*. 2011; **19**: 1-4.

260. Solórzano A, Cerdas L. Incubación de los huevos y nacimiento en la coral gargantilla, *Micrurus mipartitus hertwigi* (Serpentes: Elapidae) en Costa Rica. *Rev Biol Trop*. 1988; **36**(2B): 535-536.

261. Haagner G, Carpenter G. Notes on the reproduction of captive forest cobras, *Naja melanoleuca* (Serpentes: Elapidae). *J Herpetol Assoc Afr*. 1988; **34**(1): 35-37.

262. Bird WM, Peak P, Baxley DL. Natural history and meristics of an allopatric population of red cornsnakes, *Pantherophis guttatus* (Linnaeus, 1766) in Central Kentucky, USA. *J Herpetol*. 2015(1): 6-11. doi: 10.17161/jnah.vi1.11899

263. Demeter BJ. Observations on the care, breeding and behaviour of the giant day gecko *Phelsuma madagascariensis* at the National Zoological Park, Washington. *Int Zoo Yearb*. 1976; **16**: 130-133.

264. Ballinger RE. Reproduction of the Texas Horned Lizard, *Phrynosoma cornutum*. *Herpetologica*. 1974; **30**(4): 321-327.

265. Iverson JB, Young CA, Akre TSB *et al.* Reproduction by female bullsnakes (*Pituophis catenifer sayi* ) in the Nebraska Sandhills. *Southwest Nat*. 2012; **57**(1): 58-73, 16. doi: 10.1894/0038-4909-57.1.58

266. Werner YL. Egg size and egg shape in near-eastern gekkonid lizards. *Isr J Ecol Evol*. 1989; **35**(4): 199-213. doi: 10.1080/00212210.1988.10688614

267. Vitt LJ, Ohmart RD. Reproduction and ecology of a colorado river population of *Sceloporus magister* (Sauria: Iguanidae). *Herpetologica*. 1974; **30**(4): 410-417.

268. Ballinger RE, Droge DL, Jones SM. Reproduction in a Nebraska Sandhills Population of the Northern Prairie Lizard *Sceloporus undulatus garmani*. *Am Midl Nat*. 1981; **106**(1): 157-164. doi: 10.2307/2425145

269. Cree A, Guillette Jr. LJ, Reader K. Eggshell formation during prolonged gravidity of the tuatara *Sphenodon punctatus*. *J Morphol*. 1996; **230**(2): 129-144. doi: 10.1002/(SICI)1097-4687(199611)230:2<129::AID-JMOR2>3.0.CO;2-C

270. Nelson NJ, Thompson MB, Pledger S *et al.* Egg mass determines hatchling size, and incubation temperature influences post-hatching growth, of tuatara *Sphenodon punctatus*. *J Zool*. 2004; **263**(1): 77-87. doi: 10.1017/S095283690400490X

271. Fuentes-Fernandez MD, Suarezrancel MM, Molina-Borja M. Variation in body size and morphometric traits of males and females of the wall gecko, *Tarentola delalandii,* (Phyllodactylidae) from different environments on Tenerife. *Afr J Herpetol*. 2016; **65**(2): 83-98. doi: 10.1080/21564574.2016.1234512

272. Marques V, Riaño G, Carretero MA *et al.* Sex determination and optimal development in the Moorish gecko, *Tarentola mauritanica*. *Acta Zool*. 2022; **n/a**(n/a): 1-15. doi: 10.1111/azo.12427

273. Herrera EA, Robinson MD. Reproductive and fat body cycles of the tegu lizard, *Tupinambis teguixin*, in the Llanos of Venezuela. *J Herpetol*. 2000; **34**(4): 598-601. doi: 10.2307/1565277

274. Martin RF. Variation in reproductive productivity of range margin tree lizards (*Urosaurus ornatus*). *Copeia*. 1977; **1977**(1): 83-92. doi: 10.2307/1443508

275. Hoddenbach GA, Turner FB. Clutch Size of the Lizard Uta stansburiana in Southern Nevada. *Am Midl Nat*. 1968; **80**(1): 262-265. doi: 10.2307/2423613

276. Zhou T. *Atlas for identification of turtles and tortoises (In Chinese)*. Beijing: China Agriculture Press, 2004.

277. Williams CJA, Greunz EM, Ringgaard S *et al.* Magnetic resonance imaging (MRI) reveals high cardiac ejection fractions in red-footed tortoises (*Chelonoidis carbonarius*). *J Exp Biol*. 2019; **222**(18). doi: 10.1242/jeb.206714

278. de Miranda EBP. The plight of reptiles as ecological actors in the tropics. *Front Ecol Evol*. 2017; **5**. doi: 10.3389/fevo.2017.00159

279. Zhou T, Zhou F-T. *Tortoises of the World*. Beijing: China Agriculture Press, 2020.

280. Pritchard PCH. *Chelus fimbriata* (Schneider 1783)–matamata turtle. *Chelonian Res Monogr*. 2008; **5**(1): 020.021-020.010. doi: 10.3854/crm.5.020.fimbriata.v1.2008

281. Andersen SK, Staerk J, Kalhor E *et al.* Economics, life history and international trade data for seven turtle species in Indonesian and Malaysian farms. *Data Br*. 2021; **34**: 106708. doi: 10.1016/j.dib.2020.106708

282. Congdon JD, Greene JL, Gibbons JW. Biomass of Freshwater Turtles: A Geographic Comparison. *Am Midl Nat*. 1986; **115**(1): 165-173. doi: 10.2307/2425846

283. Márquez M. Status and distribution of the Kemp’s Ridley Turtle, *Lepidochelys kempii,* in the Wider Caribbean Region. In: Eckert KL, Grobois FAA (eds.). *Marine Turtle Conservation in the Wider Caribbean Region: A Dialogue for Effective Regional Management*. Santo Domingo, Dominican Republic: WIDECAST, IUCN-MTSG, WWF, and UNEP-CEP. ; 2001. 46-51.

284. Pritchard PCH, Mortimer JA. Taxonomy, external morphology, and species identification. In: Eckert KL, Bjorndal KA, Abreu-Grobois FA, et al. (eds.). *Research and management techniques for the conservation of sea turtles*: IUCN/SSC Marine Turtle Specialist Group Publication; 1999. 1-18.

285. Pages T, Fuster JF, Palacios L. Thermal responses of the fresh water turtle *Mauremys caspica* to step-function changes in the ambient temperature. *J Therm Biol*. 1991; **16**(6): 337-343. doi: 10.1016/0306-4565(91)90060-F

286. Holt S, Horwitz LK, Wilson B *et al.* Leopard tortoise *Stigmochelys pardalis* (Bell, 1928) mortality caused by electrified fences in central South Africa and its impact on tortoise demography. *Afr J Herpetol*. 2021; **70**(1): 32-52. doi: 10.1080/21564574.2020.1860140

287. Costanzo JP, Claussen DL. Natural freeze tolerance in the terrestrial turtle, *Terrapene carolina*. *J Exp Zool*. 1990; **254**(2): 228-232. doi: 10.1002/jez.1402540215

288. Lambert MRK. Studies on the growth, structure and abundance of the Mediterranean spur-thighed tortoise, Tesudo graeca in field populations. *J Zool*. 1982; **196**(2): 165-189. doi: 10.1111/j.1469-7998.1982.tb03499.x

289. Willemsen RE, Hailey A. Variation of adult body size of the tortoise Testudo hermanni in Greece: proximate and ultimate causes. *J Zool*. 1999; **248**(3): 379-396. doi: 10.1111/j.1469-7998.1999.tb01037.x

290. Gaio C, Rossi T, Villa R *et al.* Pharmacokinetics of Acyclovir after a single oral administration in marginated tortoises, *Testudo marginata*. *J Herpetol Med Surg*. 2007; **17**(1): 8-11. doi: 10.5818/1529-9651.17.1.8

291. Burghardt GM, Ward B, Rosscoe R. Problem of reptile play: Environmental enrichment and play behavior in a captive Nile soft‐shelled turtle, *Trionyx triunguis*. *Zoo Biol*. 1996; **15**(3): 223-238.

292. Lawver DR, Jackson FD. A review of the fossil record of turtle reproduction: eggs, embryos, nests and copulating pairs. *Bull Peabody Mus Nat Hist*. 2014; **55**(2): 215-236. doi: 10.3374/014.055.0210

293. Xu L, Xie J-F, Zhang S-K *et al.* Fossil turtle eggs from the Upper Cretaceous Gaogou Formation, Xiaguan-Gaoqiu Basin, Neixiang County, Henan Province, China: Interpretation of the transformation from aragonite to calcite in fossil turtle eggshell. *Cretac Res*. 2022; **134**: 105166. doi: 10.1016/j.cretres.2022.105166

294. Zelenitsky DK, Therrien F, Joyce WG *et al.* First fossil gravid turtle provides insight into the evolution of reproductive traits in turtles. *Biol Lett*. 2008; **4**(6): 715-718. doi: doi:10.1098/rsbl.2008.0395

295. Winkler JD, SÁnchez-Villagra MR. A nesting site and egg morphology of a Miocene turtle from Urumaco, Venezuela: Evidence of marine adaptations in pelomedusoides. *Palaeontology*. 2006; **49**(3): 641-646. doi: 10.1111/j.1475-4983.2006.00557.x

296. Erickson GM, Zelenitsky DK, Kay DI *et al.* Dinosaur incubation periods directly determined from growth-line counts in embryonic teeth show reptilian-grade development. *Proc Natl Acad Sci*. 2017; **114**(3): 540-545. doi: 10.1073/pnas.1613716114

297. Yang T-R, Wiemann J, Xu L *et al.* Reconstruction of oviraptorid clutches illuminates their unique nesting biology. *Acta Palaeontol Pol*. 2019; **64**(3): 581-596. doi: 10.4202/app.00497.2018

298. Varricchio DJ, Horner JR, Jackson FD. Embryos and eggs for the Cretaceous theropod dinosaur *Troodon formosus*. *J Vert Paleontol*. 2002; **22**(3): 564-576. doi: 10.1671/0272-4634(2002)022[0564:EAEFTC]2.0.CO;2
